# The Keloid Disorder: Heterogeneity, Histopathology, Mechanisms and Models

**DOI:** 10.3389/fcell.2020.00360

**Published:** 2020-05-26

**Authors:** Grace C. Limandjaja, Frank B. Niessen, Rik J. Scheper, Susan Gibbs

**Affiliations:** ^1^Department of Molecular Cell Biology and Immunology, Amsterdam University Medical Center (location VUmc), Vrije Universiteit Amsterdam, Amsterdam, Netherlands; ^2^Department of Plastic Surgery, Amsterdam University Medical Center (location VUmc), Vrije Universiteit Amsterdam, Amsterdam, Netherlands; ^3^Department of Pathology, Amsterdam University Medical Center (location VUmc), Vrije Universiteit Amsterdam, Amsterdam, Netherlands; ^4^Department of Oral Cell Biology, Academic Centre for Dentistry (ACTA), University of Amsterdam and Vrije Universiteit Amsterdam, Amsterdam, Netherlands

**Keywords:** keloid, cicatrix, hypertrophic, keloid anatomy and histology, keloid etiology, keloid pathology, keloid heterogeneity, keloid model, keloid phenotype

## Abstract

Keloids constitute an abnormal fibroproliferative wound healing response in which raised scar tissue grows excessively and invasively beyond the original wound borders. This review provides a comprehensive overview of several important themes in keloid research: namely keloid histopathology, heterogeneity, pathogenesis, and model systems. Although keloidal collagen versus nodules and α-SMA-immunoreactivity have been considered pathognomonic for keloids versus hypertrophic scars, conflicting results have been reported which will be discussed together with other histopathological keloid characteristics. Importantly, histopathological keloid abnormalities are also present in the keloid epidermis. Heterogeneity between and within keloids exists which is often not considered when interpreting results and may explain discrepancies between studies. At least two distinct keloid phenotypes exist, the superficial-spreading/flat keloids and the bulging/raised keloids. Within keloids, the periphery is often seen as the actively growing margin compared to the more quiescent center, although the opposite has also been reported. Interestingly, the normal skin directly surrounding keloids also shows partial keloid characteristics. Keloids are most likely to occur after an inciting stimulus such as (minor and disproportionate) dermal injury or an inflammatory process (environmental factors) at a keloid-prone anatomical site (topological factors) in a genetically predisposed individual (patient-related factors). The specific cellular abnormalities these various patient, topological and environmental factors generate to ultimately result in keloid scar formation are discussed. Existing keloid models can largely be divided into *in vivo* and *in vitro* systems including a number of subdivisions: human/animal, explant/culture, homotypic/heterotypic culture, direct/indirect co-culture, and 3D/monolayer culture. As skin physiology, immunology and wound healing is markedly different in animals and since keloids are exclusive to humans, there is a need for relevant human *in vitro* models. Of these, the direct co-culture systems that generate full thickness keloid equivalents appear the most promising and will be key to further advance keloid research on its pathogenesis and thereby ultimately advance keloid treatment. Finally, the recent change in keloid nomenclature will be discussed, which has moved away from identifying keloids solely as abnormal scars with a purely cosmetic association toward understanding keloids for the fibroproliferative disorder that they are.

## Introduction

As early as approximately 3000 B.C., the existence of keloid scars has been acknowledged in the description of a “*swelling on his breast, large, spreading, and hard*,” which felt like “*touching a ball of wrappings*” in the Edwin Smith Papyrus, the first known surgical treatise describing ancient Egyptian medical practice (Breasted, 1930; Berstein and Roenigk, 1996). Keloids are not mentioned in modern day literature until the early 19th century when Jean Louis Alibert, the father of French Dermatology, first described tumor-like scars which he initially referred to as ‘*les cancroïdes de la peau.*’ When it became clear these cicatricial tumors were in fact non-cancerous, Alibert changed the name to ‘*cheloïde*’ or ‘*keloïde*’ in reference to the Greek word ‘χηλi’ (khçlçé) for crab’s claw and the suffix -oid meaning ‘like.’ Together this is meant to reflect not only the claw-like extension of the keloids but also refers to their horizontal invasive growth beyond the initial wound margins into the surrounding skin (Alibert, 1825; Delpech, 1881; Berstein and Roenigk, 1996).

The keloid incidence rate varies greatly and is known to be influenced by racial ethnicity. The risk of keloid development significantly increases with increasing pigmentation (Burd and Huang, 2005; Wolfram et al., 2009). In the Black and the Hispanic general population, the incidence varies from 4.5–6.2 to 16% (Cosman et al., 1961; Oluwasanmi, 1974; Rockwell et al., 1998); while the incidence in the Taiwanese Chinese and Caucasians is reported to be as low as <1% (Bloom, 1956; Seifert and Mrowietz, 2009; Sun et al., 2014). However, these numbers are largely based on studies from several decades ago with the oldest dating back to 1931. To our knowledge there are no new incidence numbers of keloid scarring in the general population. More recent data is available for very specific subpopulations: in head and neck surgical patients (Young et al., 2014) as well as women after caesarian section (Tulandi et al., 2011), the incidence of keloid scar formation was significantly increased in African Americans (0.8 and 7.1%, respectively) compared with the Caucasian (0.1 and 0.5%, respectively) and Asian or other (0.2 and 5.2%, respectively) population. Interestingly, in Africans with albinism the prevalence rate of 7.5% was not statistically different from the overall prevalence rate of 8.3% in the general population or the 8.5% observed in the normally pigmented African population (Kiprono et al., 2015). It would therefore seem that increased pigmentation in and of itself cannot solely explain the reported ethnic differences in incidence rates (Bran et al., 2009).

In addition to the obvious cosmetic disfigurement, keloids can also produce symptoms of itching and pain (Lee S. S. et al., 2004; Bijlard et al., 2017). A study comparing the quality of life in patients with keloids to that of psoriasis patients found that patients with abnormal scars demonstrated the same reduced quality of life levels as psoriasis patients when compared with healthy controls (Balci et al., 2009). Similarly, a cross-sectional survey on the burden of keloid disease (Bijlard et al., 2017) showed that having keloids was associated with considerable impairment of emotional wellbeing. In summary, keloids may affect a very specific demographic for reasons we do not yet know, but for those affected, these abnormal scars can have significant consequences beyond cosmetics.

The mechanisms behind keloid scarring in particular are still poorly understood (Slemp and Kirschner, 2006; Robles and Berg, 2007; Seifert and Mrowietz, 2009), and this is reflected in our inability to satisfactorily manage this abnormal scar (Niessen et al., 1999; Butler et al., 2008b). Known for its therapy-resistant nature, excision alone has recurrence rates of 55–100% and can even result in the development of a worse scar than before (Robles and Berg, 2007; Butler et al., 2008b; Balci et al., 2009; Shih et al., 2010). To further advance research on the pathogenesis underlying keloid scar formation, there is an urgent need for relevant, true-to-life keloid scar models that resemble the *in vivo* keloid phenotype accurately. This review on keloid scars will discuss histopathological characteristics, inter- and intralesional heterogeneity, the pathogenetic mechanisms, as well as existing scar model systems of keloids.

## Keloid Histopathology

Keloids are primarily a clinical diagnosis (Gulamhuseinwala et al., 2008), and as such are not usually sent in for further analysis by the pathologist. Although the histopathological definition of a keloid scar was not further detailed in the article, Gulamhuseinwala et al. (2008) found that retrospective analysis of H&E stainings of 568 clinically diagnosed keloids only proved accurate in 81% of the cases. Experienced plastic surgeons diagnosed keloids based on the following clinical criteria: the presence of a scar with a history of antecedent local trauma and growth extending beyond its boundary. The non-keloid diagnoses included acne keloidalis (11%), hypertrophic (6%), and even normotrophic (2%) scars and a single pilonidal abscess. Importantly though, no malignancies or dysplasias were reported. Based on these findings, the authors suggested that sending excised keloid tissue for histopathological examination is not necessary if the clinician is an expert and there is a strong clinical suspicion (Gulamhuseinwala et al., 2008). In response to this study, however, Wong and Ogawa pointed out that many clinicians would not be comfortable with the incorrect diagnosis rate of 19% and therefore advocate for post-surgical histopathological confirmation (Wong and Lee, 2008; Ogawa et al., 2009).

The histopathological abnormalities of the full scarring spectrum and normal skin have been summarized in Supplementary Table S1, specific cellular abnormalities in keloid scars are summarized in Supplementary Table S3 and will be elaborated upon in the section ‘Keloid cellular abnormalities’. The histopathological findings on keloid scars will be briefly summarized in this section. The epidermal thickness in keloid scars has been described as anything from atrophic (Koonin, 1964; Bakry et al., 2014) and normal (Moshref and Mufti, 2009; Huang et al., 2014), to sometimes (Ehrlich et al., 1994; Materazzi et al., 2007) or always increased (Bertheim and Hellström, 1994; Chua et al., 2011; Syed et al., 2011; Sidgwick et al., 2013; Jumper et al., 2015; Suttho et al., 2017; Shang et al., 2018). However, the overwhelming majority supports the observation of increased epidermal thickness in keloid scars, and what is more, this was confirmed when thickness was measured in μm (Hellström et al., 2014) as well as number of viable cell layers (Limandjaja et al., 2017, 2019). Similarly, conflicting findings have been reported with regards to rete ridge formation. Reports range from normal rete ridge formation (Lee J. Y. Y. et al., 2004; Moshref and Mufti, 2009) to reduced (Koonin, 1964; Chong et al., 2015; Jumper et al., 2015; Suttho et al., 2017; Shang et al., 2018) or complete absence thereof (Ehrlich et al., 1994; Meenakshi et al., 2005; Huang et al., 2014), although none have attempted to objectively measure the extent of rete ridge formation. Overall, most studies, including our own histopathological studies (Limandjaja et al., 2017, 2019), appear to support the findings of a flattened epidermis with increased thickness. Epidermal differentiation appears mostly unaffected (Bloor et al., 2003; Ong et al., 2010; Limandjaja et al., 2017, 2019). Although increased epidermal activation/proliferation has been observed (Prathiba et al., 2001; Bloor et al., 2003; Ong et al., 2010), this is in contrast with the findings from our extensive immunohistochemical analysis of the keloid epidermis (Limandjaja et al., 2017). We showed normal levels of epidermal proliferation and differentiation in the thickened keloid epidermis, save for the precocious expression of terminal differentiation marker involucrin. We therefore proposed that increased epidermal thickness was not the result of epidermal hyperproliferation, but was associated with abnormal differentiation instead. Interestingly, keloid keratinocytes also showed increased expression of epithelial-mesenchymal transition (EMT) markers (Chua et al., 2011; Ma et al., 2015; Yan et al., 2015; Hahn et al., 2016; Kuwahara et al., 2016).

Histopathological studies of the keloid dermis showed that fibroblasts were present in higher numbers (Ueda et al., 1999; Tanaka et al., 2004; Meenakshi et al., 2009; Jiao et al., 2017). Other dermal cell types residing in keloids include myofibroblasts (Santucci et al., 2001; Kamath et al., 2002; Lee J. Y. Y. et al., 2004; Lee et al., 2012; Moshref and Mufti, 2009; Shin et al., 2016) and fibrocytes (Iqbal et al., 2012; Shin et al., 2016), both were present in increased numbers. In our whole biopsy image analysis of keloid tissue, CD34 expression was found to be absent from the keloid dermis, but constitutively and abundantly present in normal skin and normotrophic scars (Limandjaja et al., 2019). Interestingly, within these CD34− dermal regions, we found senescent (p16+) mesenchymal cells (vimentin+) as well as myofibroblasts (α-SMA+).

Overall trends in the dermal ECM composition include increased levels of collagen I and III (Naitoh et al., 2001; Syed et al., 2011) with increased collagen bundle thickness (Verhaegen et al., 2009); increased fibronectin (Kischer and Hendrix, 1983), glycosaminoglycans (Carrino et al., 2012), chondroitin sulfate (Ikeda et al., 2009), biglycan (Hunzelmann et al., 1996), versican (Carrino et al., 2012), tenascin (Dalkowski et al., 1999), and periostin (Zhou et al., 2010; Maeda et al., 2019); while levels of elastin (Szulgit et al., 2002; Ikeda et al., 2009; Theoret et al., 2013), and decorin (Carrino et al., 2012) were reduced. Dermal hyaluronic acid expression showed variable results (Alaish et al., 1995; Meyer et al., 2000; Ikeda et al., 2009; Yagi et al., 2013), but unlike normal skin, its expression was equal in both the keloid epidermis and dermis (Tan et al., 2011). Reports on vascularity are also highly variable, both increased (Ehrlich et al., 1994; Amadeu et al., 2003; Tanaka et al., 2004; Materazzi et al., 2007; Ong et al., 2007b; Syed and Bayat, 2012; Bakry et al., 2014) and decreased vascular density (Beer et al., 1998; Ueda et al., 2004; Kurokawa et al., 2010; Theoret et al., 2013) has been observed in keloids. Nerve fiber density appears to be increased in keloids compared with normal skin (Hochman et al., 2008; Drummond et al., 2017). Lastly, keloids also show increased immune cell infiltration (Amadeu et al., 2003; Sharquie and Al-Dhalimi, 2003; Tanaka et al., 2004; Shaker et al., 2011; Jiao et al., 2015; Luo et al., 2017), with higher quantities of macrophages (Boyce et al., 2001; Shaker et al., 2011; Jiao et al., 2015) and T-lymphocytes (Shaker et al., 2011; Murao et al., 2014; Jiao et al., 2015) in particular. An extensive review by Jumper et al. (2015) on the histopathology of keloid scars has reiterated most of the aforementioned findings, and further emphasizes the following as most frequently occurring and therefore most discerning features of keloid scars: a thickened, flattened epidermis; a tongue-like advancing edge in the dermis; haphazard, thick, hyalinized collagen bundles as the predominant dermal feature, with subsequent loss of the papillary-reticular boundary; increased dermal cellularity; signs of inflammation; and variable α-SMA expression.

Unlike their keloid counterparts, hypertrophic scars are raised scars whose growth remains within the borders of the original wound (Burd and Huang, 2005) and they can be difficult to distinguish histopathologically. Keloidal collagen (Cosman et al., 1961; Santucci et al., 2001; Ogawa et al., 2009) vs. α-SMA and dermal nodules (Ehrlich et al., 1994; Huang et al., 2014) have often been cited as pathognomonic features for keloids or hypertrophic scars, respectively, but conflicting reports abound (Muir, 1990; Ehrlich et al., 1994; Santucci et al., 2001; Lee J. Y. Y. et al., 2004; Ogawa et al., 2009; Ali et al., 2010; Bux and Madaree, 2010; Huang et al., 2014). In a histopathological study (Limandjaja et al., 2019) comparing both scar types, α-SMA and dermal nodules were present in both scars and while keloidal collagen remained a strong keloid marker, it was also observed in one of the hypertrophic scars. Additionally, a thickened epidermis with involucrin overexpression and a CD34−/α-SMA+/p16+ dermal cell population could be found in both scar types, although α-SMA and p16 immunoreactivity were present in higher degrees in hypertrophic scars vs. keloids, respectively. In short, despite the clearly defined clinical distinction between the two abnormal scar types, the histological distinction between hypertrophic and keloid scars remains a source of contention, especially in the early stages (Burd and Huang, 2005).

## Inter- and Intralesional Keloid Heterogeneity

### Interlesional Heterogeneity

**FIGURE 1:**
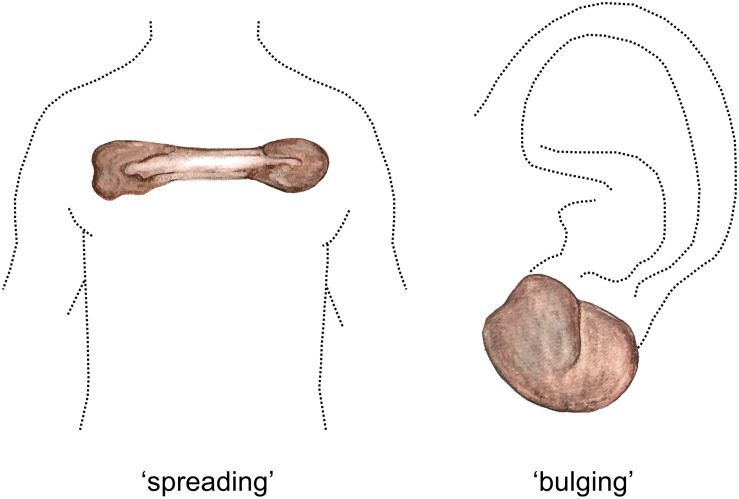
Spreading vs. bulging keloid phenotypes. Watercolor illustration. Left figure shows a typical keloid of the ‘spreading’ phenotype located on the anterior chest, with quiescent center and an actively growing peripheral margin. Right figure shows a keloid of the ‘bulging’ phenotype, which are bulbous in shape and can often be observed on the earlobe. Figure first published in Limandjaja (2019), used with permission.

Several reports suggest that distinct keloid phenotypes may exist. As early as 1960, Conway et al. (1960) discerned between nodular raised keloids and flat keloids often observed on the sternum. More recently, Bella et al. (2011) studied large multigenerational pedigrees of patients with familial keloids in three rural African tribes. The superficial spreading phenotype predominated in two of the tribes, while the raised vertical phenotype predominated in the remaining tribe (Figure 1). Superficial spreading keloids show irregular subepidermal spread with irregular areas of hyper- and hypopigmentation, they are mostly raised at the edges and are characterized by a central flattened and quiescent area. This central area is often regularly or hypopigmented, while the margins show hyperpigmentation. In contrast, raised keloid scars are prominently bulbous in shape with distinct borders and may have limited areas of central quiescence. Another form of interlesional keloid heterogeneity was proposed by Akaishi et al. (2010), who distinguished between regular keloids with a round shape and clear curving lines, and irregular keloids with irregular shapes and lines. The authors found that the irregularly shaped keloids showed a significant increase in infection and previous surgery rates, and proposed that in contrast, the shape of regular keloids was determined by skin tension alone. In predilection body sites exposed to constant stretching (e.g., scapula, chest, and shoulder), the butterfly, the crab’s claw or the dumbbell have also been described as typical keloid shapes which are predominantly determined by local mechanical factors (Huang et al., 2017). In short, several distinct keloid phenotypes have been described, the division of which appears to be based predominantly on the keloid’s growth pattern and/or the resulting shape.

### Intralesional Heterogeneity

It is also likely that heterogeneity exists within keloid scars (see Supplementary Table S1). Based on clinical observations, the most often described distinction is that of a red, raised peripheral margin which actively invades the surrounding skin and more depressed, lighter colored center showing clinical regression (Louw et al., 1997; Lu et al., 2007a; Seifert et al., 2008). This peripheral-central distinction matches the description of the superficial spreading keloid phenotype (Bella et al., 2011). Symptoms of strong itching (Lee S. S. et al., 2004) predominate in the more pigmented keloid margin (Louw et al., 1997; Le et al., 2004; Lu et al., 2007a; Bella et al., 2011), together with hypercellularity (Appleton et al., 1996; Ladin et al., 1998; Akasaka et al., 2001; Varmeh et al., 2011; Huang et al., 2014), increased vascularity (Appleton et al., 1996; Le et al., 2004; Touchi et al., 2016) and immune cell infiltration (Appleton et al., 1996; Le et al., 2004). Reduced apoptosis (Lu et al., 2007a; Seifert et al., 2008), increased proliferation (Varmeh et al., 2011; Suttho et al., 2017) and increased cellular activity (Louw et al., 1997), all contribute to enlarging the pool of ECM-producing fibroblasts in this region and support the hypothesized increased keloid activity (see Supplementary Table S2A). In contrast, the central keloid region shows either hypopigmentation or regular pigmentation (Louw et al., 1997; Le et al., 2004; Lu et al., 2007a; Bella et al., 2011) with pain as the main symptom (Lee S. S. et al., 2004), as well as hypocellularity (Appleton et al., 1996; Ladin et al., 1998; Akasaka et al., 2001; Varmeh et al., 2011; Huang et al., 2014) and reduced vascularity (Appleton et al., 1996; Le et al., 2004; Touchi et al., 2016) (see Supplementary Table S2A). Fibroblasts derived from this central region generally show signs of inactivity (Louw et al., 1997), as well as reduced proliferation (Varmeh et al., 2011; Suttho et al., 2017), increased apoptosis (Lu et al., 2007a; Seifert et al., 2008) and senescence (Varmeh et al., 2011). Taken together with the increased expression of ECM-degrading genes (Seifert et al., 2008), the central region appears to be the area of relative quiescence.

Overall, the majority of studies support the concept of an actively developing periphery and a more quiescent central region, but the reverse has also been postulated with an active role for the central region rather than the periphery. The keloid center has been reported to show increased proliferation (Giugliano et al., 2003; Tsujita-Kyutoku et al., 2005), the absence of apoptosis (Appleton et al., 1996; Sayah et al., 1999; Akasaka et al., 2001), increased expression of fibrosis-associated genes (e.g., TGFβRI, SMAD 2, and SMAD3) (Tsujita-Kyutoku et al., 2005) and certain wound healing mediators (IL-6 and VEGF) (Giugliano et al., 2003), all of which support a more pro-active role for this region. In line with these findings, our *in vitro* reconstructed (Limandjaja et al., 2018b) different keloid regions showed that differences existed between the regions in terms of scar parameter expression. The central deep keloid region showed the more exaggerated keloid phenotype with respect to increased contraction, increased epidermal thickness, reduced HGF secretion and reduced collagen type IV α2 chain dermal gene expression.

Comparison of the keloid heterogeneity findings remains difficult due to the varying definitions of what constitutes the periphery and the center within a keloid, as this may differ significantly between studies. Additionally, we suspected that the apparent dichotomy between results supporting an active keloid periphery and those whose findings ascribe this active role to the keloid center, may be explained by the existence of different keloid phenotypes (Limandjaja et al., 2018b). Various keloid phenotypes have been described in the previous paragraph, but we would like to propose an even simpler classification based on the work of Bella et al. (2011) and Supp et al. (2012b): namely that of a more concave ‘superficial spreading’ and raised or ‘bulging’ keloids (see Figure 1) (Bella et al., 2011). Depending on the phenotype, the actively expanding region could be located in the periphery or the deeper central region (Supp et al., 2012b). As all the keloids included in the study were of the ‘bulging’ phenotype, it follows that the central deep region would show the most aggressive keloid phenotype.

Furthermore, heterogeneity has also been observed in regions other than the periphery and the center, these are summarized in Supplementary Table S2B. An often-used division is that between different dermal layers (Russell et al., 1989, 1995; Luo et al., 2001; Supp et al., 2012b; Chong et al., 2015, 2018; Jiao et al., 2017), in which case the middle or deepest dermal layers were usually found to act more aggressively compared with the more superficial layers. The conflicting reports on the peripheral and central keloid regions notwithstanding, it is clear that differences exist between different lesional sites within a keloid and these abnormal scars should therefore not simply be considered or treated as a homogenous growth. Because of this, we strongly advocate for the inclusion of a description of the keloid phenotype/shape and the use of schematic drawings to indicate from where within a keloid samples were taken for experimentation.

### Normal Skin Surrounding Keloids

The normal skin directly adjacent to and surrounding keloid scars is also rarely included in keloid research. Although similarity to normal skin has been reported (Jumper et al., 2017), the majority of reported results suggest that the surrounding normal skin behaves more like keloid tissue than normal skin. For example, the surrounding normal skin often itches like the keloid periphery (Lee S. S. et al., 2004) and shows increased blood flow compared with unaffected normal skin (Liu et al., 2016). On a cellular level, increased staining of the hematopoietic stem cell marker c-KIT (Bakry et al., 2014) and heat shock protein 70 (Lee et al., 2013) has been observed in both keloids and their surrounding normal skin, while keratinocytes and fibroblasts from the surrounding normal skin shared the abnormal expression of many of the same genes in keloid-derived keratinocytes and fibroblasts (Hahn et al., 2013). In the dermal compartment, the epidermal appendages lost from keloid tissue reappear in the surrounding normal skin in reduced capacity; and the dense and excessive collagen deposition of the keloid can extend into the ECM of the surrounding normal skin, which is otherwise loosely organized, with thin, wavy or even fragmented collagen fibers (Lee et al., 2013; Lee W. J. et al., 2015; Bakry et al., 2014; Jiao et al., 2017). Similarly, portions of nodules from the adjacent keloid have also been found to extend into the surrounding normal skin (Kischer and Pindur, 1990). The dermis adjacent to keloids is more cellular but less crowded compared with healthy skin, and shows significant lymphocyte infiltration (Bakry et al., 2014; Lee W. J. et al., 2015; Jiao et al., 2017). The skin surrounding keloids also differed from unaffected healthy skin with respect to proliferation and apoptosis. Increased dermal proliferation and increased numbers of apoptotic keratinocytes (Appleton et al., 1996) were only observed in the surrounding normal skin, and absent from healthy skin. Lastly, Dohi et al. (2019) proposed an even more prominent role for surrounding normal skin as the driving force for keloid progression into the normal skin, via local preferential increased mechanical strain. Conversely, the surrounding normal skin has also been reported to differ from keloids. Compared with keloid scars, the surrounding normal skin has more blood vessels (Beer et al., 1998) and showed increased expression of the proliferative PCNA marker in the dermis, which was normally absent in both normal skin and keloids (Appleton et al., 1996). The surrounding skin also showed strong, increased levels of CD34 staining in contrast with the CD34 absence in abnormal scars (Erdag et al., 2008). It also lacks the abnormal thermosensory thresholds to warmth, cold, heat and pain sensations reported for keloids (Lee S. S. et al., 2004). All things considered, current literature suggests that the surrounding normal skin shares several features with the adjacent keloid and is therefore a relevant area to include for further investigation.

In our histopathological analysis of keloids, we found that the normal skin directly adjacent to the keloids mostly resembled normal skin or mature normotrophic scar (Limandjaja et al., 2019). An epidermis of normal thickness and rete ridge formation with normal differentiation and proliferation could be seen, together with a CD34+/α-SMA−/p16− phenotype instead of the CD34−/α-SMA+/p16+ phenotype associated with keloid scars. However, the most interesting findings emerged from our *in vitro* work (Limandjaja et al., 2018b), where changes were observed which in and of itself were not statistically significant but overall formed a clear pattern of intermediate abnormal expression. Across all the abnormal scar parameters present in the *in vitro* keloid scar model, the *in vitro* reconstructed surrounding normal skin showed a phenotype more extreme than true normal skin but less aggressive than the peripheral keloid models. With respect to contraction, α-SMA immunoreactivity, HGF secretion and collagen type IV α2 gene expression, the surrounding normal skin showed intermediate between truly unaffected normal skin and keloid scar.

Taken together, we and others have shown that heterogeneity exists within keloid scars. For future studies it would therefore be imperative to mention the shape and growth pattern of the keloid (superficial spreading or bulging) and additionally, it should always be mentioned where in the keloid any tissue for experimentation was obtained from, preferably with a schematic overview for unambiguous clarification. Additionally, we would argue that it is worth including the surrounding normal skin in any keloid research study when possible.

## Keloid Scar Pathogenesis: Where Do We Stand?

**FIGURE 2:**
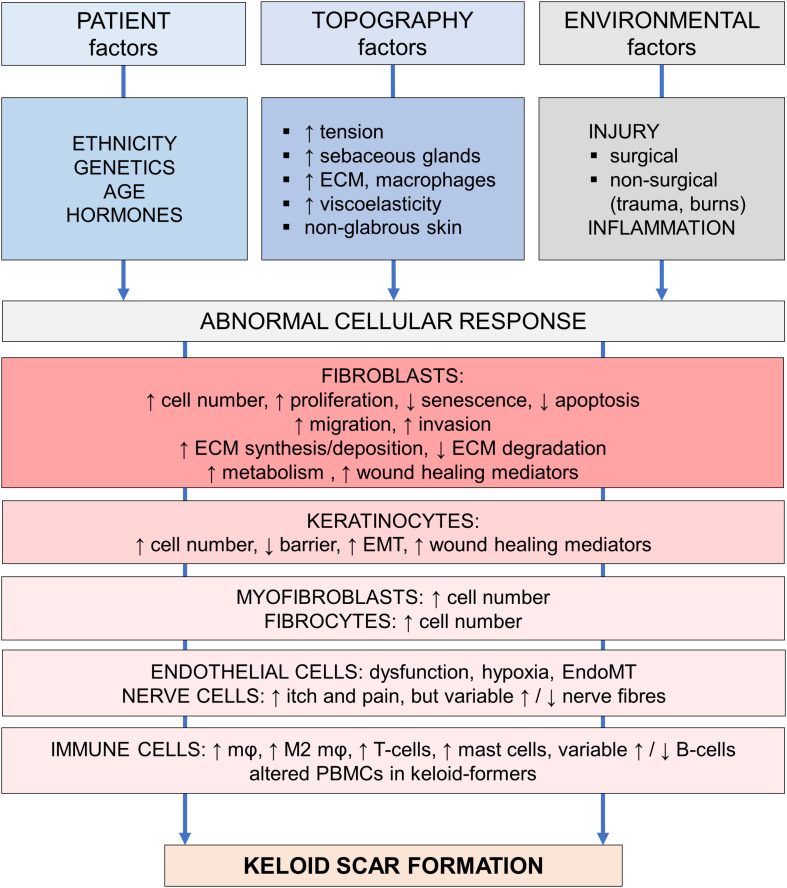
Pathogenesis of keloid scarring. Overview of the various factors involved in keloid pathogenesis, adapted from Wolfram et al. (2009). This figure is meant to provide a provisionary framework to help organize the multitude of pathogenetic findings in a logical and systematic way. Abbreviations; ECM, extracellular matrix; EMT, epithelial-mesenchymal transition; EndoMT, endothelial-mesenchymal transition; mφ, macrophage; M2, alternatively activated, pro-fibrotic macrophage subtype; PBMCs, peripheral blood mononuclear cells. Figure first published in Limandjaja (2019), used with permission.

The development of a human *in vitro* keloid scar model resembling *in vivo* keloid tissue would not only benefit drug development and testing, it would also greatly aid research into the underlying mechanisms leading to keloid scarring. The fact that keloid pathogenesis remains so poorly understood (Slemp and Kirschner, 2006; Wolfram et al., 2009), has been the bane of affected patients and clinicians alike. Despite the many theories proposed by experts, no single unifying hypothesis has been put forward (Seifert and Mrowietz, 2009). In Figure 2, adapted from Wolfram et al. (2009), the various abnormalities reported in keloid tissue and keloid-derived cells are organized in several tiers: patient, topography or special skin sites, and environmental factors all contribute to the expression of abnormal cellular responses, which eventually lead to keloid scar formation. In the following sections, recent and relevant findings for each of these factors will be discussed.

### Patient-Related Factors Influencing Keloid Scarring

Ethnicity, genetic predisposition, gender and age are patient characteristics which may influence keloid predilection (Wolfram et al., 2009). There is no conclusive evidence in favor of differences in occurrence based on gender. Some have reported that keloids are more likely to occur in women than men (Bayat et al., 2005; Burd and Huang, 2005; Seifert and Mrowietz, 2009; Middelkoop et al., 2011; Young et al., 2014), but this may also reflect at least in part, the overall greater awareness of unaesthetic scarring in women and a consequent higher tendency to seek medical assistance (Marneros et al., 2001; Burd, 2006). Young age is also associated with increased risk of abnormal scarring (Middelkoop et al., 2011). Keloids can develop at any age, but incidence is highest between the ages of 10–30 years (Bayat et al., 2005; Seifert and Mrowietz, 2009; Young et al., 2014; Lu et al., 2015). Because of the peak in incidence immediately post-puberty, exacerbations during pregnancy and resolution after menopause, a potential role for endocrinological hyperactivity in keloid pathogenesis has also been proposed (Rockwell et al., 1998; Seifert and Mrowietz, 2009; Huang and Ogawa, 2013a; Glass, 2017).

Perhaps the most relevant patient-related factor is the genetic predisposition for keloid formation in certain individuals. Ethnic differences in prevalence showed that darker pigmented individuals are at higher risk of developing keloid scars (Marneros et al., 2001; Al-Attar et al., 2006). Additionally, having a family member with keloids is associated with increased keloid prevalence (Bayat et al., 2005; Kiprono et al., 2015; Lu et al., 2015). This is predominantly the case for first degree relatives, as demonstrated by a heritability of 72, 41, and 17% for first, second, and third degree relatives in the Chinese population (Lu et al., 2015). Individuals with a family history of keloids are also at higher risk of developing multiple keloids and developing keloids of greater severity (Bayat et al., 2005; Lu et al., 2015). The familial heritability, the increased prevalence in certain ethnicities and common occurrence in twins, all strongly support the concept of genetic susceptibility in patients with keloid scars (Marneros et al., 2001; Shih and Bayat, 2010; Glass, 2017). Other lines of evidence pointing to a genetic influence on keloid predisposition include familial inheritance patterns, linkage studies, case-control association studies, and gene expression studies (Shih and Bayat, 2010; Glass, 2017). Different modes of inheritance have been reported, varying from autosomal recessive and X-linked to autosomal dominant (Shih and Bayat, 2010; Glass, 2017). Shih and Bayat (2010) have previously reviewed the available evidence and suggest that most evidence points to an autosomal dominant inheritance pattern with incomplete penetration and variable expression, this then also explains why carriers do not always express the keloid phenotype and why keloid patients do not always respond to trauma with keloid scarring. Despite their valuable contribution to our understanding of genetic predilection for keloids, familial inheritance studies have not led to the discovery of any particular predisposing genes (Glass, 2017). Multiple gene mapping methods as well as targeted gene pathway investigations have identified several gene polymorphisms (*NEDD4, FOXL2, MYO1E*, and *MYO7A* also *HLA*) associated with keloids (Nakashima et al., 2010; Zhu et al., 2013; Velez Edwards et al., 2014; Glass, 2017), but the underlying mechanism is still unclear. Similarly, various abnormalities in gene expression have shown highly variable results between studies (Shih and Bayat, 2010), but affected genes are known to be involved in the ECM, inflammation and apoptosis (Shih and Bayat, 2010). Aside from these inherited gene mutations, acquired altered gene expression in the form of epigenetic modification may also play a role in keloid pathogenesis and further complicates matters (Glass, 2017; He et al., 2017). Ultimately, however, the specific genetic variation responsible for keloid scarring has yet to be identified, but likely involves more than a single gene. Additionally, different keloid patients probably carry different gene polymorphisms which can all lead to keloid scar formation, this would explain the variations in keloid phenotype observed in different people (Shih and Bayat, 2010; Velez Edwards et al., 2014; Glass, 2017).

### Topography-Related Factors Influencing Keloid Scarring

It is important to note that patients with a history of keloid scarring do not necessarily form keloids after every injury (Slemp and Kirschner, 2006), two identical incisions can generate one normal scar and one keloid in the same individual (Fong et al., 1999; Fong and Bay, 2002; Al-Attar et al., 2006). Certain body sites are more prone to keloid scarring, thus the location of the wound influences risk of keloid scar formation (Wolfram et al., 2009; Middelkoop et al., 2011). The earlobe, neck, sternum, upper back, shoulders and upper limbs all constitute keloid-prone anatomical sites (Murray et al., 1981; Bayat et al., 2005; Burd and Huang, 2005; Bella et al., 2011; Middelkoop et al., 2011).

Although the keloid-prone earlobe and anterior chest have been described as tension-free areas (Brody, 1990; Ogawa et al., 2003; Al-Attar et al., 2006), the most popular explanation for why keloids occur more frequently at certain body sites remains the theory that these are regions of increased skin tension that are subject to constant stretching during normal movement (Peacock et al., 1970; Rockwell et al., 1998; Butler et al., 2008a; Bux and Madaree, 2012; Ogawa et al., 2012). There is no consensus on whether elasticity may explain the differences between keloid-prone and keloid-protected sites. Bux and Madaree (2012) reported that keloid-prone sites are characterized by high tension with low stretch and low elastic modulus. In contrast, Sano et al. (2018) observed that with exception of the earlobes, sites less prone to pathologic scarring (e.g., palmoplantar regions) were “*comparatively hard*,” characterized by low distensibility and reduced elasticity. In contrast, keloid susceptible sites showed high distensibility and increased elasticity. Additionally, high sebaceous gland density (Yagi et al., 1979; Fong et al., 1999; Fong and Bay, 2002; Al-Attar et al., 2006), increased collagen and decreased M1 macrophage numbers (Butzelaar et al., 2017), are all characteristics of keloid-prone skin which may promote keloid scar formation in genetically predisposed individuals.

### Environmental Factors Influencing Keloid Scarring

Although spontaneous keloid scar formation has been reported (Jfri et al., 2015), it is a rare occurrence that has been reported in association with certain syndromes such as Rubenstein-Taybi and Goeminne syndrome (Jfri et al., 2015), or may simply reflect a laps in memory (Robles and Berg, 2007; Jfri et al., 2015). Environmental factors are therefore generally an essential prerequisite to keloid scar formation as some form of assault to the skin has to take place to incite keloidogenesis (Bran et al., 2009; Wolfram et al., 2009; Shih and Bayat, 2010). These keloid-inducing events vary from minor to major antecedent trauma, as well as any process resulting in skin inflammation. Insect bites or vaccinations are examples of minor insults to the skin which may be so minor as to not be remembered by the patient at all, while major trauma is usually observed in the setting of surgical and non-surgical wound healing. Examples of the latter group include lacerations, abrasions, piercings, tattooing or blunt trauma. Additionally, inflammatory skin conditions such as acne, (peri)folliculitis, chicken pox, herpes zoster and hidradenitis suppurativa, may also lead to the development of keloids (Nemeth, 1993; Murray, 1994; English and Shenefelt, 1999; Bran et al., 2009). Isotretinoin is often used to treat acne and has been suggested to act as an additional predisposing factor, though this has not yet been proven conclusively (Guadanhim et al., 2016). While burns have often been mentioned as one of the many potential keloid-inducing events (Trusler and Bauer, 1948; Nemeth, 1993; Murray, 1994; English and Shenefelt, 1999; Bran et al., 2009), they are usually associated with the formation of widespread hypertrophic scars (Middelkoop et al., 2011; Gold et al., 2014) rather than keloids. Fortunately, venipuncture has not been reported to induce keloid scarring (Yadav et al., 1995; Robles and Berg, 2007). Regardless of the type of injury however, the resultant keloid scar response is characteristically disproportionate to the original inciting injury (Tuan and Nichter, 1998).

To summarize, keloid formation is most likely to occur after an inciting stimulus such as dermal injury or inflammatory process (environmental factor) at a keloid-prone anatomical site (topological factor) in a genetically predisposed individual (patient-related factor). The specific cellular abnormalities this generates to ultimately result in keloid scar formation, are discussed next (Figure 2).

### Keloid Cellular Abnormalities

Although the keloid fibroblast is still considered the main culprit responsible for keloid scar formation, recent studies have shifted the focus to recognize the potential role of the epidermal compartment and the immune system in keloidogenesis. This section will address the trends in reported cellular abnormalities across the entire spectrum of cells present in skin and/or involved in wound healing (see Supplementary Tables S3A–D).

#### Abnormalities in Keloid Epidermal Cell Population

Often overlooked or even designated as ‘normal appearing’ (Butler et al., 2008b; Hollywood et al., 2010), the keloid epidermis has only recently started garnering attention in keloid research. A summary of the reported keloid epidermal abnormalities has been listed in Supplementary Table S3A and a summary of the histopathological epidermal abnormalities can be found in the paragraph ‘Keloid Histopathology’ and in Supplementary Table S1. The abnormalities of the keloid epidermis are not limited to those visible by histopathology alone, there is growing evidence that its barrier function is also affected as measurements of transepidermal water loss (TEWL) and high-frequency conductance suggest keloid scars may show altered stratum corneum function compared with healthy skin (Suetake et al., 1996; Sogabe et al., 2002). In line with these findings, we found that the specific abnormal overexpression of terminal differentiation marker involucrin was not only associated with increased epidermal thickness, but also with disorganization of the stratum corneum as visualized by transmission electron microscopy (Limandjaja et al., 2017). The following paragraphs will focus on abnormalities reported for keloid keratinocytes and melanocytes. Langerhans cells also reside in the epidermal compartment, but will be discussed in the paragraph on keloid immune cells.

##### Keloid keratinocytes

Keloid keratinocytes may have a more direct role in keloid scarring than previously assumed. Increased expression of growth factors and cytokines such as CTGF (Khoo et al., 2006), HGF and its receptor c-Met (Mukhopadhyay et al., 2010), VEGF and PLGF (Ong et al., 2007b) have been demonstrated in keloid-derived keratinocytes. Furthermore, cultured keloid keratinocytes were found to differentially express 538 genes in a study by Li and Wu (2016) and of these, further functional analysis identified homeobox A7 (*HOXA7*), minichromosome maintenance 8 (*MCM8*), proteasome subunit α type 4 (*PSMA4*) and proteasome subunit β type 2 (*PSMB2*) as key differentially expressed genes. In another gene expression study, Hahn et al. (2013) found abnormal expression of genes involved in differentiation, cell–cell adhesion and increased motility. Keloid keratinocytes also contribute to keloid scarring by paracrine regulation of ECM synthesis in fibroblasts, as evidenced by their ability to induce a more profibrotic phenotype *in vitro* even in fibroblasts of normal skin origin (Lim et al., 2002).

Lastly, several studies support a role for epithelial-mesenchymal transition (EMT) in keloid scarring, a phenomenon by which epithelial cells undergo phenotypic changes and acquire more mesenchymal characteristics (Stone et al., 2017). EMT has been found to occur in wound healing, and plays a role in fibrosis by serving as a source of myofibroblast generation (Stone et al., 2017). The changes associated with EMT have been reported in keloid scars and involve the loss of epithelial cell markers such as E-cadherin (Ma et al., 2015; Yan et al., 2015; Hahn et al., 2016) and gain of mesenchymal characteristics such as vimentin and FSP-1 (fibroblast specific protein 1) expression (Yan et al., 2010, 2015; Ma et al., 2015; Hahn et al., 2016; Kuwahara et al., 2016), combined with changes in cell shape toward a more motile and migratory phenotype (Hahn et al., 2013, 2016; Supp et al., 2014; Stone et al., 2017). In short, the fundamental abnormalities found in the keloid keratinocytes with respect to wound healing mediator secretion, differentially expressed genes, paracrine effects on co-cultured cells and epithelial-mesenchymal transition, all support a more active role for keratinocytes in keloidogenesis.

##### Keloid melanocytes

Little has been published on the role of melanocytes in keloid pathogenesis (see Supplementary Tables S1, S3A), despite the long observed increased keloid incidence in individuals with darker pigmentation (Burd and Huang, 2005; Wolfram et al., 2009). To our knowledge, only Gao et al. (2013) addressed the potential role of melanocytes specifically in both hypertrophic and keloid scar formation and proposed that during wound healing, a damaged basement membrane allows the melanocytes to interact with the dermal fibroblasts. The ensuing increase in fibroblast proliferation and collagen production together with activation of the TGF-β pathway, promote abnormal scar formation. They performed indirect co-culture experiments in which melanocytes were able to induce increased levels of proliferation, collagen I, TGF-β1 and its downstream p-SMAD 2/3 expression in normal fibroblasts compared with monocultured fibroblasts.

The increased melanin in keloid-prone patients may also contribute to keloid scar formation by inhibiting the senescence-inducing and anti-inflammatory effects of UVB radiation (Wirohadidjojo et al., 2011) and vitamin D (Cooke et al., 2005), respectively. However, variations in melanin levels alone cannot fully explain the association of keloids with darker pigmented individuals, as it has been reported that the African albino population shows similar keloid prevalence rates to the normally pigmented Africans (Kiprono et al., 2015). Aberrations have also been reported in the steps involved in melanogenesis, the process by which melanin is generated (Koonin, 1964; Slominski et al., 1993; Song, 2014; Wadhwa et al., 2016). For example, we now know that polymorphisms in the MC1R gene are in fact responsible for ethnic variations in pigmentation (Videira et al., 2013), and it has been demonstrated that this receptor is not only expressed on melanocytes but can also be found on dermal fibroblasts (Stanisz et al., 2011). In fact, Luo et al. (2013) reported that keloid scars and particularly keloid fibroblasts, showed reduced expression of the melanocortin-1 receptor. They proposed that this may negate the α-MSH-mediated suppression of collagen synthesis and myofibroblast formation, thereby stimulating keloid development. Additionally keloid fibroblasts have also been found to be resistant to inhibitory effects of TGF-β1 on POMC expression (Teofoli et al., 1997; Lotti et al., 1999). Thus, keloids association with increased pigmentation may very well not reflect a primary abnormality in the melanocytes, but a concomitant altered function of a shared receptor in the fibroblasts.

#### Abnormalities in Keloid Dermal Cell Population

By their very nature, hypertrophic and keloid scars are defined by the presence of raised, protruding scar tissue. The focus of most studies has therefore understandably been on the dermal component and more specifically on the extracellular matrix (ECM) and the ECM-producing fibroblasts. Both keloid and hypertrophic scars showed increased cellularity and were in excess of all three primary ECM components of water, collagen and proteoglycans. Notably, in keloids these processes were significantly upregulated compared with normal skin and hypertrophic scars (Ueda et al., 1999; Miller et al., 2003; Meenakshi et al., 2005).

A summary of the reported keloid dermal abnormalities has been listed in Supplementary Tables S3B–D, and a summary of the dermal histopathological abnormalities can be found in the paragraph ‘Keloid Histopathology’ and in Supplementary Table S1. The following paragraphs will detail abnormalities reported for keloid fibroblasts and keratinocyte/fibroblast interactions, myofibroblasts, fibrocytes, endothelial cells, and nerve cells.

##### Keloid fibroblasts

An overwhelming number of studies have been devoted to the keloid-derived fibroblast. However, the sheer multitude of papers published on keloid fibroblasts have made it impossible to discuss them all in this review and is outside our scope. For this reason, the focus of this review was limited to fibroblast abnormalities as they pertain to the main themes listed by Marneros and Krieg (2004) and Robles and Berg (2007): proliferation, ECM synthesis and degradation, expression of wound healing mediators and apoptosis. A summary of these publications is listed in Supplementary Table S3B, overall trends in these *in vitro* monoculture findings will be summarized in the following paragraphs.

There is an overall increase in the number of fibroblasts in keloids, most likely mediated by increased proliferation rates. Although some have also reported normal or even decreased proliferation rates in keloid fibroblasts, the overwhelming majority of *in vitro* monolayer studies support keloid fibroblast hyperproliferation (Russell et al., 1988; Concannon et al., 1993; Blume-Peytavi et al., 1997; Carroll et al., 2002; Carroll and Koch, 2003; Giugliano et al., 2003; Hanasono et al., 2003, 2004; Meenakshi et al., 2005; Lim et al., 2006, 2009; Yeh et al., 2006; Ghazizadeh et al., 2007; Ong et al., 2007a; Akino et al., 2008; Witt et al., 2008; Zhang G. et al., 2009; Jing et al., 2010; Romero-Valdovinos et al., 2011; He et al., 2012; Syed and Bayat, 2012; Jurzak and Adamczyk, 2013; Wang et al., 2013; Xin et al., 2017). In conjunction with increased proliferation, reduced apoptosis by any means would also lead to a cumulative net increase in the keloid fibroblast population. Despite some papers reporting increased apoptosis (Akasaka et al., 2000, 2005), overall, the majority of studies report reduced apoptosis (Ladin et al., 1998; Messadi et al., 1999; Chipev et al., 2000; Luo et al., 2001; Funayama et al., 2003; Tucci-Viegas et al., 2010; Wang et al., 2013). Apoptosis is also reduced in keloid fibroblasts by upregulation of apoptosis-resistance (Ohtsuru et al., 2000; Messadi et al., 2004; Lu et al., 2007b; Seifert et al., 2008), as well as telomere dysfunction and defective senescence. Findings of telomerase upregulation (Yu et al., 2016) and consequent telomere lengthening (Granick et al., 2011; Yu et al., 2016) in keloid fibroblasts support lifespan-prolonging effects of telomere dysfunction, although telomere shortening as a result of oxidative stress has also been reported in 30% of the keloids studied by De Felice et al. (2009). In normal wound healing, fibroblasts eventually become senescent and can then act as inhibitors in the regulation of fibroblast proliferation and ECM synthesis. In this way, defective senescence may also result in a net increase in fibroblast density (Blažić and Brajac, 2006), but literature on senescence in keloid fibroblasts has been sparse and even counterintuitive (Varmeh et al., 2011).

In line with their invasive nature, keloid fibroblasts also show increased migration (Fujiwara et al., 2005a; Lim et al., 2006; Witt et al., 2008; Wen et al., 2011; Syed and Bayat, 2012; Wang et al., 2013; Fang et al., 2016; Jumper et al., 2017; Hsu et al., 2018) and capacity for invasion in 3D invasion assays (Dienus et al., 2010; He et al., 2012; Syed and Bayat, 2012; Wang et al., 2018). Furthermore, increased metabolic activity (Meenakshi et al., 2005; Vincent et al., 2008), increased ECM synthesis (McCoy et al., 1982; Abergel et al., 1987; Ala-Kokko et al., 1987; Babu et al., 1989; Berman and Duncan, 1989; Suzawa et al., 1992; Fujiwara et al., 2005a; Ong et al., 2007a; He et al., 2012) and deposition (Abergel et al., 1985; Uzawa et al., 2003; Fang et al., 2016) combined with reduced ECM degradation (Abergel et al., 1985; Berman and Duncan, 1989; Uchida et al., 2003; Yeh et al., 2006, 2009; Seifert et al., 2008; Russell et al., 2010; McFarland et al., 2011; Suarez et al., 2013), all contribute to the ECM overexpression and the resulting dermal protuberance that defines these scars (see Supplementary Table S3B). Increased levels of collagen I (Uitto et al., 1985; Ala-Kokko et al., 1987; Lee et al., 1991; Friedman et al., 1993; Sato et al., 1998; Chipev et al., 2000; Daian et al., 2003; Hasegawa et al., 2003; Lim et al., 2003; Hsu et al., 2006, 2018; Xia et al., 2007; Zhang G. et al., 2009; Dienus et al., 2010; McFarland et al., 2011; Lin et al., 2015; Suarez et al., 2015; Fang et al., 2016; Luo et al., 2017), a major constituent of the dermis, is likely responsible for the bulk of this increased tissue mass. Additionally, there is an increased collagen I:III ratio (Uitto et al., 1985; Abergel et al., 1987; Lee et al., 1991; Friedman et al., 1993; Zhang G. et al., 2009), despite variable reports on the levels of collagen III (Clore et al., 1979; Uitto et al., 1985; Ala-Kokko et al., 1987; Lee et al., 1991; Sato et al., 1998; Lim et al., 2002, 2003, 2013; Zhang G. et al., 2009; Lin et al., 2015; Hsu et al., 2018). The most reported trend is that of increased collagen III levels (Ala-Kokko et al., 1987; Lee et al., 1991; Sato et al., 1998; Lim et al., 2003, 2013; Zhang G. et al., 2009; Hsu et al., 2018). Other ECM constituents also expressed at higher levels in keloid fibroblasts include fibronectin (Kischer and Hendrix, 1983; Babu et al., 1989; Kischer and Pindur, 1990; Sible et al., 1994; Blume-Peytavi et al., 1997; Chipev and Simon, 2002; Liang et al., 2013; Suarez et al., 2015; Fang et al., 2016; Luo et al., 2017; Hsu et al., 2018), elastin (Russell et al., 1989, 1995; Lee et al., 1991), glycosaminoglycans (Berman and Duncan, 1989; Suarez et al., 2013), and both small and large proteoglycans (Yagi et al., 2013).

The profibrotic characteristics of keloid fibroblasts are at least in part, mediated by increased levels of several key wound healing mediators and their associated receptors (see Supplementary Table S3B). Major pathways upregulated in keloid fibroblasts include TGF-β1 (Mccormack et al., 2001; Mikulec et al., 2001; Carroll et al., 2002; Carroll and Koch, 2003; Funayama et al., 2003; Hanasono et al., 2003; Xia et al., 2004; Bock et al., 2005; Fujiwara et al., 2005b; Bran et al., 2010; Lim et al., 2013; Wang et al., 2013; Jurzak et al., 2014; Yang et al., 2014; Lin et al., 2015; Suarez et al., 2015; Fang et al., 2016), TGF-β2 (Xia et al., 2005; Bran et al., 2010; Suarez et al., 2015; Fang et al., 2016), and their receptors (Chin et al., 2001; Xia et al., 2004; Tsujita-Kyutoku et al., 2005); CTGF (Xia et al., 2004, 2007; Khoo et al., 2006; Russell et al., 2010; Jurzak et al., 2014; Fang et al., 2016); VEGF (Wu et al., 2004; Fujiwara et al., 2005b; Ong et al., 2007b; Dienus et al., 2010); interleukins IL-6 (Xue et al., 2000; Ghazizadeh et al., 2007; Do et al., 2012) and IL-8 (Do et al., 2012); as well as IGF-1 receptor (Yoshimoto et al., 1999; Ohtsuru et al., 2000; Phan et al., 2003; Hu et al., 2014) and its binding-related proteins (Phan et al., 2003; Seifert et al., 2008; Smith et al., 2008; Russell et al., 2010). Moreover, keloid fibroblasts not only produce higher levels of wound healing factors, they are also inherently more sensitive to the effects of many of these factors (see Supplementary Table S3B). Keloid fibroblasts showed increased collagen secretion, PAI-1 and PDGFα receptor expression, as well as increased proliferation and migration in response to IL-18 (Do et al., 2012), VEGF (Wu et al., 2004), TGF-β1 (Messadi et al., 1998), HDGF (Ooi et al., 2010), and CTGF (Luo et al., 2017), respectively, which was absent in normal fibroblasts. Similarly, keloid fibroblasts exhibited a greater response in ECM synthesis, proliferation, migration, invasion and inflammatory mediator secretion to TGF-β (Bettinger et al., 1996; Daian et al., 2003), HGF (Jin, 2014), PDGF (Haisa et al., 1994), and IL-18 (Do et al., 2012) stimulation, respectively, compared with normal skin fibroblasts.

In a similar fashion to normal fibroblasts (Kroeze et al., 2009), keloid-derived fibroblasts display mesenchymal stem cell (MCS) markers and possess the multipotency to differentiate into adipocytes, osteocytes, chondrocytes, smooth muscle cells, endothelial cells, and neural lineage cells (Moon et al., 2008; Iqbal et al., 2012; Plikus et al., 2017); thereby earning the descriptor of multipotent precursor cells. Interestingly, multipotency capabilities may differ between different scar types as demonstrated by the ability of keloid fibroblasts, but not their hypertrophic counterparts, to differentiate into adipocytes either by stimulation with BMP4 or when co-cultured with human scalp hair follicle cells (Plikus et al., 2017). Iqbal et al. (2010) further differentiated between MSCs of hematopoietic and non-hematopoietic origin, with the majority comprising the non-hematopoietic subtype located in the top and middle areas of the keloids. Regardless of the MSC subtype, however, all MSC markers showed progressive downregulation in culture with increasing cell passage. Based on the similarly progressive loss of the keloid phenotype with *in vitro* serial culturing and the abnormally proliferative nature of keloid fibroblasts, Moon et al. (2008) hypothesized that keloid fibroblasts may be stimulated by the aberrant keloid cytokine milieu to remain in an undifferentiated multipotent and proliferative stem cell state. By extension, Qu et al. (2013) proposed that these keloid stem cells are able to sustain themselves by asymmetric cell division due to their drug resistant and high self-renewing abilities. The continued generation of new aberrant keloid cells then sets the typical tumor-like keloid growth in motion, and also helps explain the high post-therapy recurrence rates. In fact, the pathological keloid microenvironment may also be responsible for generating the keloid stem cells in the first place. Qu et al. (2013) also hypothesized that a pathological niche exists in keloids that is the result of the pre-existing abnormalities in keloid-prone patients, namely the enhanced and persistent inflammatory response and the overexpression of growth factors and their receptors. The multipotent keloid fibroblasts, or rather keloid stem cells, are then transformed from normal dermal stem cells after exposure to this pathological keloid niche. Akino et al. (2008) co-culture experiments of mesenchymal stem cells with keloid fibroblasts may support this niche hypothesis, as mesenchymal stem cells showed similar fibrotic and myofibroblast-like changes after exposure to keloid fibroblasts in co-culture. Regardless of their cell of origin however, the multipotent stem cell nature of the keloid fibroblast appears to play an important role in the genesis and maintenance of keloid scars.

Although highly informative, findings from fibroblast monolayer cultures are not without their limitations. Serial culturing, methods of fibroblast cell isolation (enzymatic vs. explant^∗^), presence or absence of serum in culture medium, and 3D vs. monolayer culturing, are all potential culturing artifacts which have differed across studies and may influence outcome parameters significantly. It is important to consider the potential confounding effects of these culturing differences while interpreting different results. As a final consideration, it should be noted that keloid fibroblasts are usually compared with fibroblasts derived from healthy non-lesional skin, while in fact normotrophic scars represent the true standard against which keloids should be compared. This holds true for all the tissue and cellular components studied in keloid research, and is not limited to the comparison of keloid fibroblasts to normotrophic scar-derived fibroblasts. In conclusion, keloid fibroblast monocultures have generated a multitude of interesting findings, but it is important to remember the inherent limitations associated with monoculture model systems and consider the influence of differences in cell isolation and culture methods.

##### Abnormal keloid keratinocyte-fibroblast interactions

We know that the interactions between keratinocyte and fibroblasts are an integral component of the normal wound healing process (Lorenz and Longaker, 2003; Broughton et al., 2006) and findings from *in vitro* double chamber co-culture experiments have been particularly informative in this regard, as they allow us to study indirect paracrine interactions between the two cell populations (see Supplementary Table S4D). Co-cultures of keratinocytes with fibroblasts show increased proliferation, levels of ECM and growth factor expression compared with monocultures (Phan et al., 2002, 2003; Funayama et al., 2003; Lim et al., 2003; Xia et al., 2004; Khoo et al., 2006; Ong et al., 2007b; Ooi et al., 2010; Do et al., 2012), but this effect is generally greatest with keloid-derived cells. Keloid keratinocytes are able to induce the profibrotic keloid phenotype in normal skin fibroblasts (Lim et al., 2001, 2002, 2003; Funayama et al., 2003; Xia et al., 2004; Khoo et al., 2006; Ong et al., 2007b; Chua et al., 2011), while keloid fibroblasts are able to the propagate fibrosis markers even when co-cultured with normal keratinocytes (Lim et al., 2001; Phan et al., 2003; Xia et al., 2004; Supp et al., 2012b; Lee Y.-S. et al., 2015).

These findings all strongly support a role for abnormal keloid keratinocyte/fibroblast interactions in the pathogenesis of keloids and thereby provide a new point of interception for therapeutic strategies. In this light, Burd and Chan (2002) described an interesting case of a pediatric patient with a giant keloid covering most of the upper right leg and buttocks. In a multistep procedure, the keloid tissue was removed and an artificial dermal matrix was placed on the wound bed. This was followed by the addition of an autologous keratinocyte cell suspension fixated by a fibrin glue spray. During the 18-month follow-up there was no recurrence of keloid formation. This case report serves as an excellent example of a bench to bedside approach to negate the adverse keloid epidermis-dermis interaction, by removing the diseased dermal matrix and introducing normal keratinocytes.

##### Keloid myofibroblasts

Although Ehrlich et al. (1994) put forward the absence of myofibroblasts as a feature that differentiates keloids from hypertrophic scars, the opposite has also been observed (Santucci et al., 2001). In fact, the overwhelming majority of studies report the presence of α-SMA+ myofibroblasts in 33–81% of the keloids analyzed (Santucci et al., 2001; Kamath et al., 2002; Amadeu et al., 2003; Lee J. Y. Y. et al., 2004; Moshref and Mufti, 2009). Particularly when cultured *in vitro*, keloid fibroblasts can be shown to contain a significant portion of myofibroblasts (Chipev et al., 2000; Chipev and Simon, 2002; van der Slot et al., 2004; Ong et al., 2007a; Lee et al., 2012; Jin, 2014; Suarez et al., 2015; Luo et al., 2017; Shang et al., 2018). In our histopathological study on abnormal scar types and immature scars (3–5 weeks old), we identified a CD34−/α-SMA+ specific dermal cell population, which were largely senescent in the abnormal scars (p16+) but actively proliferating in the young scars (Ki67+) (Limandjaja et al., 2019). See Supplementary Tables S2, S3B for a summary of the histopathological results and cellular abnormalities, respectively.

In wound healing by secondary intention, macrophages stimulate wound bed-derived fibroblasts with TGF-β1 and PDGF to transform them into myofibroblasts (Broughton et al., 2006). In a recently published review, Lim et al. (2019) suggested that the aberrant fibroblasts and myofibroblasts in keloids may originate from an altogether different cell type, namely the embryonal stem cell-like cell population located in the endothelium of microvessels and on the perivascular cells within keloid-associated lymphoid tissue. After injury, these cells are thought to differentiate into abnormal fibroblasts and myofibroblasts through the process of endothelial-mesenchymal transition. Additionally, circulating fibrocytes or mesenchymal stem cells from the bone marrow may also migrate to the target site to generate the abnormal (myo-)fibroblast population. In other words, myofibroblasts in the keloid environment may have several sources of origin beyond the wound bed fibroblasts. Alternatively, mesenchymal stem cells may also serve as a myofibroblast source. Monolayer co-culture experiments with mesenchymal stem cells and keloid fibroblasts have shown that the latter are able to induce a myofibroblast-like phenotypic switch of the mesenchymal stem cells (Akino et al., 2008). In short, myofibroblasts may very well originate from several different sources in addition to the wound-bed fibroblasts.

In normal wound healing processes, we know that myofibroblasts can produce significant wound surface reduction through wound contraction (Lorenz and Longaker, 2003), but Plikus et al. (2017) published an interesting new theory on how myofibroblasts may contribute to the development of keloid scars. They found that hair follicles are essential for inducing myofibroblast-to-adipocyte reprogramming that allows for regeneration rather than scar formation. As hair follicles are absent from the keloid microenvironment, the myofibroblasts are left unable to convert to adipocytes, thereby triggering the scar response leading to keloid formation. In this way, hair follicles and adipocytes may be involved in keloid scarring by effecting myofibroblast dissipation from the wound bed, and serve as interesting new potential therapeutic targets for further research.

##### Keloid fibrocytes

Bucala et al. (1994) were the first to suggest that the surrounding connective tissue may not be the sole source of new fibroblasts in wound repair and described a blood-borne cell with fibroblast-like properties that enter sites of tissue repair (see Supplementary Tables S1, S3B). These so-called fibrocytes were characterized by a collagen+/vimentin+/CD34+ phenotype and not only produced ECM proteins and wound healing mediators, but were also capable of acting as antigen-presenting cells and differentiating into myofibroblasts (Bucala et al., 1994; Quan et al., 2004). Based on the limited available literature, there are increased numbers of CD45RO+/35F9+/MRP8/9+ fibrocytes in keloids compared with normotrophic scars (Iqbal et al., 2012), and moreover, PBMCs derived from keloid patients produced more LSP-1+/collagen1+ fibrocytes than PBMCs from healthy controls (Naylor et al., 2012). In further support of this, Mathangi Ramakrishnan et al. (2012) showed that keloid fibroblasts expressed increased levels of fibrocyte markers (CD34+/CD86+), which were absent in normal fibroblasts. This suggests an at least partial fibrocyte origin of the keloid-derived fibroblasts. Reduced fibrocyte numbers have also been reported (Ueda et al., 1999), but this was based on the presence of histologically identified slender nuclei rather than immunohistochemical phenotyping, and may therefore not be as reliable. Given the aforementioned findings of increased fibrocyte presence in keloids (Iqbal et al., 2012; Mathangi Ramakrishnan et al., 2012) and their potential differentiation into abnormal keloid myofibroblasts (Lim et al., 2019), fibrocytes may be significantly involved in keloidogenesis and therefore deserve further investigation.

##### Keloid endothelial cells

Both increased (Ehrlich et al., 1994; Amadeu et al., 2003; Tanaka et al., 2004; Materazzi et al., 2007; Ammendola et al., 2013; Bakry et al., 2014) and decreased (Beer et al., 1998; Ueda et al., 2004; Bux and Madaree, 2010; Kurokawa et al., 2010) vasculature have been reported in keloid scars in approximately equal measure (see Supplementary Tables S2, S3C). However, based on reports of microvessel occlusion and increased expression of hypoxia-induced factor 1α (HIF-1α) in abnormal scars, it has been proposed that keloids are relatively hypoxic tissues (Zhao et al., 2017). The ischemia hypothesis builds on this to explain how hypoxia can contribute to keloid scar development. Kischer et al. (1982) demonstrated that unlike normal skin, the overwhelming majority of hypertrophic and keloid scars have microvessels with occluded lumens and that this was likely due to endothelial cell proliferation. The authors considered this reaffirmation of their hypothesis that hypoxia “*is an integral factor in the generation of hypertrophic scar and keloid*” (Kischer et al., 1982), but whether or not this relative hypoxia promotes fibroblast and endothelial cell proliferation has still not been determined (Song, 2014).

Kischer (1984) also suggested another mechanism by which the microvessel abnormalities could generate both hypertrophic scars and keloids. They proposed that injury leads to regeneration of the microvessels, and suggest that the pericytes of the newly regenerating microvessels form the source of the fibroblasts generating the excessive collagen which characterizes these abnormal scars.

It has also been suggested that endothelial cell dysfunction plays a role in keloidogenesis. Ogawa and Akaishi (2016) proposed that local factors such as stretching tension together with genetic factors both act to induce endothelial cell dysfunction in the form of vascular hyperpermeability during the inflammatory phase of wound healing. This prolongs the influx of inflammatory cells and factors, thereby also prolonging the inflammatory phase. Consequently, dysfunction of the fibroblast cell population leads to the development of either hypertrophic or keloid scars. Lastly, endothelial cells may also contribute to keloid scar development by undergoing endothelial-mesenchymal transition (EndoMT) to acquire a mesenchymal phenotype (Lee Y.-S. et al., 2015). In this way, endothelial cells may directly serve as a source of the abnormal keloid fibroblasts.

##### Keloid nerve cells

Based on the symptoms of itching and pain, both sensations carried by small nerve fibers, there does appear to be a role for nerve cells in the development of keloid scars. Yet, thus far little has been published on the presence of nerve cells in keloid tissue (see Supplementary Tables S2, S3C). Sensory nerve fibers have also been mentioned in the context of Ogawa’s mechanobiology theory on keloid pathogenesis (Ogawa, 2011). As part of the group of skin receptors perceiving mechanical forces, information from the sensory fibers is then relayed to the central nervous system leading to the release of neuropeptides, which can then modulate scarring by altering skin and immune cell functions. However, studies staining for nerve fibers in keloid tissue have reported both increased (Hochman et al., 2008; Drummond et al., 2017) and decreased (Saffari et al., 2018) nerve fiber densities. As different markers (PGP9.5 and S100 protein, respectively) were used to identify the nerve fibers, this could in part explain the different outcomes.

#### Keloid Immune Cells

Although both increased and reduced levels of certain immune cell types have been reported (see Supplementary Tables S2, S3D), overall there appears to be an increase in macrophages (Boyce et al., 2001; Shaker et al., 2011; Bagabir et al., 2012a; Jiao et al., 2015), T-lymphocytes (Boyce et al., 2001; Shaker et al., 2011; Bagabir et al., 2012a; Jiao et al., 2015) and mast cells (Kamath et al., 2002; Moshref and Mufti, 2009; Shaker et al., 2011; Bagabir et al., 2012a; Ammendola et al., 2013) in keloids that have been found to interact with each other, other immune cells and dermal fibroblasts on a cellular level (Boyce et al., 2001; Santucci et al., 2001; Shaker et al., 2011). Moreover, macrophages and T-lymphocytes from keloids also showed intrinsic abnormalities compared with their normal counterparts. Keloid-derived macrophages showed a high activation status, increased M2 polarization and overall increased expression of both M1 and M2 activation factors compared with normal skin macrophages (Jin et al., 2018). They were also more potent at inducing the regulatory T-cell phenotype when co-cultured with CD4+ T-lymphocytes from keloid patients’ blood. Thus, there was not just a general increase in T-lymphocytes, but specifically the regulatory (Jin et al., 2018) and memory (Chen et al., 2018) T-cells, as well as an increased CD4+:CD8+ ratio (Boyce et al., 2001; Bagabir et al., 2012a) in keloids. Furthermore, the altered cytokine production in keloid-derived memory T-cells (Chen et al., 2018) and the reduced mitogenic response of circulating T-cells to known mitogenic stimuli (Bloch et al., 1984), suggests that an abnormal T-cell response may contribute to keloid scarring. In this way, the previously discussed sebum reaction hypothesis is also an extension of this concept as the intrinsically abnormal, sebum-sensitive T-lymphocytes take center stage in this theory (Song, 2014).

Mast cells have also been found in abundance in keloid scars. Arbi et al. (2015) found that mast cells were closely associated with fibroblasts in keloid scars and that the phagocytosis of collagen fibrils by mast cells was a common ultrastructural feature. They hypothesized that the abnormal collagen synthesis observed in keloids and the consequent accumulation of collagen fibers, are able to induce increased mast cell recruitment and subsequent collagen phagocytosis. The resulting release of mast cell-derived mediators (interleukins, mediators, and growth factors) is then able to stimulate further collagen production and thereby aids further keloid scar development. Lastly, there have been variable reports on the presence of Langerhans cells in the epidermal compartment of keloid scars, both normal (Bagabir et al., 2012a) and increased (Jiao et al., 2015) numbers have been observed in both hypertrophic and keloid scars.

Several hypotheses centering on inflammatory processes have also been put forward. In the chronic inflammation hypothesis, Dong et al. (2013) posed that the presence of chronic inflammation in keloids indicates that local inflammation promotes keloid formation. The traumatic and inflammatory stimuli that trigger keloid scar formation result in the continuous upregulation of already highly sensitive pro-inflammatory genes in the keloid microenvironment. This keloid microenvironment fosters the development of abnormalities in the resident keloid fibroblast, which in turn is considered to be the driving force behind keloid scar formation. Ogawa (2017) has further expanded on this notion and postulated that chronic inflammation is responsible for the invasive growth of keloids and even suggests that both hypertrophic scars and keloids are principally inflammatory disorders of the reticular dermis rather than being skin tumors. In the neurogenic inflammation hypothesis, the inflammation is thought to arise from mechanical stress, such as skin stretching, which stimulates mechanosensitive receptors on sensory fibers to release neuropeptides. These then bind to receptors of various skin cell types including keratinocytes and fibroblasts, mast cells and endothelial cells. Vasodilation and vessel permeabilization, increased mast cell release of histamines and cytokine production (including TGF-β) take place as a result of this. Fibroblasts then become activated as a result of the neurogenic inflammation and the upregulation TGF-β, leading to keloid and hypertrophic scar formation (Akaishi et al., 2008).

### Other Proposed Hypotheses on Keloid Scar Formation

The myriad of available treatment modalities is only matched by the multitude of proposed hypotheses to explain keloid scar formation. These are not mutually exclusive, and further support the notion that keloid scarring has a multifactorial genesis (Slemp and Kirschner, 2006). When appropriate, these have already been discussed in the appropriate paragraphs of the section on keloid cellular abnormalities. A brief discussion of additional hypotheses that could not be categorized in the previous paragraph, will follow next.

#### Keloid Triad Hypothesis

Perhaps one of the only proposed hypotheses encompassing multiple risk factors in one, the keloid triad hypothesis (Agbenorku et al., 1995) is defined as a group of three etiologic factors: genetic links, infective agent (bacterial, viral, or other) and surgery (e.g., sutures, tension of suture lines, location of sutures in relation to the relaxed skin tension lines); which must be simultaneously present and interact to develop keloid scarring. These three factors are further subdivided into major factors and minor etiological factors. Major factors include: African ethnicity, age 10–30 years, familial susceptibility or keloid-prone upper part of body site. Minor factors include: orientation of incisions/sutures with respect to RTSLs, wound or sutures under tension, healing by secondary intention, type of infection; determines whether or not a keloid scar is likely to develop. At least one major and two minor factors must be present for keloid scars to develop. A keloid is unlikely to develop if all three factors are minor or if only two factors are present, but a hypertrophic scar may form instead.

#### Incomplete Malignancy Hypothesis

When Ladin et al. (1998) studied apoptosis in keloid scars, they found that the level of apoptotic cells was significantly reduced in keloid tissue and fibroblasts compared with normal foreskin tissue and fibroblasts. However, keloid fibroblasts did show increased apoptosis upregulation in response to treatment with hypoxia, hydrocortisone or γ interferon, while normal fibroblasts were only responsive to high doses of hydrocortisone. Because of this, Ladin et al. (1998) suggested that keloids may represent a type of incomplete malignancy that has undergone some, but not all tumourigenic changes.

#### Viral Hypothesis

In this infection-based hypothesis (Alonso et al., 2008), the authors proposed a role for a normally quiescent, unknown virus in the bone marrow or lymphatic system which is activated in a genetically susceptible person with a wound. This virus can then reach the wound via fibrocytes that are chemoattracted to the wound site or via infecting virions in the saliva arriving at the wound bed. There the many chemical stimuli from the wound healing processes allow the virus to become activated, resulting in transcription of viral proteins which derail wound healing and eventually lead to keloid scarring.

#### Stiffness Gap Hypothesis

Born out of recent findings on the role of mechanotransduction in keloid scarring, this theory (Huang et al., 2017) proposes that the enlarged gap between ECM stiffness and cellular stiffness enables the constant and continued keloid progression. The ECM is not only a cellular scaffold storing important wound healing mediators, but its rigidity also influences fibroblast function and can induce processes such as fibroblast migration, proliferation and differentiation. When dermal fibroblasts sense the stiffness gaps between the ECM and themselves via mechanotransduction, alterations in fibroblast phenotype ensue which promote proliferation, migration and ECM synthesis and therefore contribute to keloid progression. However, systematic mechanobiological experiments to verify this hypothesis have yet to be performed.

#### Immunonutritional Hypothesis

It has been proposed by several authors that nutritional imbalances can promote prolonged inflammation and cytokine-mediated reactions which contribute to keloid scarring (Huang and Ogawa, 2013a). Nutritional imbalances have been reported for essential fatty acids and micronutrients in keloid-prone patients. For example, the keloid-prone black South African population were deficient in the essential fatty acids a-linolenic acid, eicosapentaenoic acid and docosahexaenoic acid, but showed an increased intake of linoleic acid and arachidonic acid. The levels of micronutrients (calcium, copper, iron, vitamin B2, vitamin C, vitamin A, and zinc) was also insufficient in their diet (Louw and Dannhauser, 2000), and this was also reflected in the serum as well as keloid scar tissue (Bang et al., 2002). Compared with non-keloid forming patients, keloid-prone patients showed higher levels of zinc in their serum, higher copper levels in non-lesional normal skin and in the keloid scar tissue. The essential fatty acid imbalance has also been observed in the membrane of keloid fibroblasts with lower linoleic acid and higher neutral lipid levels in the central keloid area compared with its periphery (Louw et al., 1997). Lipids are not only essential cell membrane constituents, but also have important roles in local inflammation and intracellular signal transduction. As such they are also active participants in the chronic inflammatory processes that result in the development and continued growth of keloids (Huang and Ogawa, 2013b). And finally, dietary compounds have been shown to affect keloid-derived cells at the *in vitro* level as well. Green tea and its extracts have been shown to reduce keloid fibroblast proliferation, migration and collagen synthesis (Zhang et al., 2006; Park et al., 2008).

#### Metabolic Hypothesis

It has also been theorized that the net overexpression of ECM components in the keloid dermis is also the result of both the increased number of fibroblasts and their increased intrinsic metabolic activity, aside from the usual suspects in abnormal signaling pathways (Butler et al., 2008a). Both keloids and hypertrophic scars showed increased numbers of fibroblasts compared with normal scars or normal skin (Ueda et al., 1999), but only keloid fibroblasts showed increased metabolic activity. Metabolic activity has also been measured as levels of ATP (Ueda et al., 1999; Vincent et al., 2008) and as total protein content synthesis together with endoplasmic reticulum staining (Meenakshi et al., 2005), all of which have proven to be upregulated in keloids compared with normal skin, normotrophic and hypertrophic scars. Modulating keloid metabolism may therefore be a way to reduce keloid scar growth (Butler et al., 2008a; Huang and Ogawa, 2013a).

#### Nitric Oxide Activity

Using mathematical modeling, Cobbold (2001) proposed that nitric oxide could be involved in both hypertrophic and keloid scar formation. Nitric oxide is a known source of free radical molecules which stimulates collagen synthesis in normal wound healing, it therefore follows that excessive amounts of nitric oxide can result in the elevated scar tissue characteristic of these abnormal scar types. Cobbold further speculated that the source of this excessive nitric oxide may be the basal epidermis, in part because the generated free radicals also stimulate melanin synthesis and melanocytes are located within this epidermal layer.

#### Psychoneuroimmune-Endocrine Hypothesis

Hochman et al. (2015) posed that the “brain-skin connection” may play a role in keloid scarring. The psychogenic component is a part of the pathogenesis in most skin diseases such as psoriasis and atopic dermatitis, where it can trigger integrated responses from the nervous, immune and the endocrine systems. Normal wound healing also depends on neurogenic factors, which in turn are influenced by psychological, immune and endocrine factors. Any change in these factors may interfere with the scar formation process. Hochman speculated that stressed patients themselves exacerbate the neuro-endocrine system, leading to the release of hormones, neural transmitters, and immune cells which creates an inflammatory microenvironment that stimulates fibrotic processes (Hochman et al., 2015). This has not been extensively studied for abnormal scars but, stress as measured by the sweating response during a stress-inducing task was found to be associated with an increase in keloid recurrence rates after surgery with postoperative radiotherapy (Furtado et al., 2012).

#### Hypertension Hypothesis

Dustan (1995) first hypothesized that the differences in growth factor abnormalities that are conducive to keloid scarring in the black population, could also be responsible for the development of hypertension and therefore explain the differences in hypertension severity between the black and white population. A significant increase in hypertension rates has in fact been reported in keloid-prone patients compared with non-keloid formers in both the African–American (Snyder et al., 1996; Adotama et al., 2016; Rutherford and Glass, 2017) and Caucasian population (Snyder et al., 1996). Ogawa et al. (2013) also reported that severe keloid cases (multiple or large >10 cm^2^) in Japan were significantly more likely to have hypertension than patients with mild keloids (<2 or <10 cm^2^). Furthermore, in patients with keloids, those with more severe hypertension were significantly more likely to have multiple and/or larger keloids than patients with normotension or less severe hypertension (Arima et al., 2015). The mechanisms by which hypertension could lead to keloid development remain largely speculative for the time being, but the changing blood flow likely affects the skin cellular constituents in ways that promote fibrotic processes (Huang and Ogawa, 2014). Angiotensin-converting enzyme (ACE) was proposed as a potential common mechanism for the pathogenesis of both keloids and hypertension, but Stewart and Glass (2018) found no correlation between plasma ACE levels and keloids or hypertension. Nevertheless, antihypertensives such as ACE-inhibitors (e.g., captopril), calcium-channel blockers (e.g., verapamil) have been able to reduce keloid symptoms and are part of the current therapeutic arsenal for keloid treatment. Huang and Ogawa (2014) reviewed the evidence for the participation of systemic hypertension in hypertrophic and keloid scar pathogenesis and although they could not establish a strong causal relationship, hypertension was considered a likely risk factor for abnormal scarring.

## Models of Keloid Scarring

**FIGURE 3:**
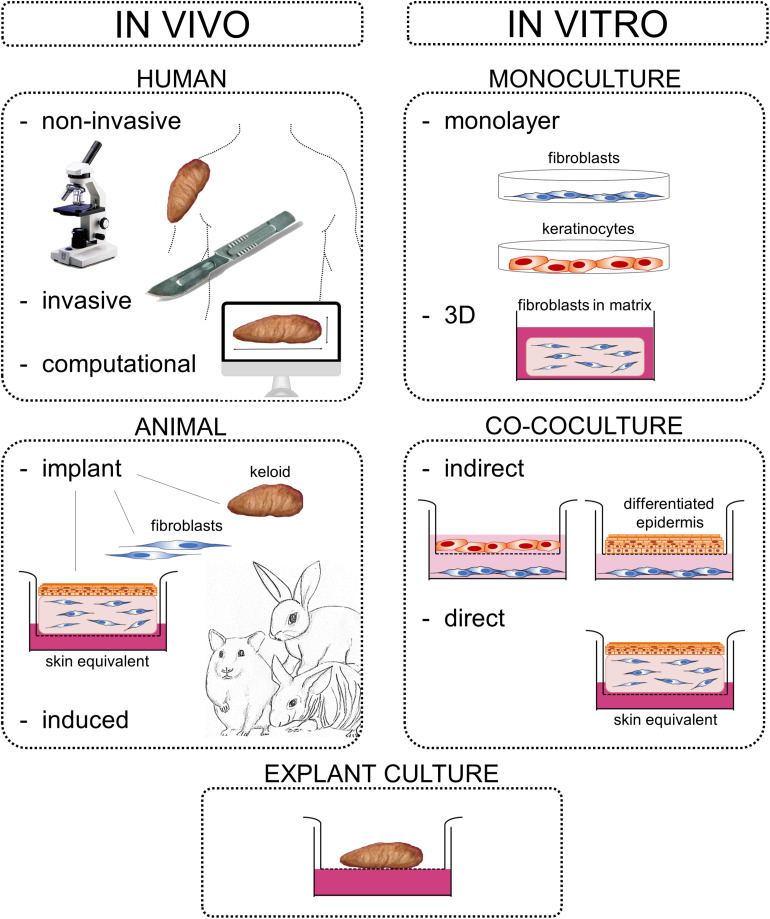
Keloid scar models. Overview of available keloid scar models. Keloid models can be further subdivided into *in vivo* and *in vitro* models, which can be of human or animal origin. Human *in vivo* models include non-invasive testing methods (e.g., imaging and microscopy) and invasive methods, such as serial biopsy or inducing keloid formation by wounding. Animal *in vivo* models include implantation of keloid tissue fragments, fibroblasts or full thickness skin equivalents, as well as inducing keloid scar development by irritation or wounding. *In vitro* keloid models range from simple monolayers to 3D structures and co-culture systems. Indirect co-culture systems include monolayer keloid fibroblasts combined with either monolayer keratinocytes or a fully differentiated epidermis. Lastly, explant cultures are a combination of *in vivo* and *in vitro* as the method involves the maintaining of keloid tissue fragments in *in vitro* culture. Figure first published in Limandjaja (2019), used with permission.

Keloid scar models can largely be divided into *in vivo* and *in vitro* models, Supplementary Tables S4A–E and Figure 3 give an overview of the currently available keloid scar models.

### *In vivo* Keloid Models

*In vivo* keloid models are of the human or animal variety. Currently available *in vivo* human keloid models (Supplementary Table S4A) can be further subdivided into non-invasive, invasive, and computational models. Non-invasive *in vivo* modeling generally comprises live imaging whereby certain tissue characteristics are visualized. Invasive keloid modeling varies from the relatively minor FDG injections to measure glucose metabolism (Ozawa et al., 2006), to serial biopsies to evaluate keloid development over time (Lin et al., 2015) and even includes attempts to induce keloid formation in known keloid formers by wounding (Mancini and Quaife, 1962). Despite their value especially in the clinical setting for follow-up evaluation, human *in vivo* models post-1962 are inherently limited in the ability to manipulate experimental variables and yield primarily observational data. While Lebeko et al. (2019) saw particular merit in the temporal data acquired from serial keloid biopsy analysis as an *in vivo* ‘4D model’ in their review on keloid models, it is important to recognize the potential risk of exacerbating the existing keloid scars by continual provocation with biopsy-associated injury.

In animal models, keloid formation is either induced or involves the implantation of human keloid tissue (see Supplementary Table S4C). Unfortunately, inducing keloid scar formation has proven virtually impossible (Seo et al., 2013) and more often than not, hypertrophic scars developed instead of keloids (Morris et al., 1997; Khorshid, 2005). Implanting human keloid cells or tissue fragments into animal models has been more successful (Hillmer and Macleod, 2002) and resulted in the development of a palpable nodule-like mass. While implanted keloid tissue was generally able to retain the keloid-specific keloidal collagen even within the animal model, the use of already established keloid tissue does not allow us to study *de novo* keloid development. However, when tissue culture techniques were combined with IL-6 exposure to implant a keloid fibroblasts-hydrogel suspension into nude mice, the resultant mass not only showed the greatest increase in growth, but also gave rise to *de novo* keloidal collagen formation (Zhang Q. et al., 2009). The appeal of an *in vivo* animal model is obvious, but there is an important limitation to the use of any animal for the study of keloid scarring, in that keloids occur exclusively in humans (Tuan and Nichter, 1998; Yang et al., 2003). Though exuberant granulation tissue on horse limbs has often been posed as the equine version of keloid tissue, a recent study (Theoret et al., 2013) comparing keloids and exuberant granulation tissue has shown they are not in fact one and the same. Fundamental differences in essential skin physiology (hair follicle density, epidermal, and dermal thickness) and wound healing mechanisms between rodents and humans (Perez and Davis, 2008; Ramos et al., 2008) pose additional important limitations. The *in vivo* microenvironment in animal models may therefore provide a less than human-like exposure to the keloid cell population.

### Keloid Explant Models

Keloid tissue explants do not necessarily require implantation into an animal model in order to survive and be used as a keloid model in and of itself, they can be maintained in culture after removal from the human body for up to 6 weeks (Duong et al., 2006; Bagabir et al., 2012b) (see Supplementary Table S4B). Various culture methods have been tried, but keloid morphology appears best conserved in explants embedded in collagen gel that are cultured air-exposed (Duong et al., 2006; Bagabir et al., 2012b; Mendoza-Garcia et al., 2015). Unfortunately, relying on fresh keloid tissue for this explant model system has its obvious logistical issues with limited availability and potential inter- and intralesional variability between samples. Another important limitation of this model type is the absence of circulatory system, as is the case with all *in vitro* models to date. Ultimately, this model system appears best suited for the testing of therapeutics (Syed et al., 2012, 2013a,b) rather than studying the pathogenetic mechanisms underlying keloid formation, as there is very limited ability to manipulate any experimental variables.

### *In vitro* Co-culture Keloid Models

As skin physiology, immunology and wound healing is markedly different in animals (Hillmer and Macleod, 2002; Ramos et al., 2008; Seo et al., 2013; Seok et al., 2013) and since keloids are exclusive to humans (McCauley et al., 1992; Yang et al., 2003), there is a need for relevant human *in vitro* models (see Supplementary Tables S4D, E). What is more, *in vitro* co-culture systems allow for extensive experimental manipulation, such as the development of heterotypic models combining normal keratinocytes with keloid fibroblasts to study the individual contribution of keloid fibroblasts to keloid formation.

Indirect co-cultures consist of double chamber systems with keratinocytes seeded (as a monolayer or differentiated epidermis) onto an upper porous transwell insert and fibroblasts cultured in a monolayer on the underlying bottom well (see Supplementary Table S4D). These indirect co-cultures are simple-to-execute experiments that are particularly suited to study paracrine interactions, as previously discussed in ‘Abnormal Keratinocyte-Fibroblast Interactions’. However, the monolayer fibroblast cultures and the artificial separation between keratinocytes and fibroblasts, bear little resemblance to the true *in vivo* situation.

Direct co-cultures represent the most *in vivo*-like *in vitro* culture model and include either mixed monolayer cultures or full thickness skin equivalents comprised of keloid-derived cells (see Supplementary Table S4E). The full thickness skin equivalents comprised entirely of keloid-derived keratinocytes and fibroblasts most closely resemble the native keloid, but thus far, these have only been developed in the context of implantation into an animal model (Supp et al., 2012b; Lee Y.-S. et al., 2015) and/or subjected only to limited experimental analysis (Supp et al., 2012a). Overexpression of ECM components, particularly collagen I, was the common denominator in these keloid models. Recently, we were also able to reconstruct keloids *in vitro* with keloid-derived keratinocytes and fibroblasts, using a collagen-elastin scaffold (Limandjaja et al., 2018a). Compared with *in vitro* reconstructed normal skin, the keloid model showed a trend of increased dermal thickness, increased α-SMA and p16 expression, reduced HGF secretion and reduced collagen type IV α2 chain, hyaluronan synthase 1 and matrix metalloproteinase 3 gene expression. More importantly, the keloid model behaved differently from similarly reconstructed hypertrophic scars, and was able to demonstrate intralesional heterogeneity within keloids (Limandjaja et al., 2018b). In other words, a relatively simple *in vitro* keloid model was already able to demonstrate certain intrinsic abnormalities in keloid-derived keratinocytes and fibroblasts and in doing so, identified certain aspects of keloid behavior which could not have been deduced from *ex vivo* biopsy analysis alone. In our opinion, this both illustrates and validates the use of skin tissue engineering as an important adjunct to furthering keloid scar research.

Ultimately, there is no single universal keloid model that is able to satisfactorily answer every experimental objective (Hillmer and Macleod, 2002; Marttala et al., 2016). Overall, animal models are better suited for therapeutic testing and particularly for safety testing prior to human administration (Hillmer and Macleod, 2002; Marttala et al., 2016), while tissue culture systems are best suited to study keloid pathogenesis (Hillmer and Macleod, 2002; Marttala et al., 2016). Given the exclusive occurrence of keloids in humans (Tuan and Nichter, 1998; Yang et al., 2003) and other serious limitations of using animal models for wound healing studies, as well as the fact that research on its pathogenesis cannot rely solely on (immunohistochemical) analysis of an intact native specimen; it seems pertinent to focus our efforts on further advancing tissue culture models - in particular the *in vitro*, organotypic full thickness keloid skin equivalents. Once proven faithful to actual *in vivo* keloid biology, using this model to study keloid formation will eventually aid in the development of superior treatment methods by uncovering new mechanisms of pathogenesis. In addition, such a model would have the added benefit of serving as a test object for future therapeutic modalities without the complication of ethical objections (Yang et al., 2003).

## The Keloid Disorder

The International Classification of Disease (ICD) represents the international standard for reporting diseases and health conditions and in the most recently released ICD-11 (2018), keloids have been grouped under ‘fibromatoses and keloids,’ “*a heterogenous group of disorders characterized by increased deposition of fibrous tissue in the skin and subcutaneous tissues.*” This is departure from the previous ICD-10 (2016) in which keloids were still categorized under ‘hypertrophic disorders of skin,’ and is the result of recent international agreement to refer to keloids as part of a ‘keloid disorder’ rather than a ‘keloid scar.’ This change in ICD classification and nomenclature reflects growing consensus among keloid researchers and clinicians alike that keloids more closely resemble a benign, fibrous tumor than scar tissue and can best be described in terms of a fibrotic disorder.

By their very definition of continued, invasive horizontal growth (Burd and Huang, 2005), keloids clearly share certain characteristics of cancerous growths. Lu et al. (2007b) have previously described keloids as behaving “*clinically as non-metastatic malignancies, although histologically they are benign.*” Similarly, Ladin et al. (1998) coined keloids ‘incomplete malignancies’ as keloid fibroblasts are still apoptosis-responsive to *in vitro* treatment despite overall reduced levels of apoptosis. The term refers to the manner in which keloids show some, but not all the changes associated with tumourigenesis. The absence of metastasis despite their invasive growth and the aforementioned cancer-like characteristics, separates keloids from true malignant tumors and lends further support to the ‘incomplete malignancy’ term coined by Ladin et al. (1998).

The change in nomenclature reflected in the newest edition of the ICD may seem like an exercise in semantics, but it should be noted that the cosmetic association with the word ‘keloid scar’ significantly complicates both insurance coverage for treatment as well as funding for research. This is not to say this nomenclature change must result in an altogether ban on the association of the word ‘scar’ with keloids, merely that we shift the focus away from a purely cosmetic association, toward broadening our perspective of keloids for the fibroproliferative disorders that they are and in this way recognize the severity of this fibrotic disorder.

Keloid research remains plagued by significant controversy and contradictory findings, which has been highlighted throughout this manuscript where possible. Much of this could be resolved by the standardization of study designs and general research approach. Important considerations for future research include (i) inclusion of a description of the keloid phenotype (based on growth pattern), (ii) clarification of the location within the keloid from which samples were taken (e.g., growing/bulging area versus more quiescent region; peripheral versus central areas), (iii) inclusion of scars of similar maturation stage (at least 1 year old). Lastly, the inclusion of both hypertrophic and normotrophic scars would be a valuable addition to keloid research, as this provides a more comprehensive view of the entire scarring spectrum.

## Author Contributions

GL, SG, and FN conceived the manuscript outline. GL performed the literature study and wrote the manuscript with input from all the authors. SG, FN, and RS supervised, helped shape the manuscript, and offered final critical revision.

## Conflict of Interest

The authors declare that the research was conducted in the absence of any commercial or financial relationships that could be construed as a potential conflict of interest.
