# Supplementary material for: The Keloid Disorder: Heterogeneity, Histopathology, Mechanisms and Models
Source: Front Cell Dev Biol. 2020 May 26;8:360. doi: 10.3389/fcell.2020.00360 (PMC7264387; doi:10.3389/fcell.2020.00360)
Supplement: Supplementary file 1 [file Table_1.DOCX]

**Supplemental table 1.** Histopathology of normal skin and scars

| **Location** | **Parameter** | | | **Nskin** | **Nscar** | **Hscar** | **Kscar** | **References** |
| --- | --- | --- | --- | --- | --- | --- | --- | --- |
| Epidermis | epidermal thickness | | |  |  | ↑ | + | [12, 78, 124, 131] |
|  |  | | | + | + | + / ↑ | + / ↑ | [53, 123, 170] |
|  |  | | | + | + | + | ↑ | [21, 25, 44, 61, 117, 175, 184] |
|  |  | | | + | + | ↑ |  | [74, 119, 161, 203] |
|  |  | | | thin |  |  | ↑ | [91, 113, 148, 169, 181, 182] |
|  |  | | | thin |  | ↑ |  | [171, 209] |
|  |  | | |  |  | ↑↑ | ↑ | [180] |
|  |  | | |  |  |  | ↓ (83%) | [20] |
|  | epidermal thickness (μm) | | |  | ↑ | ↑↑ | ↑↑↑ | [71] |
|  |  | | | + | + | + |  | [46] |
|  |  | | | + |  | ↑ |  | [45, 103, 205] |
|  |  | | | + | ↑ |  |  | [202] |
|  | epidermal thickness (cell layers) | | | +, ± 4 | + | ↑, ± 8 | ↑↑ | [114, 115], [42, 103] |
|  | rete ridges | | | + | + | – | + | [12, 131] |
|  |  | | | + | – | – | – | [20, 53, 78, 115, 124, 169, 186] |
|  |  | | |  |  | ↓ | ↓ | [43, 91, 131, 169, 181] |
|  |  | | | + | ↓ |  | – | [21] |
|  |  | | |  |  | + or – | + or – | [119, 170] |
|  |  | | | + | + | ↓ |  | [46] |
|  |  | | |  |  | + / ↑ |  | [161] |
|  |  | | |  |  | + (60%) | + (93%) | [107] |
|  |  | | |  |  |  | lengthened | [184] |
|  | epidermal appendages | | | + | + | – | + | [12] |
|  |  | | | + | – | – | – | [20, 21, 53, 95, 123, 186] |
|  |  | | |  |  | displaced | displaced | [162] |
|  | differentiation | | K1/K10 | + | + | + | + | [27, 66, 115, 119, 148, 197, 202, 203] |
|  | K2e | | | + |  | + / ↑ | + / ↑ | [27] |
|  | filaggrin | | | + | + | ↑, + | + | [66, 115, 119], [66, 115] |
|  | loricrin, SKALP, SPRR2 | | | +, +, + | +, +, + | +, +, + | +, +, + | [115] |
|  | involucrin | | | + | + | + / ↑ | ↑ | [115] |
|  | transglutaminase | | | + | + | + |  | [66] |
|  | activation | | K6/K16 | –  – | – | ↑↑, –  – (80-90%) | ↑  – (90%) | [27, 66, 119, 148], [66]  [115] |
|  | K17 | | | – | – (90%) | – (90%), + | – (90%) | [66, 115], [66, 119] |
|  | progenitors | K19 | | + |  | ↑, – |  | [32] |
|  | proliferation | | K5/K14  K5 | +  + | ↑  + | ↑↑, +  +, ↓ | ↑ | [119, 153, 202, 203], [119]  [66, 197] |
|  |  | | Ki67 | + | + | +, ↑ | +, ↑ | [26, 27, 115, 148], [26, 27, 148] |
|  | PCNA | | | + |  |  | + | [16] |
|  | apoptosis | | TUNEL | + |  | ↓ |  | [41] |
|  | in situ end labelling/acridine orange | | | + |  |  | ↑ | [16] |
|  | Bcl-2, c-jun | | | +, + |  | +, + | +, + | [188] |
|  | p53 | | | – |  | – | –, + | [70, 188] |
|  | p65 | | | – |  |  | ↑ | [121] |
|  | autophagy LC-3 | | | + |  | ↓ |  | [171] |
|  | caspase-1, caspase-3 | | | +, + |  |  | ↑, ↓ | [49] |
|  | hyaluronan | | | + | + | +/– – | ↑ | [25, 127] |
|  | hyaluronic acid | | | + | ↑, + |  | ↑↑, + | [127, 186], [186] |
|  | hyaluronan synthase, hyaluronidase | | | +, + |  |  | ↑, ↑ | [175], [175] |
|  | tenascin C; biglycan, decorin | | | +; +, +/– |  |  | ↑; +, +/– | [48]; [185], [185] |
|  | basement membrane (BM), continuous | | | + |  | – |  | [200] |
|  | methenamine silver (BM) | | | + | ↓ | – |  | [202, 203] |
|  | collagen IV | | | + | – | – |  | [202, 203] |
|  | laminin-5 | | | + |  | – |  | [203] |
|  | integrin β4 | | | + | – | – |  | [202, 203] |
|  | integrin β1 | | | + |  | ↑, ↓, − |  | [32, 197, 203], [197], [203] |
|  | desmosome components (JUP, PKP1) | | | + |  |  | ↓ | [63] |
|  | melanocytes; melanin | | | +; + | +; + |  |  | [195] |
|  | melanocortin-1 receptor | | | +, – |  |  | ↓↓, ↑ | [116], [132] |
|  | α-MSH, HMB-45, POMC | | | –, –, – |  |  | ↑, –, ↑ | [132], [178], [177] |
|  | vitamin D receptor | | | + |  |  | ↓ | [65] |
|  | EMT-markers | E-cadherin | | + |  | − | +, ↓ | [102, 200], [64, 118, 201] |
|  | vimentin | | | + |  | ↑ | ↑ | [64, 102, 118, 200, 201] |
|  | α-SMA | | | +/– – |  |  | ↑ | [201] |
|  | FSP1; fibronectin | | | –; – |  | ↑ | ↑; ↑ | [200, 201]; [118, 201] |
|  | β-catenin; HIF-1α | | | –; – |  |  | ↑; ↑ | [44, 64]; [118] |
|  | MMP-28, TIMP-1 | | | +, +/– – |  | ↑, ↑ |  | [154], [176] |
|  | PAI-1 | | | + | + |  | ↑ | [191] |

**Supplemental table 1.** Histopathology of normal skin and scars – continued

| **Location** | **Parameter** | | **Nskin** | **Nscar** | **Hscar** | **Kscar** | **References** |
| --- | --- | --- | --- | --- | --- | --- | --- |
| Epidermis | TGF-β1 | | (20%), + | (60%), + | ↑ (90%), + | ↑ (90%) | [2], [57, 67, 69] |
|  | TGF-β, TGFβ3 | | +, + | + | +, + |  | [67] |
|  | TGFβRI, TGFβRII | |  |  | +, + |  | [165] |
|  | SMAD3 | |  |  |  | ↑ | [69] |
|  | CTGF; FGF2 | | +; + |  |  | ↑; ↑ | [96]; [136] |
|  | PDGF; bFGF | | +; + | +; + | ↑; + |  | [142]; [67] |
|  | HGF, c-Met | | +, + |  |  | ↑, ↑ | [135] |
|  | HDGF | | + |  |  | + / ↑ | [151] |
|  | TNF-α, IFN-γ | | –, + | +, + | +, + |  | [143], [142] |
|  | IL-1α | | – | ↑ | + |  | [142] |
|  | IL-1β | | +, – | + | ↑ , + |  | [142, 161], [142] |
|  | IL-4, IL-15 | | +, – | +/– –, – | ↑, ↑ |  | [143], [35] |
|  | IL-18, IL-18Rα | | +, + |  |  | ↑, ↑ | [49] |
|  | IL-31, IL-31RA, OSMR | | +, +, + |  | ↑, ↑, ↑ |  | [205] |
|  | IGF-1  IGF-1R | | +  + (90%) |  | +  + (80%) | + (90%) | [56]  [77, 146, 204] |
|  | VEGF | | + | + | ↑, + | ↑ | [32, 67, 149], [67] |
|  | Ang 1, Ang 2 | |  | –, – | ↑, – |  | [194] |
|  | prothrombin, thrombin, thrombomodulin | | +, +, + | +, ↑, ↑ |  |  | [18] |
|  | PAR-1 | | +, +/– – | + |  |  | [18, 123], [123] |
|  | PAR-2 | | +/– – | + |  |  | [123] |
|  | CD34; c-KIT | | –; +, +/– |  |  | –; +, ↑ | [20]; [20, 133], [133] |
|  | CD36 | | − |  | ↑ |  | [9] |
|  | HLA-DR, HLA-DQ, IL-2R | | –, –, – | –, –, – | ↑, ↑, ↑ |  | [34] |
|  | galectin-7 | | + |  | ↓ |  | [41] |
|  | pleiotrophin | | +/– – |  | ↑ |  | [209] |
|  | leptin | | + | + | ↑ | ↑↑ | [167] |
|  | adiponectin, adiponectin receptor | | +, + |  |  | ↑, ↓ | [117] |
|  | activin-A, follistatin | | +, + |  |  | ↑, ↑ | [134] |
|  | SPARC, stratifin | | +, + |  | ↑, ↓ |  | [39] |
|  | S100A12 | | + |  | ↑ | ↑ | [212] |
|  | COX-1 | | + | + | ↑, + | ↓, + | [152, 155], [152] |
|  |  | |  | + (40%) | + (53%) | + (100%) | [1] |
|  | COX-2 | | + | + | + | ↑, + | [152, 155], [152] |
|  |  | |  | + (100%) | + (73%) | + (87%) | [1] |
|  | STAT3; ERK 1/2 | | +; − |  |  | +; ↑ | [113]; [125] |
|  | HOXA9 | | + |  | ↑ |  | [32] |
|  | AKR1B10; Nrf2 | | –; + |  |  | ↑; ↓ | [92], [109] |
|  | Gα1/3; TSLP | | +; + |  |  | ↑; ↑ | [211], [172] |
|  | Wnt10a; iNOS | | +/– –; + |  |  | ↑; ↑ | [206]; [76] |
|  | NICD | | + |  | ↓ | ↑ | [97] |
| Dermal cells | cellularity | | ↓ | ↑ | ↑, – | ↑, + | [12, 20, 53, 74, 131, 139, 162, 171, 202, 203, 209], [105, 162] |
|  | proliferation | Ki67  PCNA | +  + | +  + | ↑, +  + | ↑, +  ↑ | [6, 16, 188], [188]  [7, 106] |
|  | apoptosis | TUNEL |  | + | ↑, + | ↑↑, ↓, ↑ | [5, 7, 164], [7, 164], [7] |
|  | in situ end labelling/acridine orange | | – |  |  | ↑ | [16] |
|  | Bcl-2, c-jun, c-fos, p53 | | +, +, +, – |  | +, +, +, – | +, +, +, – | [188] |
|  | p65 | | +/– – |  |  | ↑ | [121] |
|  | autophagy LC-3 | | + |  | ↓ |  | [171] |
|  | melanocortin-1 receptor, vit. D receptor | | +, + |  |  | ↓↓, + | [116], [65] |
|  | TGF-β  TGF-β1 | | –, +  −, + | ↑, +  + (60%) | ↑↑, +  ↑ (87%) | ↑  ↑ (87%), ↑ | [106, 166], [106, 142]  [2], [111]* |
|  | Hic-5, p-SMAD2/3, SMAD7 | | +, +, + |  |  | ↑, ↑, ↑ | [83] |
|  | KGF | | + |  |  | ↑ | [31] |
|  | PDGF, bFGF | | +, + | +, + | ↑, + |  | [142] |
|  | HDGF, HGF, c-Met | | +, +, + |  |  | ↑, ↑, ↑ | [151], [135], [135] |
|  | TNF-α, IFN-γ | | −, + | +, + | +, + |  | [142, 143] |
|  | IL-1α; IL-1β | | +; +, − | +; ↑, + | +; ↑, + |  | [142]; [142, 161] |
|  | IL-4, IL-6, IL-15 | | +, –, + | +, –, + | +, NA, ↑ | NA, ↑, NA | [143], [137], [35] |
|  | IGF-1 | | – | + | ↑ |  | [56] |
|  | CXCL1, CXCR2; CXCR1 | | –, –; + |  | –, – | ↑, ↑; ↑ | [144]; [210] |
|  | HSP70 | | + |  |  | ↑ | [106] |
|  | VEGF | | + | + |  | ↑ | [160, 179] |
|  | Ang 1, Ang 2, TIE-2 | | +, +, + | +, +, ↑ |  |  | [179] |
|  | prothrombin, thrombin, thrombomodulin | | –, –, – | ↑, ↑, ↑ |  |  | [18] |
|  | PAR-1 | | + | ↑ |  |  | [18] |

**Supplemental table 1.** Histopathology of normal skin and scars – continued

| **Location** | **Parameter** | **Nskin** | **Nscar** | **Hscar** | **Kscar** | **References** |
| --- | --- | --- | --- | --- | --- | --- |
| Dermal cells | FVIII, FXIIIa |  | NA, – | –, – | –, – | [3], [3] [94]‡ |
|  | CD34, P4H | +, +/– – | +, – | –, + | –, + | [3, 4, 24], [4] |
|  | CD34 / vimentin / Te7 | + |  |  |  | [140] |
|  | CD34 / S-100 |  | – / – | – / – | – / – | [94]‡ |
|  | CD34 | +/–, + | +/–, – |  | + (77%), ↓ | [20, 130], [54, 86, 141] |
|  | c-KIT | –, + | ↑ |  | + | [20, 72], [72] |
|  | galectin-7; pleiotrophin; leptin | −; +/– –; + | NA; NA; + | ↑; ↑; ↑ | NA; NA; ↑↑ | [41]; [209]; [167] |
|  | periostin | + |  | ↑ | ↑ | [120] |
|  | adiponectin, adiponectin receptor | +, + |  |  | ↑, ↓ | [117] |
|  | COX-1 | + | + | ↑ | +/– –, ↑ | [152, 155], [152] |
|  |  |  | – (100%) | +/– (50%) | – (93%) | [1] |
|  | COX-2 | + | + | + | ↑, + | [152, 155], [152] |
|  |  |  | + (30%) | +/– (73%) | + (87%) | [1] |
|  | STAT3; p-STAT3 (Tyr705), β-catenin | +; +, + |  |  | ↑; ↑, ↑ | [113]; [110] |
|  | ERK 1/2, ERK phosphorylation; Wnt-3a | −, +; + |  |  | ↑, ↑; + | [125], [125]; [108] |
|  | JAG-1, Notch-1/2 | +/– –, +/– – |  |  | ↑, ↑ | [183] |
|  | SDF1α, CXCR4, TSLP | +/– –, +, + |  |  | ↑, ↑, ↑ | [172] |
| Fibroblasts | cellular density | + | + | ↑ | ↑, + / ↑ | [88, 125, 128, 187, 192], [170] |
|  | AgNORs | + |  | ↑ | ↑ | [58] |
|  | CD34 / α-SMA / p16 | + / – / – | + / – / – | – / ++ / + | – / + / ++ | [114] |
|  | Ki67 | –, + | + | + | ↑, + | [26, 90, 114], [90, 114] |
|  | TUNEL; p53 | –; NA |  | ↑; NA | ↑; +/– | [5, 6]; [70] |
|  | caspase-1 | + |  |  | ↑ | [49] |
|  | caspase-2 | +/– – | +/– |  | ↑↑ | [7] |
|  | caspase-3 | +/– –, + | +, +/– | +, ↑ | ↑, ↑↑ | [6, 7, 49], [6, 49] |
|  | caspase-9 |  | + | + | ↑ | [7] |
|  | vim; HSP47; dermatopontin; fibronectin | +; +; +; + |  |  | +; ↑; ↓;↑ | [102], [139], [157], [111]* |
|  | collagen I; FSP-1 | +/–, +; – |  | ↑; ↑ |  | [90, 208], [90]; [200] |
|  | β-catenin; cathepsin | –; +/– – | –; ↑ |  | ↑; ↑↑ | [80, 163, 208]; [156] |
|  | TGF-β  TGFβRI, TGFβRII | +, –  +/– –, +/– – | +/– | ↑  ↑, ↑ | ↑ | [129, 163, 208], [163, 208]  [165] |
|  | SMAD 2/3/4, SMURF2 | +, + |  |  | ↑, ↑ | [29] |
|  | PDGF, PDGFRβ | +, + |  |  | ↑, ↓ | [129] |
|  | FGF-1, FGF-2, FGFR | +, +, + |  |  | +, ↑, ↓ | [129, 136] |
|  | EGF, EGFR | +, + |  |  | ↑, ↓ | [129] |
|  | HGF, c-Met, p-Met | +, +, + |  |  | ↑, ↑, ↑ | [90] |
|  | TNF-α; TNF-β | +; + |  |  | –, ↑; ↑ | [126, 129], [126]; [129] |
|  | IL-6, IL-6R, downstream targets | +, – |  |  | ↑, ↑ | [59, 129] |
|  | IL-18, IL-18Rα | +, + |  |  | ↑, ↑ | [49] |
|  | MCP-1, CCR2 | +, + |  | +, + | ↑, ↑ | [112] |
|  | integrins α1β1, α2β1  integrins α5β1, α5β3 | +/–, +/–  +/–, + |  |  | ↑, ↑  ↑, ↑ | [184]  [184], [129] |
|  | IGF-1, IGFBP5  IGF-1R | –, +  –, +/– – |  | + (30%) | –, ↑  ↑ (95%) | [129], [157]  [77, 146], [204] |
|  | VEGF, CTGF | +, + |  |  | ↑, ↑ | [129], [157] |
|  | PAI-1, uPA, uPAR | –, –, NA | ↑, ↑,+/– |  | ↑↑, ↓, ↑ | [191], [191], [104] |
|  | HLA-DR, HLA-DQ, IL-2R | –, –, – | –, –, – | ↑, –, – |  | [34] |
|  | MMP-1  MMP-2; MMP-3  MMP-9  TIMP-1; TIMP-2; TIMP-3 | +; +  +  +; NA; NA | +  ↑, +; NA  ↑, +  ↑, –; –; – |  | +  ↑; ↓  ↑, +  –; ↑; ↑ | [82]  [60, 82, 158],[82]; [157]  [60, 158],  [82]; [60, 82]; [82] |
|  | TSG-6, IαI polypeptides | +, + | +, + | +, + |  | [186] |
|  | Wnt5a; MIF | +/– –; + |  |  | ↑; ↑ | [80]; [68] |
|  | DNMT1; NICD | +/–; +/– – |  | ↑; ↑ | ↑; ↑↑ | [52]; [97] |
|  | HtrA1; Gα1/3 | +; + |  |  | ↑; ↑ | [199]; [207] |
|  | SFRP1, JAG1 | +, + |  |  | ↓, ↑ | [157] |
|  | RUNX2, caveolin-1 | –, + | +, + | +, NA | ↑, ↓ | [75] |
| Myofibroblasts | α-SMA | – | +/– – | ↑↑ | +/– – | [12] |
|  |  | – | – | ↑↑ | – (88%) | [45, 53] |
|  |  |  | – | ↑ | ↑ (60-81%) | [94]‡ [162] |
|  |  |  |  | ↑ (70%) | ↑ (45%) | [107] |
|  |  |  | +/– – | – | ↑ (33%) | [131, 172] |
|  |  | +, – | – | ↑ | – | [74, 130, 165, 184], [130, 165] |
|  |  | + |  |  | ↑, – | [105], [189] |
|  |  | – | – | ↑↑ | ↑ | [114] |
|  | PAR-1, PAR-2 |  |  | +, + | +, + | [123] |

**Supplemental table 1.** Histopathology of normal skin and scars – continued

| **Location** | **Parameter** | | **Nskin** | **Nscar** | **Hscar** | **Kscar** | **References** |
| --- | --- | --- | --- | --- | --- | --- | --- |
| Fibrocytes | identification by cell shape | |  | + | ↑ | ↓ | [192] |
|  | CD45RO / 25F9 / MRP8/9 | |  | +/– – |  | ↑ | [85] |
|  | CD45 / collagen I / CXCR4 | | + |  |  | ↑ | [172] |
| IHC profile | dependent on scar maturity (cellularity, nodules, α-SMA, immune cells) | |  |  | yes | no | [162]  [162] |
| ECM | connective tissue | |  |  | ↑ | ↑ | [53, 123] |
|  | nodules | |  |  | + | –, +/– – | [131], [12, 53] |
|  |  | |  |  | + | + | [16, 21, 78, 79, 107, 123, 162, 168] |
|  |  | |  |  | + (50%) | + (48%) | [107] |
|  |  | |  |  | + (100%) | + (58-69%) | [162, 170] |
|  |  | | – |  | + |  | [57] |
|  |  | | – | – | large (60%) | small (60%) | [114] |
|  | keloidal collagen | | – | – | – | + | [53, 78, 88, 131, 162, 168, 169, 186, 189] |
|  |  | |  |  | – | + (55%) | [107]^ |
|  |  | |  | + | +/– – | + | [95, 145] |
|  |  | | – | – | – (80%) | + (100%) | [114] |
|  | non-fibrotic PD | |  | − | + (80%)  − | + (40%)  − | [107]  [131] |
|  | tongue-like advancing edge | |  |  | – | + | [107, 131] |
|  | horizontal fibrous band in upper RD  whorled fibrous bundles in RD | |  | – | –  + | + (>93%)  – | [82, 107, 131]  [107, 184] |
|  | prominent fascia-like band | |  |  | – | + | [107] |
|  | collagen bundle thickness | | + | + | + | ↑ | [196] |
|  |  | | thin |  | thin |  | [74, 209] |
|  |  | | thick |  | thick | thick | [42, 103, 125, 180] |
|  | flexible collagen fibers | | + |  | – |  | [74, 209] |
|  | loose collagen fibers | | –, + |  | + | – | [42, 74, 103, 184, 203, 209] |
|  | dense collagen fibers | |  | + | + | + | [21, 42, 74, 103, 203, 209] |
|  | collagen orientation | |  | wavy | parallel | haphazard | [107, 131, 162, 170] |
|  |  | |  | parallel |  | disoriented | [20] |
|  |  | | random | parallel | parallel | parallel | [16, 21, 74, 196, 209] |
|  |  | | network |  | network − | network − | [74, 125, 209] |
|  | collagen crosslinking | | ↓ |  |  | ↑ | [38] |
|  | collagen type I | PD | + | + | + | + | [71] |
|  | RD | | + | ↑ | + | ↓, ↑ | [71] , [182] |
|  | overall | | + |  | + | +, ↑ | [17, 88, 90, 139, 147] , [139, 182] |
|  | collagen type III | PD | + | + | ↑ | ↓, ↑ | [71, 182], [182] |
|  | RD | | +/– | + | + | + | [71] |
|  | overall | | +, +/–, ↓ |  | ↑ | ↓, ↑, + | [17, 88, 139, 147], [139, 182], [38, 88, 147] |
|  | collagen I:III ratio | | ↑ |  |  | ↓ | [38] |
|  | COMP; collage XIV | | –; + |  |  | ↑ (67%); + | [84]; [48] |
|  | collagen IV, collagen VII, laminin | | +, +, + |  | +, +, + |  | [45] |
|  | fibronectin | | +/– –, + | ↑ | ↑ | ↑, +, ↑↑ | [98, 138, 173], [48, 138], [173] |
|  | fibrillin-1 | | + | ↓ | ↓, + | ↓, ↑ | [13, 45, 81], [45, 81] |
|  | fibromodulin | | + |  |  | ↓ | [74] |
|  | elastin | PD | +/– – | ↓ | ↓ | ↓ | [13, 103] |
|  | RD | | + | + | + | ↑ | [13, 103] |
|  | overall | | + | – | –, +/–, + | (50%), − | [94]‡ [81], [42, 45, 184], [81, 184, 189] |
|  | DANCE (required for elastic fibers) | | + |  |  | ↓ | [81] |
|  | thickness PD, thickness RD | | thin, thin |  | thin, thin |  | [13, 42, 103] |
|  | density PD, density RD | | ↓, ↑ |  | –, ↓ |  | [13, 42, 103] |
|  | hyaluronan | | + | + | ↓↓ | ↓ | [25] |
|  | hyaluronic acid | | +, +/– – | ↓  + | ↑ | ↓↓, ↑  ↓ PD, ↑ RD | [8, 81, 127, 198], [8, 81, 198]  [50, 186] |
|  | hyaluronan synthase, hyaluronidase | | +, + |  |  | ↑, ↑ | [175] |
|  | epidermis:dermis hyaluronic acid ratio | | ↓ | ↓ |  | + | [186] |
|  | glycosaminoglycans | | –, +, +/– – | + | ↑ | ↑ | [33, 50, 81], [33], [81] |
|  | dermatan sulphate | | + | + | ↑ |  | [50] |
|  | chondroitin sulphate | | +, +/– – | + | ↑ | ↑ | [50, 81], [81] |
|  | versican | | –, + | ↑ | ↑↑ | ↑ | [166, 198], [198] |
|  | biglycan | | +, –, +/– | ↑ | ↑↑ | ↑, +/– | [79, 166], [74, 185], [185] |
|  | decorin | | + | ↑↑ | ↓ | ↓, + | [33, 74, 79, 166], [79, 185] |
|  | lumican | | + |  | ↓ |  | [74] |
|  | tenascin; periostin | | +, + |  | ↑, ↑ | ↑, ↑ | [45, 48], [120, 214] |
|  | TGF-β1 | | − |  | ↑ |  | [57] |
|  | uPAR | |  | – |  | ↑ | [104] |

**Supplemental table 1.** Histopathology of normal skin and scars – continued

| **Location** | **Parameter** | | **Nskin** | **Nscar** | **Hscar** | **Kscar** | **References** |
| --- | --- | --- | --- | --- | --- | --- | --- |
| Endothelial cells | vascular density | | + | + | ↑ | ↑ | [12, 20, 53, 74, 123, 149, 183, 187] |
|  |  | | + | + | ↓ | ↓, ↓↓ | [23, 101, 189, 193], [193] |
|  |  | |  | ↑ | ↑ |  | [202, 203] |
|  |  | | + | + |  | ↑ P, ↓ C | [190] |
|  | microvascular density | | + | +, ↑ | ↑ | ↑, ↓ | [14, 194], [30, 100, 213] |
|  | vascular lumen | | + | ↑ | ↓ | ↓↓ | [101, 193, 194] |
|  | vertical vessel orientation | |  | + | + | − | [95, 107, 130, 131] |
|  | vascular orientation | |  | horizontal |  | aggregate  below epi | [131] |
|  | CD31 / vimentin (EndoMT) | | − / − |  |  | ↑ / ↑ | [108] |
|  | endothelin-1 | | + | + | ↑ | ↑ | [99] |
|  | thrombomodulin, PAR-1 | | +, + | ↑, ↑ |  |  | [18] |
|  | HIF-1α | | − | + |  | ↑ P, ↑↑ C | [190, 213] |
|  | VEGF | | − | +, ↑ |  | +, ↑↑, − | [129, 190, 194], [100, 190], [129] |
|  | Ang 1 | | +/– – |  | ↓ |  | [194] |
|  | Ang 2 | | +/– – |  | ↑ |  | [194] |
|  | TGF-β | | − |  |  | − | [129] |
|  | TGFβRI, TGFβRII | | +, + |  | +, + |  | [165] |
|  | PDGF, PDGFRβ | | +, + |  |  | ↓, ↓ | [129] |
|  | FGF-1, FGF-2, FGFR | | +, +, + |  |  | –, –, + | [129] |
|  | EGF, EGFR | | +, + |  |  | +, + | [129] |
|  | TNF-α, TNF-β, | | –, – |  |  | –, – | [129] |
|  | TNFR1 | | + | – | ↑ |  | [161] |
|  | IL-6, IL-6R | | –, + |  |  | –, ↓ | [129] |
|  | IGF-1, IGF-1R | | +, + |  |  | +, + | [129], [204] |
|  | αVβ3 | | + |  |  | + | [129] |
|  | HLA-DR, HLA-DQ  IL-2R | | +, +  – | +, +  – | +, +  – |  | [34]  [34] |
|  | MMP-9 | | + | ↑ |  |  | [60] |
|  | NICD | | + |  | + | ↑ | [97] |
| Nerve cells | nerve fibers | | + | –, + | ↓ |  | [11, 18], [11] |
|  | neuropeptides | | + |  | ↑ |  | [103] |
|  | neuropeptide-containing nerve fibers | | + | +, – | +, ↑ |  | [11, 47], [47] |
|  | nerve density (PGP 9.5) | | + |  |  | ↓ | [159] |
|  | nerve density (α1-AR / PGP 9.5) | | + |  |  | ↑ | [51] |
|  | nerve density (S100) | | + |  |  | ↑ | [73] |
|  | dimensions | | thick, short |  |  | thin, long | [73] |
|  | depth of superficial fibers | | ↓ |  |  | ↑ | [73] |
| Immune cells | infiltrate | | –, + | –, ↑, + | ↑, ↑↑ | ↑, few | [12, 34, 36, 37, 87, 117, 168, 170, 187], [16, 34, 36], [36] |
|  | infiltrate expression TNF-α, TNF-β | | +, + | ↑, ↑ | +, ↑ |  | [36] |
|  | KALT (distinctive lymphoid aggregates) | | − | − |  | (15%), + | [19], [62] |
|  | embryonic stem cell markers | |  |  |  | + | [62] |
|  | mononuclear cells | POMC | − |  |  | ↑ | [177] |
|  | macrophages | CD68 | + | −, + | −, + | −, ↑ | [3, 130], [28, 87, 143, 168] |
|  | CD11c | | + | + | ↑ |  | [36] |
|  | HAM56 | | + | + | + |  | [161] |
|  | M1 (CD68) | | + | + |  | ↑ | [19] |
|  | M1 (IL-12, iNOS) | | + |  |  | ↑ | [89] |
|  | M2 (CD163) | | + | + |  | ↑ | [19] |
|  | M2 (IL-10, TGF-β) | | + |  |  | ↑ | [89] |
|  | Lymphocytes | |  | + | + |  | [161] |
|  | B-lymphocytes | | −, few | − | − | −, ↑, few | [19, 28, 36], [19, 87, 88], [168] |
|  | T-lymphocytes | CD3, CD4 | +, +/– |  |  | ↑, ↑ | [87, 168], [137] |
|  | CD3 / CD4 / CD8 | | + | + | ↑ | ↑ | [19, 28, 36] |
|  | CD4:CD8 ratio | | + |  | ↑ | ↑ | [19, 28, 36] |
|  | CD4 / FOXP3 Tregs | | + |  |  | + | [137] |
|  | Activated (CD3 / IL-2R / HLA-DR)  TNF-β | | +  + | ↑, +  ↑ | ↑↑, ↑  ↑↑ |  | [36], [37]  [36] |
|  | natural killer (CD56) | | + | + | ↑ |  | [36] |
|  | CD3 / CD45RO / CD4 / HLA-DR / LFA-1 | |  |  | + | ↑ | [162] |
|  | mast cells | tryptase | + | +, ↑ | + | ↑ | [10, 14, 42, 72, 122, 143], [72] |
|  | chymase | | + | ↓ |  |  | [14, 19, 72] |
|  | overall | | + | +, − | +, ↑, − | +, ↓, ↑ | [16, 22, 71, 103, 131, 205], [42, 71, 168] [94]‡ , [94]‡ [131, 168, 170] |

**Supplemental table 1.** Histopathology of normal skin and scars – continued

| **Location** | **Parameter** | | **Nskin** | **Nscar** | **Hscar** | **Kscar** | **References** |
| --- | --- | --- | --- | --- | --- | --- | --- |
| Immune cells |  | Langerhans cells | + | + | +  ↑ | +  ↑ | [19, 34, 36, 40, 46, 87, 143, 161] [34, 36, 46, 87, 143] |
|  | CD1a / CD36 / HLA-DR / ICAM-1 DC | |  |  | + | ↑ | [162] |
|  | HLA-DR DC  CD1a DC | | +  + |  | ↑  ↑ | ↑  ↑ | [40]  [40] |
|  | FXIIIa DC | |  | ↑ | ↑ | ↑↑ | [150] |
|  | NICD | | + |  | ↑ | ↑↑ | [97] |
|  | IgA, IgM, C3, C1q deposits | | − |  |  | ↑ | [87] |
| Other | mTOR activation | |  | + | ↑ | ↑ | [15] |
|  | GDF-9 | | – |  | – | ↑ | [55] |
|  | S100, p63, desmin, LMWK, P KER | |  | − |  |  | [130] |
|  | FGF-2, LTBP-2 | | +/– – | +, + | ↑, ↑ | ↑, ↑ | [174] |
|  | neuregulin-1, ErbB2, ErbB3, ErbB4 | | +, +,+, + |  |  | ↑, ↑, ↓, ↓ | [93] |

**Supplemental table 1.** Histopathology of normal skin and scars, parameter expression of scars listed as compared to normal skin. Table contains all publications in which normal skin or mature normotrophic, hypertrophic and keloid scars were subjected to simple light microscopy histological, immunohistochemical or immunofluorescence analysis (flow cytometry, electron microscopy, or gene expression studies were not included). Non-conflicting results were combined in a single row as much as possible. If there is no value listed, the experimental group was not included in the study’s analysis and there was no value available. The dermal cell population constitutes the entire spectrum of cell types in the dermis; this category was included to reflect studies in which findings were not specified per dermal cell type. Legend; +: present, normal expression or values; ↑: increased; −: absent; ↓: decreased; + / ↑: increased in some samples but not all; ≈: similar to normal skin and/or normal scar; NA: not applicable, when value is not available/known; ^: hypertrophic scars were said to have no keloidal collagen at all, but both 0 and small fragments of keloidal collagen were scored as negative. Abbreviations in alphabetical order; α-MSH: alpha melanocyte stimulating hormone; α-SMA: alpha smooth muscle actin; α1-AR: alpha 1 adrenergic receptor; AgNORs: silver-stained nucleolar organiser regions (marker for cellular activity and proliferation); AKR1B10: aldo-keto reductase (retinoic acid metabolism); Ang: angiopoietin; Bcl-2: B-cell lymphoma 2; bFGF: basic fibroblast growth factor; BM: basement membrane; C3/C1q: complement C3 or C1q; C: central region of keloid; COX: cyclo-oxygenase; CGRP: calcitonin gene-related peptide (neuropeptide); c-KIT: receptor tyrosine kinase, also known as CD117 (embryonic stem cell marker); c-Met: hepatocyte growth factor receptor; COMP: cartilage oligomeric matrix protein (accelerates collagen I fibril assembly); CTGF: connective tissue growth factor; CXCR: C-X-C chemokine receptor; CXCR4: C-X-C chemokine receptor type 4 (SDF1α receptor); DC: dendritic cell; DNMT1: DNA methyltransferase 1 (catalyses DNA methylation); ECM: extracellular matrix; EGFR: epidermal growth factor receptor; EndoMT: endothelial-mesenchymal transition; EMT: epithelial-mesenchymal transition; ErbB2/3/4: downstream signalling of neuregulin-1; ERK: extracellular signal–regulated kinases (pathway activated by periostin to promote angiogenesis); FGF: fibroblast growth factor; FGFR: fibroblast growth factor receptor; FSP1: fibroblast-specific protein 1; FVIII: factor VIIII; FXIIIa: factor XIIIa; Gα1/3: α-subunits of the G proteins (involved in KGF signalling); GDF-9: growth differentiation factor 9; HDGF: hepatoma-derived growth factor; HGF: hepatocyte growth factor; Hic-5: hydrogen peroxide-inducible clone-5 (TGFβ inducible transcriptional regulator); HIF-1α: hypoxia-inducible factor 1 alpha; HLA: human leukocyte antigen; HMB-45: stains melanoma cells; HOXA9: homeobox A9; HSP: heat shock protein; HtrA1: a serine protease; IGF-1: insulin-like growth factor 1; IFN-γ: interferon gamma; IgA/IgM: immunoglobulin A or M; IGF-1R: insulin-like growth factor 1 receptor; IGFBP: insulin-like growth factor binding protein; IL: interleukin; IL-6R: interleukin 6 receptor; IL-18Rα: interleukin 18 receptor alpha; IL-31RA: interleukin 31 receptor A (its ligand is associated with pruritus); iNOS: inducible nitric oxide synthase; JAG1: jagged 1 protein (ligand); JUP: junctional plakoglobin (desmosome component); K: keratin; KALT: keloid-associated lymphoid tissue (similar to mucosa-associated lymphoid tissue); KGF: keratinocyte growth factor; LC-3: microtubule-associated protein 1A/1B-light chain 3 (autophagy); LMWK: low molecular weight keratin; LTBP-2: latent-transforming growth factor beta-binding protein 2; M2: alternatively activated, profibrotic macrophages; MIF: macrophage migration inhibition factor; MMP: matrix metalloproteinase; myofibs: myofibroblasts; mTOR: mammalian target of rapamycin; NICD: Notch intracellular domain (involved in cell fate determination, modulates e.g. proliferation, apoptosis, migration); Notch: JAG receptor; Nrf2: nuclear factor erythroid 2-related factor 2 (defense against oxidative stress, involved in apoptosis); OSMR: oncostatin-M specific receptor (part of IL-31 signalling); P: periphery of keloid; P4H: proline-4-hydroxylase (marker for active collagen synthesis); PAI-2: plasminogen activator inhibitor 2; PAR: protease-activated receptor; PD: papillary dermis; PDGF: platelet-derived growth factor; PDGFRβ: platelet-derived growth factor receptor beta; PGP9.5: protein gene product 9.5 (neuronal marker); P KER: cytokeratins (keratins predominantly of molecular weight 56 and 69 kDa); PKP1: plakophilin 1 (desmosome component); POMC: proopiomelanocortin; RD: reticular dermis; RUNX2: Runt-related transcription factor 2 (involved in osteogenesis, chondrogenesis); SB: stratum basale of epidermis; SFRP1: secreted frizzled related protein 1; SKALP: skin-derived antileukoproteinase; SMAD: mothers against decapentaplegic homolog 1 (Drosophila), major effectors of TGF-β signalling; SMURF2: SMAD specific E3 ubiquitin protein ligase 2 (SMAD inhibitory effect); SPARC: secreted protein acidic and rich in cysteine (thought to control collagen I synthesis together with stratifin); SPRR2: small proline-rich protein 2; STAT3: signal transducer and activator of transcription 3; SDF1α: stromal cell-derived factor alpha; Te7: human thymic fibroblasts antibody; TGF-β: transforming growth factor beta; TGFβR: transforming growth factor beta receptor; TIE-2: tyrosine-protein kinase receptor 2 (angiopoietin receptor) TIMP: tissue inhibitor of metalloproteinase; TNF: tumour necrosis factor; Tregs: regulatory T-lymphocytes; TSG-6: (tumour necrosis factor) TNF-inducible gene 6; TSLP: thymic stromal lymphopoietin (potential collagen synthesis initiator) uPAR: urokinase-type plasminogen activator receptor; VEGF: vascular endothelial growth factor; vim: vimentin; Wnt3a/5a/10a: Wnt family member 3a/5a/10a. N.B. additional information on parameters listed in abbreviations all derived from cited literature in table.

**References**

1. Abdou AG, Maraee AH, Saif HFA-E (2014) Immunohistochemical evaluation of COX-1 and COX-2 expression in keloid and hypertrophic scar. Am J Dermatopathol 36:311–317

2. Abdou AG, Maraee AH, Al-Bara AM, Diab WM (2011) Immunohistochemical expression of TGF-β1 in keloids and hypertrophic scars. Am J Dermatopathol 33:84–91

3. Aiba S, Tabata N, Ishii H, et al (1992) Dermatofibrosarcoma protuberans is a unique fibrohistiocytic tumour expressing CD34. Br J Dermatol 127:79–84

4. Aiba S, Tagami H (1997) Inverse correlation between CD34 expression and proline-4 hydroxyase immunoreactivity on spindle cells noted in hypertrophic scars and keloids. J Cutan Pathol 24:65–69

5. Akasaka Y, Fujita K, Ishikawa Y, et al (2001) Detection of apoptosis in keloids and a comparative study on apoptosis between keloids, hypertrophic scars, normal healed flat scars, and dermatofibroma. Wound Repair Regen 9:501–506

6. Akasaka Y, Ishikawa Y, Ichiro O, et al (2000) Enhanced expression of caspase-3 in hypertrophic scars and keloid: induction of caspase-3 and apoptosis in keloid fibroblasts in vitro. Lab Investig 80:345–357

7. Akasaka Y, Ito K, Fujita K, et al (2005) Activated caspase expression and apoptosis increase in keloids: cytochrome c release and caspase-9 activation during the apoptosis of keloid fibroblast lines. Wound Repair Regen 13:373–382

8. Alaish SM, Yager DR, Diegelmann RF, Cohen IK (1995) Hyaluronic acid metabolism in keloid fibroblasts. J Pediatr Surg 30:949–952

9. Alessio M, Gruarin P, Castagnoli C, et al (1998) Primary ex vivo culture of keratinocytes isolated from hypertrophic scars as a means of biochemical characterization of CD36. Int J Clin Lab Res 28:47–54

10. Algermissen B, Hermes B, Feldmann-Boeddeker I, et al (1999) Mast cell chymase and tryptase during tissue turnover: analysis on in vitro mitogenesis of fibroblasts and keratinocytes and alterations in cutaneous scars. Exp Dermatol 8:193–198

11. Altun V, Hakvoort TE, Van Zuijlen PPM, et al (2001) Nerve outgrowth and neuropeptide expression during the remodeling of human burn wound scars: a 7-month follow-up study of 22 patients. Burns 27:717–722

12. Amadeu T, Braune A, Mandarim-de-Lacerda C, et al (2003) Vascularization pattern in hypertrophic scars and keloids: a stereological analysis. Pathol Res Pract 199:469–473

13. Amadeu TP, Braune AS, Porto LC, et al (2004) Fibrillin-1 and elastin are differentially expressed in hypertrophic scars and keloids. Wound Repair Regen 12:169–174

14. Ammendola M, Zuccalà V, Patruno R, et al (2013) Tryptase-positive mast cells and angiogenesis in keloids: a new possible post-surgical target for prevention. Updates Surg 65:53–57

15. Andreoli A, Ruf MT, Itin P, et al (2015) Phosphorylation of the ribosomal protein S6, a marker of mTOR (mammalian target of rapamycin) pathway activation, is strongly increased in hypertrophic scars and keloids. Br J Dermatol 172:1415–1417

16. Appleton I, Brown NJ, Willoughby DA (1996) Apoptosis, necrosis, and proliferation: possible implications in the etiology of keloids. Am J Pathol 149:1441–1447

17. Arbi S, Eksteen EC, Oberholzer HM, et al (2015) Premature collagen fibril formation, fibroblast-mast cell interactions and mast cell-mediated phagocytosis of collagen in keloids. Ultrastruct Pathol 39:95–103

18. Artuc M, Hermes B, Algermissen B, Henz BM (2006) Expression of prothrombin, thrombin and its receptors in human scars. Exp Dermatol 15:523–529

19. Bagabir R, Byers RJ, Chaudhry IH, et al (2012) Site-specific immunophenotyping of keloid disease demonstrates immune upregulation and the presence of lymphoid aggregates. Br J Dermatol 167:1053–1066

20. Bakry OA, Samaka RM, Basha MA, et al (2014) Hematopoietic stem cells: do they have a role in keloid pathogenesis? Ultrastruct Pathol 38:55–65

21. Beausang E, Floyd H, Dunn KW, et al (1998) A new quantitative scale for clinical scar assessment. Plast. Reconstr. Surg. 102:1954–1961

22. Beer TW, Baldwin H, West L, et al (1998) Mast cells in pathological and surgical scars. Br J Opthalmology 82:691–694

23. Beer TW, Baldwin H, Goddard JR, et al (1998) Angiogenesis in pathological and surgical scars. Hum Pathol 29:1273–1278

24. Benias PC, Wells RG, Sackey-Aboagye B, et al (2018) Structure and distribution of an unrecognized interstitium in human tissues. Sci Rep 8:1–8

25. Bertheim U, Hellström S (1994) The distribution of hyaluronan in human skin and mature, hypertrophic and keloid scars. Br J Plast Surg 47:483–489

26. Betz P, Nerlich A, Wilske J, et al (1993) The time-dependent localization of Ki67 antigen-positive cells in human skin wounds. Int J Leg Med 106:35–40

27. Bloor BK, Tidman N, Leigh IM, et al (2003) Expression of keratin K2e in cutaneous and oral lesions: association with keratinocyte activation, proliferation, and keratinization. Am J Pathol 162:963–975

28. Boyce DE, Ciampolini J, Ruge F, et al (2001) Inflammatory cell subpopulations in keloid scars. Br J Plast Surg 54:511–516

29. Bran GM, Sommer UJ, Goessler UR, et al (2010) TGF-β1 antisense impacts the SMAD signalling system in fibroblasts from keloid scars. Anticancer Res 3464:3459–3463

30. Bux S, Madaree A (2010) Keloids show regional distribution of proliferative and degenerate connective tissue elements. Cells Tissues Organs 191:213–234

31. Canady J, Arndt S, Karrer S, Bosserhoff AK (2013) Increased KGF expression promotes fibroblast activation in a double paracrine manner resulting in cutaneous fibrosis. J Invest Dermatol 133:647–657

32. Cao PF, Xu Y Bin, Tang JM, et al (2014) HOXA9 regulates angiogenesis in human hypertrophic scars: Induction of VEGF secretion by epidermal stem cells. Int J Clin Exp Pathol 7:2998–3007

33. Carrino DA, Mesiano S, Barker NM, et al (2012) Proteoglycans of uterine fibroids and keloid scars: similarity in their proteoglycan composition. Biochem J 443:361–368

34. Castagnoli C, Stella M, Magliacani G, et al (1990) Anomalous expression of HLA class II molecules on keratinocytes and fibroblasts in hypertrophic scars consequent to thermal injury. Clin Exp Immunol 82:350–354

35. Castagnoli C, Trombotto C, Ariotti S, et al (1999) Expression and role of IL-15 in post-burn hypertrophic scars. J Invest Dermatol 113:238–245

36. Castagnoli C, Stella M, Berthod C, et al (1993) TNF production and hypertrophic scarring. Cell. Immunol. 147:51–63

37. Castagnoli C, Trombotto C, Ondei S, et al (1997) Characterization of T-cell subsets infiltrating post-burn hypertrophic scar tissues. Burns 23:565–572

38. Di Cesare PE, Cheung DT, Perelman N, et al (1990) Alteration of collagen composition and cross-Linking in keloid tissues. Matrix 10:172–178

39. Chavez-Muñoz C, Hartwell R, Jalili RB, et al (2012) SPARC/SFN interaction, suppresses type I collagen in dermal fibroblasts. J Cell Biochem 113:2622–2632

40. Chen D, Wang Q, Bao W, et al (2003) Role of HLA-DR and CD1a molecules in pathogenesis of hypertrophic scarring and keloids. Chin Med J (Engl) 116:314–315

41. Cho S Bin, Kim JS, Zheng Z, et al (2013) Decreased tissue and serum expression of galectin-7 in patients with hypertrophic scars. Acta Derm Venereol 93:669–673

42. Choi YH, Kim KM, Kim HO, et al (2013) Clinical and histological correlation in post-burn hypertrophic scar for pain and itching sensation. Ann Dermatol 25:428–433

43. Chong Y, Park TH, Seo SW, Chang CH (2015) Histomorphometric analysis of collagen architecture of auricular keloids in an Asian population. Dermatologic Surg 41:415–422

44. Chua AWC, Ma D, Gan SU, et al (2011) The role of R-spondin2 in keratinocyte proliferation and epidermal thickening in keloid scarring. J Invest Dermatol 131:644–654

45. Costa AMA, Peyrol S, Pôrto LC, et al (1999) Mechanical forces induce scar remodeling: study in non-pressure-treated versus pressure-treated hypertrophic scars. Am J Pathol 155:1671–1679

46. Cracco C, Stella M, Teich Alasia S, Filogamo G (1992) Comparative study of Langerhans cells in normal and pathological human scars. II. Hypertrophic scars*. Eur J Histochem 36:53–65

47. Crowe R, Parkhouse N, McGrouther D, Burnstock G (1994) Neuropeptide‐containing nerves in painful hypertrophic human scar tissue. Br J Dermatol 130:444–452

48. Dalkowski A, Schuppan D, Orfanos CE, Zouboulis CC (1999) Increased expression of tenascin C by keloids in vivo and in vitro. Br J Dermatol 141:50–56

49. Do D V., Ong CT, Khoo YT, et al (2012) Interleukin-18 system plays an important role in keloid pathogenesis via epithelial-mesenchymal interactions. Br J Dermatol 166:1275–1288

50. Donoff RB, Swann DA, Schweidt SH (1984) Glycosaminoglycans of normal and hypertrophic human scar. Exp Mol Pathol 40:13–20

51. Drummond PD, Dawson LF, Wood FM, Fear MW (2017) Up-regulation of α1-adrenoceptors in burn and keloid scars. Burns 44:582–588

52. E Y, Qipa Z, Hengshu Z (2014) The expression of DNMT1 in pathologic scar fibroblasts and the effect of 5-aza-2-deoxycytidine on cytokines of pathologic scar fibroblasts. Wounds 26:139–46

53. Ehrlich HP, Desmoulière A, Diegelmann RF, et al (1994) Morphological and immunochemical differences between keloid and hypertrophic scar. Am J Pathol 145:105–113

54. Erdag G, Qureshi HS, Patterson JW, Wick MR (2008) CD34-positive dendritic cells disappear from scars but are increased in pericicatricial tissue. J Cutan Pathol 35:752–756

55. Gao Z, Wu X, Song N, et al (2010) Differential expression of growth differentiation factor-9 in keloids. Burns 36:1289–1295

56. Ghahary A, Shen YJ, Wang R, et al (1998) Expression and localization of insulin-like growth factor-1 in normal and post-burn hypertrophic scar tissue in human. Mol Cell Biochem 183:1–9

57. Ghahary A, Shen YS, Scott PG, Tredget EE (1995) Immunolocalization of TGF-β1 in human hypertrophic scar and normal dermal tissues. Cytokine 7:184–190

58. Ghazizadeh M, Miyata N, Sasaki Y, et al (1997) Silver-stained nucleolar organizer regions in hypertrophic and keloid scars. Am J Dermatopathol 19:468–472

59. Ghazizadeh M, Tosa M, Shimizu H, et al (2007) Functional implications of the IL-6 signaling pathway in keloid pathogenesis. J Invest Dermatol 127:98–105

60. Gillard JA, Reed MWR, Buttle D, Cross SS (204AD) Matrix metalloproteinase activity and immunohistochemical profile of matrix metalloproteinase- 2 and -9 and tissue inhibitor of metalloproteinase-1 during human dermal wound healing. Wound Repair Regen 12:295–304

61. Gira AK, Brown LF, Washington C V., et al (2004) Keloids demonstrate high-level epidermal expression of vascular endothelial growth factor. J Am Acad Dermatol 50:850–853

62. Grant C, Chudakova DA, Itinteang T, et al (2016) Expression of embryonic stem cell markers in keloid-associated lymphoid tissue. J Clin Pathol 69:643–646

63. Hahn JM, Glaser K, McFarland KL, et al (2013) Keloid-derived keratinocytes exhibit an abnormal gene expression profile consistent with a distinct causal role in keloid pathology. Wound Repair Regen 21:530–544

64. Hahn JM, Mcfarland KL, Combs KA, Supp DM (2016) Partial epithelial-mesenchymal transition in keloid scars: regulation of keloid keratinocyte gene expression by transforming growth factor-β1. Burn Trauma 4:1–17

65. Hahn JM, Supp DM (2017) Abnormal expression of the vitamin D receptor in keloid scars. Burns 43:1506–1515

66. Hakvoort TE, Altun V, Ramrattan RS, et al (1999) Epidermal participation in post-burn hypertrophic scar development. Virchows Arch 434:221–226

67. Hakvoort TE, Altun V, Zuijlen PPM, et al (2000) Transforming growth factor-β1, -β2, -β3, basic fibroblast growth factor and vascular endothelial growth factor expression in keratinocytes of burn scars. Eur Cytokine Netw 11:233–239

68. Hayashi T, Nishihira J, Koyama Y, et al (2006) Decreased prostaglandin E2 production by inflammatory cytokine and lower expression of EP2 receptor result in increased collagen synthesis in keloid fibroblasts. J Invest Dermatol 126:990–997

69. He T, Bai X, Yang L, et al (2015) Loureirin B Inhibits Hypertrophic Scar Formation via Inhibition of the TGF- β 1-ERK/JNK Pathway. Cell Physiol Biochem 37:666–676

70. Heitzer E, Seidl H, Bambach I, et al (2012) Infrequent p53 gene mutation but UV gradient-like p53 protein positivity in keloids. Exp Dermatol 21:277–280

71. Hellström M, Hellström S, Engström-Laurent A, Bertheim U (2014) The structure of the basement membrane zone differs between keloids, hypertrophic scars and normal skin: a possible background to an impaired function. J Plast Reconstr Aesthetic Surg 67:1564–1572

72. Hermes B, Feldmann-Böddeker I, Welker P, et al (2000) Altered expression of mast cell chymase and tryptase and of c-Kit in human cutaneous scar tissue. J Invest Dermatol 114:51–55

73. Hochman B, Nahas FX, Sobral CS, et al (2008) Nerve fibres: a possible role in keloid pathogenesis. Br J Dermatol 158:624–657

74. Honardoust D, Varkey M, Hori K, et al (2011) Small leucine-rich proteoglycans, decorin and fibromodulin, are reduced in postburn hypertrophic scar. Wound Repair Regen 19:368–378

75. Hsu C, Lin H, Harn HI, et al (2018) Caveolin-1 controls hyperresponsiveness to mechanical stimuli and activation in keloid fibroblasts. J Invest Dermatol 138:208–218

76. Hsu YC, Hsiao M, Wang LF, et al (2006) Nitric oxide produced by iNOS is associated with collagen synthesis in keloid scar formation. Nitric Oxide - Biol Chem 14:327–334

77. Hu Z-C, Tang B, Guo D, et al (2014) Expression of insulin-like growth factor-1 receptor in keloid and hypertrophic scar. Clin Exp Dermatol 39:822–828

78. Huang C, Akaishi S, Hyakusoku H, Ogawa R (2014) Are keloid and hypertrophic scar different forms of the same disorder? A fibroproliferative skin disorder hypothesis based on keloid findings. Int Wound J 11:517–522

79. Hunzelmann N, Anders S, Sollberg S, et al (1996) Co-ordinate induction of collagen type I and biglycan expression in keloids. Br J Dermatol 135:394–399

80. Igota S, Tosa M, Murakami M, et al (2013) Identification and characterization of Wnt signaling pathway in keloid pathogenesis. Int J Med Sci 10:344–354

81. Ikeda M, Naitoh M, Kubota H, et al (2009) Elastic fiber assembly is disrupted by excessive accumulation of chondroitin sulfate in the human dermal fibrotic disease, keloid. Biochem Biophys Res Commun 390:1221–1228

82. Imaizumi R, Akasaka Y, Inomata N, et al (2009) Promoted activation of matrix metalloproteinase (MMP)-2 in keloid fibroblasts and increased expression of MMP-2 in collagen bundle regions: Implications for mechanisms of keloid progression. Histopathology 54:722–730

83. Inui S, Shono F, Noguchi F, et al (2010) In vitro and in vivo evidence of pathogenic roles of Hic-5/ARA55 in keloids through SMAD pathway and profibrotic transcription. J Dermatol Sci 58:152–164

84. Inui S, Shono F, Nakajima T, et al (2011) Identification and characterization of cartilage oligomeric matrix protein as a novel pathogenic factor in keloids. Am J Pathol 179:1951–1960

85. Iqbal SA, Sidgwick GP, Bayat A (2012) Identification of fibrocytes from mesenchymal stem cells in keloid tissue: A potential source of abnormal fibroblasts in keloid scarring. Arch Dermatol Res 304:665–671

86. Iqbal SA, Syed F, McGrouther DA, et al (2010) Differential distribution of haematopoietic and nonhaematopoietic progenitor cells in intralesional and extralesional keloid: do keloid scars provide a niche for nonhaematopoietic mesenchymal stem cells? Br J Dermatol 162:1377–1383

87. Jiao H, Fan J, Cai J, et al (2015) Analysis of characteristics similar to autoimmune disease in keloid patients. Aesthetic Plast Surg 39:818–825

88. Jiao H, Zhang T, Fan J, Xiao R (2017) The superficial dermis may initiate keloid formation: histological analysis of the keloid dermis at different depths. Front Physiol 8:1–9

89. Jin Q, Gui L, Niu F, et al (2018) Macrophages in keloid are potent at promoting the differentiation and function of regulatory T-cells. Exp Cell Res 362:472–476

90. Jin Z (2014) Increased c-Met phosphorylation is related to keloid pathogenesis: implications for the biological behaviour of keloid fibroblasts. Pathology 46:25–31

91. Jumper N, Paus R, Bayat A (2015) Functional histopathology of keloid disease. Histol Histopathol 30:1033–1057

92. Jumper N, Hodgkinson T, Arscott G, et al (2016) The aldo-keto reductase AKR1B10 is up-regulated in keloid epidermis, implicating retinoic acid pathway dysregulation in the pathogenesis of keloid disease. J Invest Dermatol 136:1500–1512

93. Jumper N, Hodgkinson T, Paus R, Bayat A (2017) A role for Neuregulin-1 in promoting keloid fibroblast migration. Acta Derm Venereol 97:675–684

94. Kamath NV, Ormsby A, Bergfeld WF, House NS (2002) A light microscopic and immunohistochemical evaluation of scars. J Cutan Pathol 29:27–32

95. Kaneishi NK, Cockerell CJ (1998) Histologic differentiation of desmoplastic melanoma from cicatrices. Am J Dermatopathol 20:128–134

96. Khoo YT, Ong CT, Mukhopadhyay A, et al (2006) Upregulation of secretory connective tissue growth factor (CTGF) in keratinocyte-fibroblast coculture contributes to keloid pathogenesis. J Cell Physiol 208:336–343

97. Kim JE, Lee JH, Jeong KH, et al (2014) Notch intracellular domain expression in various skin fibroproliferative diseases. Ann Dermatol 26:332–337

98. Kischer CW, Hendrix MJC (1983) Fibronectin (FN) in hypertrophic scars and keloids. Cell Tissue Res 231:29–37

99. Kiya K, Kubo T, Kawai K, et al (2016) Endothelial cell-­derived endothelin-1 is involved in abnormal scar formation by dermal fibroblasts through RhoA/Rho-kinase pathway. Exp Dermatol 26:705–712

100. Kumar I, Staton CA, Cross SS, et al (2009) Angiogenesis, vascular endothelial growth factor and its receptors in human surgical wounds. Br J Surg 96:1484–1491

101. Kurokawa N, Ueda K, Tsuji M (2010) Study of microvascular structure in keloid and hypertrophic scars: density of microvessels and the efficacy of three-dimensional vascular imaging. J Plast Surg Hand Surg 44:272–277

102. Kuwahara H, Tosa M, Murakami M, et al (2016) Examination of epithelial mesenchymal transition in keloid tissues and possibility of keloid therapy target. Plast Reconstr Surg Glob Open 4:1–7

103. Kwak IS, Choi YH, Jang YC, Lee YK (2014) Immunohistochemical analysis of neuropeptides (protein gene product 9.5, substance P and calcitonin gene-related peptide) in hypertrophic burn scar with pain and itching. Burns 40:1661–1667

104. Leake D, Doerr TD, Scott G (2003) Expression of urokinase-type plasminogen activator and its receptor in keloids. Arch Otolaryngol – Head Neck Surg 129:1334–1338

105. Lee CH, Hong CH, Chen YT, et al (2012) TGF-beta1 increases cell rigidity by enhancing expression of smooth muscle actin: keloid-derived fibroblasts as a model for cellular mechanics. J Dermatol Sci 67:173–180

106. Lee JH, Shin JU, Jung I, et al (2013) Proteomic profiling reveals upregulated protein expression of Hsp70 in keloids. Biomed Res Int 2013:621538

107. Lee JYY, Yang CC, Chao SC, Wong TW (2004) Histopathological differential diagnosis of keloid and hypertrophic scar. Am J Dermatopathol 26:379–384

108. Lee WJ, Park JH, Shin JU, et al (2015) Endothelial-to-mesenchymal transition induced by Wnt3a in keloid pathogenesis. Wound Repair Regen 23:435–442

109. Lee YJ, Kwon SB, Kim CH, et al (2015) Oxidative damage and nuclear factor erythroid 2-related factor 2 protein expression in normal skin and keloid tissue. Ann Dermatol 27:507–516

110. Lee YS, Liang YC, Wu P, et al (2019) STAT3 signalling pathway is implicated in keloid pathogenesis by preliminary transcriptome and open chromatin analyses. Exp Dermatol 28:480–484

111. Liang C, Yen Y, Hung L, et al (2013) Thalidomide inhibits fibronectin production in TGF-β1-treated normal and keloid fibroblasts via inhibition of the p38/SMAD3 pathway. Biochem Pharmacol 85:1594–1602

112. Liao WT, Yu HS, Arbiser JL, et al (2010) Enhanced MCP-1 release by keloid CD14+ cells augments fibroblast proliferation: role of MCP-1 and Akt pathway in keloids. Exp Dermatol 19:e142–e150

113. Lim CP, Phan TT, Lim IJ, Cao X (2006) Stat3 contributes to keloid pathogenesis via promoting collagen production, cell proliferation and migration. Oncogene 25:5416–5425

114. Limandjaja GC, Belien JM, Scheper RJ, et al (2019) Hypertrophic and keloid scars fail to progress from the CD34-/α-smooth muscle actin (α-SMA)+ immature scar phenotype and show gradient differences in α-SMA and p16 expression. Br J Dermatol Jun 17:[Epub ahead of print]

115. Limandjaja GC, van den Broek LJ, Waaijman T, et al (2017) Increased epidermal thickness and abnormal epidermal differentiation in keloid scars. Br J Dermatol 176:116–126

116. Luo LF, Shi Y, Zhou Q, et al (2013) Insufficient expression of the melanocortin-1 receptor by human dermal fibroblasts contributes to excess collagen synthesis in keloid scars. Exp Dermatol 22:764–766

117. Luo L, Li J, Liu H, et al (2017) Adiponectin is involved in connective tissue growth factor-induced proliferation, migration and overproduction of the extracellular matrix in keloid fibroblasts. Int J Mol Sci 18:1–21

118. Ma X, Chen J, Xu B, et al (2015) Keloid-derived keratinocytes acquire a fibroblast-like appearance and an enhanced invasive capacity in a hypoxic microenvironment in vitro. Int J Mol Med 35:1246–1256

119. Machesney M, Tidman N, Waseem A, et al (1998) Activated keratinocytes in the epidermis of hypertrophic scars. Am J Pathol 152:1133–41

120. Maeda D, Kubo T, Kiya K, et al (2019) Periostin is induced by IL-4/IL-13 in dermal fibroblasts and promotes RhoA/ROCK pathway-mediated TGF-β1 secretion in abnormal scar formation. J Plast Surg Hand Surg May 8:1–7

121. Makino S, Mitsutake N, Nakashima M (2008) DHMEQ, a novel NF-kappaB inhibitor, suppresses growth and type I collagen accumulation in keloid fibroblasts. J Dermatol Sci 51:171—180

122. Mantel A, Newsome A, Thekkudan T, et al (2016) The role of aldo-keto reductase 1C3 (AKR1C3)-mediated prostaglandin D2 (PGD2) metabolism in keloids. Exp Dermatol 25:38–43

123. Materazzi S, Pellerito S, Di Serio C, et al (2007) Analysis of protease-activated receptor-1 and -2 in human scar formation. J Pathol 212:440–449

124. Meenakshi J, Jayaraman V, Ramakrishnan KM, Babu M (2005) Ultrastructural differentiation of abnormal scars. Ann Burn Fire Disasters 18:83–88

125. Meenakshi J, Vidyameenakshi S, Ananthram D, et al (2009) Low decorin expression along with inherent activation of ERK1,2 in earlobe keloids. Burns 35:519–526

126. Messadi D V., Doung HS, Zhang Q, et al (2004) Activation of NFκB signal pathways in keloid fibroblasts. Arch Dermatol Res 296:125–133

127. Meyer LJM, Russell SB, Russell JD, et al (2000) Reduced hyaluronan in keloid tissue and cultured keloid fibroblasts. J Invest Dermatol 114:953–959

128. Miller CC, Godeau G, Lebreton-DeCoster C, et al (2003) Validation of a morphometric method for evaluating fibroblast numbers in normal and pathologic tissues. Exp Dermatol 12:403–411

129. Mills BG, Frausto A, Brien E (2000) Cytokines associated with the pathophysiology of aggressive fibromatosis. J Orthop Res 18:655–662

130. Morgan MB, Purohit C, Anglin TR (2008) Immunohistochemical distinction of cutaneous spindle cell carcinoma. Am J Dermatopathol 30:228–232

131. Moshref S, Mufti ST (2009) Keloid and hypertrophic scars: comparative histopathological and immunohistochemical study. J King Abdulaziz Univ - Med Sci 17:3–22

132. Muffley LA, Zhu KQ, Engrav LH, et al (2011) Spatial and temporal localization of the melanocortin 1 receptor and its ligand α–melanocyte-stimulating hormone during cutaneous wound repair. J Histochem Cytochem 59:278–288

133. Mukhopadhyay A, Do D V., Ong CT, et al (2011) The role of stem cell factor and c-KIT in keloid pathogenesis: do tyrosine kinase inhibitors have a potential therapeutic role? Br J Dermatol 164:372–386

134. Mukhopadhyay A, Chan SY, Lim IJ, et al (2007) The role of the activin system in keloid pathogenesis. Am J Physiol Cell Physiol 292:C1331–C1338

135. Mukhopadhyay A, Fan S, Dang VD, et al (2010) The role of hepatocyte growth factor/c-Met system in keloid pathogenesis. J Trauma - Inj Infect Crit Care 69:1457–1466

136. Mukhopadhyay A, Wong MY, Chan SY, et al (2010) Syndecan-2 and decorin: proteoglycans with a difference-implications in keloid pathogenesis. J Trauma - Inj Infect Crit Care 68:999–1008

137. Murao N, Seino K ichiro, Hayashi T, et al (2014) Treg-enriched CD4+ T cells attenuate collagen synthesis in keloid fibroblasts. Exp Dermatol 23:266–271

138. Nagata H, Ueki H, Moriguchi T (1985) Fibronectin: localization in normal human skin, granulation tissue, hypertrophic scar, mature scar, progressive systemic sclerotic skin, and other fibrosing dermatoses. Arch Dermatol 121:995–999

139. Naitoh M, Hosokawa N, Kubota H, et al (2001) Upregulation of HSP47 and collagen type III in the dermal fibrotic disease, keloid. Biochem Biophys Res Commun 280:1316–1322

140. Narvaez D, Kanitakis J, Faure M, Claudy A (1996) Immunohistochemical study of CD34-positive dendritic cells of human dermis. Am J Dermatopathol 18:283–288

141. Nickoloff BJ (1991) The human progenitor cell antigen (CD34) is localized on endothelial cells, dermal dendritic cells, and perifollicular cells in formalin-fixed normal skin, and on proliferating endothelial cells and stromal spindle-shaped cells in Kaposi’s sarcoma. Arch Dermatol 127:523–529

142. Niessen FB, Andriessen MP, Schalkwijk J, et al (2001) Keratinocyte-derived growth factors play a role in the formation of hypertrophic scars. J Pathol 194:207–216

143. Niessen FB, Schalkwijk J, Vos H, Timens W (2004) Hypertrophic scar formation is associated with an increased number of epidermal Langerhans cells. J Pathol 202:121–129

144. Nirodi CS, Devalaraja R, Nanney LB, et al (2000) Chemokine and chemokine receptor expression in keloid and normal fibroblasts. Wound Repair Regen 8:371–382

145. Ogawa R, Akaishi S, Izumi M (2009) Histologic analysis of keloids and hypertrophic scars. Ann Plast Surg 62:104–105

146. Ohtsuru A, Yoshimoto H, Ishihara H, et al (2000) Insulin-like growth factor-I (IGF-I)/IGF-I receptor axis and increased invasion activity of fibroblasts in keloid. Endocr J 47 Suppl:S41–S44

147. Oliveira GV, Hawkins HK, Chinkes D, et al (2009) Hypertrophic versus non-hypertrophic scars compared by immunohistochemistyr and laser confocal microscopy: type I and III collagens. Int Wound J 6:445–452

148. Ong CT, Khoo YT, Mukhopadhyay A, et al (2010) Comparative proteomic analysis between normal skin and keloid scar. Br J Dermatol 162:1302–1315

149. Ong CT, Khoo YT, Tan EK, et al (2007) Epithelial–mesenchymal interactions in keloid pathogenesis modulate vascular endothelial growth factor expression and secretion. J Pathol 211:95–108

150. Onodera M, Ueno M, Ito O, et al (2007) Factor XIIIa-positive dermal dendritic cells in keloids and hypertrophic and mature scars. Pathol Int 57:337–342

151. Ooi BNS, Mukhopadhyay A, Masilamani J, et al (2010) Hepatoma-derived growth factor and its role in keloid pathogenesis. J Cell Mol Med 14:1328–1337

152. Pavelecini M, Zettler CG, Fernandes MC, Ely PB (2019) Experimental immunohistochemical expression of cyclooxygenases in hypertrophic scars and keloids. Plast Reconstr Surg Glob Open 7:1–7

153. Prathiba V, Rao KS, Gupta PD (2001) Altered expression of keratins during abnormal wound healing in human skin. Cytobios 104:43–51

154. Reno F, Sabbatini M, Stella M, et al (2005) Effect of in vitro mechanical compression on Epilysin (matrix metalloproteinase-28) expression in hypertrophic scars. Wound Repair Regen 13:255–261

155. Rossiello L, D’Andrea F, Grella R, et al (2009) Differential expression of cyclooxygenases in hypertrophic scar and keloid tissues. Wound Repair Regen 17:750–757

156. Rünger TM, Quintanilla-Dieck MJ, Bhawan J (2007) Role of cathepsin K in the turnover of the dermal extracellular matrix during scar formation. J Invest Dermatol 127:293–297

157. Russell SB, Russell JD, Trupin KM, et al (2010) Epigenetically altered wound healing in keloid fibroblasts. J Invest Dermatol 130:2489–2496

158. Sadick H, Herberger A, Riedel K, et al (2008) TGF-β1 antisense therapy modulates expression of matrix metalloproteinases in keloid-derived fibroblasts. Int J Mol Med 22:55–60

159. Saffari TM, Bijlard E, van Bodegraven EAM, et al (2018) Sensory perception and nerve fibre innervation in patients with keloid scars: an investigative study. Eur J Dermatology 28:828–829

160. Salem A, Salem A, Assaf M, et al (2009) Role of vascular endothelial growth factor in keloids: a clinicopathologic study. Int J Dermatol 48:1071–7

161. Salgado RM, Alcántara L, Mendoza-Rodríguez CA, et al (2012) Post-burn hypertrophic scars are characterized by high levels of IL-1β mRNA and protein and TNF-α type I receptors. Burns 38:668–676

162. Santucci M, Borgognoni L, Reali UM, Gabbiani G (2001) Keloids and hypertrophic scars of Caucasians show distinctive morphologic and immunophenotypic profiles. Virchows Arch 438:457–463

163. Sato M (2006) Upregulation of the Wnt/β-catenin pathway induced by transforming growth factor-β in hypertrophic scars and keloids. Acta Derm Venereol 86:300–307

164. Sayah DN, Soo C, Shaw WW, et al (1999) Downregulation of apoptosis-related genes in keloid tissues. J Surg Res 87:209–216

165. Schmid P, Itin P, Cherry G, et al (1998) Enhanced expression of transforming growth factor-β type I and type II receptors in wound granulation tissue and hypertrophic scar. Am J Pathol 152:485–93

166. Scott PG, Dodd CM, Tredget EE, et al (1995) Immunohistochemical localization of the proteoglycans decorin, biglycan and versican and transforming growth factor-β in human post-burn hypertrophic and mature scars. Histopathology 26:423–431

167. Seleit I, Bakry OA, Samaka RM, Tawfik AS (2016) Immunohistochemical evaluation of leptin expression in wound healing: a clue to exuberant scar formation. Appl Immunohistochem Mol Morphol 24:296–306

168. Shaker SA, Ayuob NN, Hajrah NH (2011) Cell talk: a phenomenon observed in the keloid scar by immunohistochemical study. Appl Immunohistochem Mol Morphol 19:153–159

169. Shang T, Yao B, Gao D, et al (2018) A novel model of humanised keloid scarring in mice. Int Wound J 15:90–94

170. Sharquie KE, Al-Dhalimi MA (2003) Keloid in Iraqi patients: a clinicohistopathologic study. Dermatologic Surg 29:847–851

171. Shi J-H, Hu D-H, Zhang Z-F, et al (2012) Reduced expression of microtubule-associated protein 1 light chain 3 in hypertrophic scars. Arch Dermatol Res 304:209–215

172. Shin JU, Kim SH, Kim H, et al (2016) TSLP is a potential initiator of collagen synthesis and an activator of CXCR4/SDF-1 axis in keloid pathogenesis. J Invest Dermatol 136:507–515

173. Sible JC, Eriksson E, Smith SP, Oliver N (1994) Fibronectin gene expression differs in normal and abnormal human wound healing. Wound Repair Regen 2:3–19

174. Sideek MA, Teia A, Kopecki Z, et al (2016) Co-localization of LTBP-2 with FGF-2 in fibrotic human keloid and hypertrophic scar. J Mol Histol 47:35–45

175. Sidgwick GP, Iqbal SA, Bayat A (2013) Altered expression of hyaluronan synthase and hyaluronidase mRNA may affect hyaluronic acid distribution in keloid disease compared with normal skin. Exp Dermatol 22:377–379

176. Simon F, Bergeron D, Larochelle S, et al (2012) Enhanced secretion of TIMP-1 by human hypertrophic scar keratinocytes could contribute to fibrosis. Burns 38:421–427

177. Slominski A, Wortsman J, Mazurkiewicz JE, et al (1993) Detection of proopiomelanocortin-derived antigens in normal and pathologic human skin. J Lab Clin Med 122:658–66

178. Smoller BR, McNutt NS, Hsu A (1989) HMB-45 recognizes stimulated melanocytes. J Cutan Pathol 16:49–53

179. Staton CA, Valluru M, Hoh L, et al (2010) Angiopoietin-1, angiopoietin-2 and Tie-2 receptor expression in human dermal wound repair and scarring. Br J Dermatol 163:920–927

180. Suarez E, Syed F, Alonso-Rasgado T, Bayat A (2015) Identification of biomarkers involved in differential profiling of hypertrophic and keloid scars versus normal skin. Arch Dermatol Res 307:115–133

181. Suttho D, Mankhetkorn S, Binda D, et al (2017) 3D modeling of keloid scars in vitro by cell and tissue engineering. Arch Dermatol Res 309:55–62

182. Syed F, Ahmadi E, Iqbal SA, et al (2011) Fibroblasts from the growing margin of keloid scars produce higher levels of collagen I and III compared with intralesional and extralesional sites: clinical implications for lesional site-directed therapy. Br J Dermatol 164:83–96

183. Syed F, Bayat A (2012) Notch signaling pathway in keloid disease: enhanced fibroblast activity in a Jagged-1 peptide-dependent manner in lesional vs. extralesional fibroblasts. Wound Repair Regen 20:688–706

184. Szulgit G, Rudolph R, Wandel A, et al (2002) Alterations in fibroblast α1β1 integrin collagen receptor expression in keloids and hypertrophic scars. J Invest Dermatol 118:409–415

185. Tan EML, Hoffren J, Rouda S, et al (1993) Decorin, versican, and biglycan gene expression by keloid and normal dermal fibroblasts: differential regulation by basic fibroblast growth factor. Exp. Cell Res. 209:200–207

186. Tan KT, McGrouther DA, Day AJ, et al (2011) Characterization of hyaluronan and TSG-6 in skin scarring: differential distribution in keloid scars, normal scars and unscarred skin. J Eur Acad Dermatology Venereol 25:317–327

187. Tanaka A, Hatoko M, Tada H, et al (2004) Expression of p53 family in scars. J Dermatol Sci 34:17–24

188. Teofoli P, Barduagni S, Ribuffo M, et al (1999) Expression of Bcl-2, p53, c-jun and c-fos protooncogenes in keloids and hypertrophic scars. J Dermatol Sci 22:31–37

189. Theoret CL, Acvs D, Olutoye OO, et al (2013) Equine exuberant granulation tissue and human keloids: a comparative histopathologic study. Vet Surg 42:783–789

190. Touchi R, Ueda K, Kurokawa N, Tsuji M (2016) Central regions of keloids are severely ischaemic. J Plast Reconstr Aesthetic Surg 69:e35–e41

191. Tuan TL, Wu H, Huang EY, et al (2003) Increased plasminogen activator inhibitor-1 in keloid fibroblasts may account for their elevated collagen accumulation in fibrin gel cultures. Am J Pathol 162:1579–1589

192. Ueda K, Furuya E, Yasuda Y, et al (1999) Keloids have continuous high metabolic activity. Plast. Reconstr. Surg. 104:694–678

193. Ueda K, Yasuda Y, Furuya E, Oba S (2004) Inadequate blood supply persists in keloids. Scand J Plast Reconstr Surg Hand Surg 38:267–271

194. Van Der Veer WM, Niessen FB, Ferreira JA, et al (2011) Time course of the angiogenic response during normotrophic and hypertrophic scar formation in humans. Wound Repair Regen 19:292–301

195. Velangi SS, Rees JL (2001) Why are scars pale? An immunohistochemical study indicating preservation of melanocyte number and function in surgical scars. Acta Derm Venereol 81:326–328

196. Verhaegen PDHM, Van Zuijlen PPM, Pennings NM, et al (2009) Differences in collagen architecture between keloid, hypertrophic scar, normotrophic scar, and normal skin: an objective histopathological analysis. Wound Repair Regen 17:649–656

197. Wang X, Liu Y, Deng Z, et al (2009) Inhibition of dermal fibrosis in self-assembled skin equivalents by undifferentiated keratinocytes. J Dermatol Sci 53:103–111

198. Yagi Y, Muroga E, Naitoh M, et al (2013) An ex vivo model employing keloid-derived cell-seeded collagen sponges for therapy development. J Invest Dermatol 133:386–393

199. Yamawaki S, Naitoh M, Kubota H, et al (2018) HtrA1 is specifically up-regulated in active keloid lesions and stimulates keloid development. Int J Mol Sci 19:1–12

200. Yan C, Grimm WA, Garner WL, et al (2010) Epithelial to mesenchymal transition in human skin wound healing is induced by tumor necrosis factor-α through bone morphogenic protein-2. Am J Pathol 176:2247–2258

201. Yan L, Cao R, Wang L, et al (2015) Epithelial-mesenchymal transition in keloid tissues and TGF-β1-induced hair follicle outer root sheath keratinocytes. Wound Repair Regen 23:601–610

202. Yang S, Geng Z, Ma K, et al (2016) Comparison of the histological morphology between normal skin and scar tissue. J Huazhong Univ Sci Technol 36:265–269

203. Yang S, Sun Y, Geng Z, et al (2016) Abnormalities in the basement membrane structure promote basal keratinocytes in the epidermis of hypertrophic scars to adopt a proliferative phenotype. Int J Mol Med 37:1263–1273

204. Yoshimoto H, Ishihara H, Ohtsuru A, et al (1999) Overexpression of insulin-like growth factor-1 (IGF-I) receptor and the invasiveness of cultured keloid fibroblasts. Am J Pathol 154:883–889

205. Young M, Eun L (2018) Interleukin-31, interleukin-31RA, and OSMR expression levels in post-burn hypertrophic scars. J Pathol Transl Med 52:307–313

206. Yu D, Shang Y, Yuan J, et al (2016) Wnt/β-catenin signaling exacerbates keloid cell proliferation by regulating telomerase. Cell Physiol Biochem 39:2001–2013

207. Zhang F, Wang H, Wang X, et al (2015) TGF-β induces M2-like macrophage polarization via SNAIL mediated suppression of a pro-inflammatory phenotype. Oncotarget 7:52294–52306

208. Zhang K, Garner W, Cohen L, et al (1995) Increased types I and III collagen and transforming growth factor-β1 mRNA and protein in hypertrophic burn scar. J Invest Dermatol 104:750–754

209. Zhang Q, Tao K, Huang W, et al (2013) Elevated expression of pleiotrophin in human hypertrophic scars. J Mol Histol 44:91–96

210. Zhang Q, Cai L, Wang M, et al (2018) Identification of a novel mutation in the mechanoreceptor-encoding gene CXCR1 in patients with keloid. Arch Dermatol Res 310:561–566

211. Zhang Y, Zhang Z, Liu Y, et al (2015) Requirement of Gαi1/3-Gab1 signaling complex for keratinocyte growth factor-induced PI3K-AKT-mTORC1 activation. J Invest Dermatol 135:181–191

212. Zhao J, Zhong A, Friedrich EE, et al (2017) S100A12 induced in the epidermis by reduced hydration activates dermal fibroblasts and causes dermal fibrosis. J Invest Dermatol 137:650–659

213. Zheng J, Song F, Lu SL, Wang XQ (2014) Dynamic hypoxia in scar tissue during human hypertrophic scar progression. Dermatologic Surg 40:511–518

214. Zhou HM, Wang J, Elliott C, et al (2010) Spatiotemporal expression of periostin during skin development and incisional wound healing: lessons for human fibrotic scar formation. J Cell Commun Signal 4:99–107
