# Supplementary material for: The Keloid Disorder: Heterogeneity, Histopathology, Mechanisms and Models
Source: Front Cell Dev Biol. 2020 May 26;8:360. doi: 10.3389/fcell.2020.00360 (PMC7264387; doi:10.3389/fcell.2020.00360)
Supplement: Supplementary file 2 [file Table_2.DOCX]

**Supplemental table 2A.** Differences between peripheral and central keloid regions

| **Category** | **Parameter** | **Periphery** | **Centre** | **References** |
| --- | --- | --- | --- | --- |
| Symptoms | pigmentation  erythematous, inflamed | ↑  +, ↑↑ | regularly or ↓  −, > | [7, 27, 30, 31]  [7, 11, 16, 27, 30, 31], [25] |
|  | thickness  active growth | elevation  + | involution, soft  − | [11, 16, 30, 31]  [37] |
|  | sensation | itching | pain | [36] |
| Tension/Stiffness | tension, stiffness | ↑, ↑ | ↓, ↓ | [2, 6] |
| Epidermis | vimentin (EMT marker) | ↑↑ | ↑ | [25] |
| Dermal cells | cellularity | ↑ | ↓ | [3, 4, 15, 26, 56] |
|  | *apoptotic cells* | ↑ | − | [3, 4, 45] |
| Fibroblasts | cell activity based on cell shape | ↑ (round) | ↓ (elongated) | [30] |
|  | cell membrane: linoleic acid; oleic acid | ↑; < | ↓; ↑ | [30] |
|  | p53, bcl-2, Fas | ↑, ↑, < | >, >, ↑ | [26] |
|  | PML (senescence); TSG-6 | ↑; + | ↑↑; ↓ | [56]; [51] |
| Fibroblasts  (*in vitro*) | *proliferation rate*  proliferation rate | *+*  ↑ | *↑*  ↓ | [54] [12]*  [49]* [56] |
|  | cell cycle phases | G2, S | G0-G1 | [31, 55]* |
|  | senescence | ↑ | ↑↑ | [56] |
|  | anti-apoptotic gene AVEN  pro-apoptotic genes (e.g. ADAM12); p53 | ↑  +; < | +  ↑; ↑ | [47]  [47]; [31]* |
|  | contraction in collagen gel | ↓ | ↑ | [49] |
|  | collagen I and III | ↑ | + | [50] |
|  | MMP-3, THSB2 | ↓ | + | [47] |
|  | MMP-19, STAT1, PRG1 | + | ↑ Cd | [47] |
|  | MMP-1/2/3/9 and MT1-MMP | ↑ | > Cs, ↑ Cd | [28]‡ |
|  | *TGFβRI, Smad2, Smad3* | *+* | *↑* | [54]* |
|  | *IL-6, VEGF; GDF-9* | *+, +; ↑* | *↑, ↑;* ↓ | [12]*; [11] |
|  | secretion of INHBA, MCP-1  secretion of MMP-3 | ↓, ↑  ↓↓ | + Cd, ↑↑ Cd  ↓↓ Cd, ↓ Cs | [47] |
|  | neuregulin-1 (growth factor) | ↑↑ | ↑ | [22] |
|  | sensitivity to photodynamic therapy | < | (< Cs) ↑ Cd | [33] |
|  | + resveratrol: collagen I, α-SMA, HSP47  TGF-β1, early apoptosis, late apoptosis | ↓  ↓↓, ↑, ↑↑ | ↓↓  ↓, ↑↑, ↑ | [16] |
| ECM | hypereosinophilic collagen bundles | − | ↑ | [3] |
|  | glazed area with larger collagen fibers densely packed, irregular | −  − | +  + | [23]  [23] |
|  | keloidal collagen | < | ↑ | [14, 15, 17, 38, 44] |
|  | nodules | large | small | [15] |
|  | collagen orientation | random | parallel | [6, 15] |
|  | CD34, CD34 / CD90 / CD117 | −; ↓ | +; ↓↓ Cs, Cd + | [1]‡; [18] |
|  | collagen synthesis (P4H+) | ↑ | ↓ | [1]‡ |
|  | collagen I; collagen III | ↑; ↑, − | >; >, + | [38, 50]; [52], [38, 50] |
|  | collagen VI | ↑ | > | [38, 52] |
|  | TGF-β1 | ↑ | > |  |
| Vasculature | vascular density | ↑ | ↓ | [4, 27, 53] |
|  | ischemia (HIF-1α) | ↑↑ | ↑ | [53] |
|  | microvessels | >, parallel | ↓, flattened | [15], [15, 24] |
| Nerve cells | nerve fiber density | ↓ | ↓↓ | [43] |
| Immune cells | lymphocyte infiltration | ↑ | > | [4, 20] |
|  | CD45+ inflammatory cells | ↑ | > | [27] |
| Senescence | telomere length | ↑ | ↑↑ Cd | [13] |
| Laser capture microdissection | hyperproliferation, pro-inflammatory, EMT | epidermis: ↑ | > | [21] |
|  | fibrosis, inflammation, apoptosis | < | epidermis: ↑ | [21] |
|  | EMT, immune modulation, keloid margin-related migration | dermis: ↑ | > | [21] |
| Proteomics | mitochondrial and structural proteins, K2 | ↑, + | +, ↑ | [19] |
| Skin equivalent  *(in vitro)* | contraction, epidermal thickness, secretory HGF & *COL4A2* downregulation | < | ↑↑ Cd, Cs ↑ | [29] |
| Conditioned media | effect on NF/NsF:  proliferation, migration; collagen I, fibronectin, α-SMA, PAI-1, TGF-β, CTGF | ↑↑  ↑↑ | ↑  ↑ | [5] |

**Supplemental table 2A.** Differences between peripheral and central keloid regions, parameter expression listed as compared to control groups (normal skin and/or normal scar). Table contains all publications in which peripheral and central keloid regions are studied and were found to exhibit differences. Legend; italicized font: studies in which the keloid centre was more aggressive than the periphery; normal non-italicized font: studies in which the keloid periphery was more aggressive than the centre; (in vitro): data from in vitro culture studies; +: present, normal expression or values; ↑: increased; −: absent; ↓: decreased; < or >: less than Hscar or Kscar, used for parameters when Hscar and Kscar were compared to each other and neither were compared to normal skin or normotrophic scar; ≈: similar to normal skin and/or normal scar; *explant fibroblast cell isolation; unless stated otherwise, fibroblasts were isolated via enzymatic digestion; ‡: results based on n=1. Abbreviations in alphabetical order; Cs: central superficial keloid region; Cd: central deep keloid region; *COL4A2*: collagen type IV α2 chain; ECM: extracellular matrix; EMT: epithelial-mesenchymal transition; GDF-9: growth differentiation factor 9; HSP: heat shock protein; INHBA: inhibin, beta A; IL: interleukin; K2: keratin 2; NF: normal skin fibroblasts; NsF: normotrophic scar fibroblasts; PML: promyelocytic leukemia protein (tumor suppressor, induces senescence in fibroblasts); Smad: mothers against decapentaplegic homolog 1 (Drosophila); STAT1: signal transducer and activator of transcription 1; TGF-β: transforming growth factor beta; TGFβR: transforming growth factor beta receptor; TSG-6: tumour necrosis factor-inducible gene 6 protein; VEGF: vascular endothelial growth factor. N.B. any additional information on parameters listed in abbreviations are all derived from cited literature in table.

**Supplemental table 2B.** Intralesional keloid heterogeneity otherwise defined

| **Regions within keloids** | **References** |
| --- | --- |
| **Keloid tissue explants (cultured up to 10 days):**  - epidermis + upper dermis: major site of collagenase production (similar to Nskin)  - keloid nodule (middle dermis): virtually no collagenase  - fibrous capsule (deeper dermis): virtually no collagenase | [34] |
| **Fourier transform infrared microspectroscopy:**  - differential chemical maps in peripheral, superficial and deep regions | [14] |
| **Histological regions within keloids:**  - zone of hyalinising collagen bundles  - fine fibrous areas  - area of inflammation  - zone of dense regular connective tissue  - nodular fibrous area, area of angiogenesis | [8] |
| **Androgen binding capacity and DNA contents:**  - clinically active keloid regions: ↑  - inactive keloid regions | [46] |
| **FOXO1 expression (associated with scarring):**  - margin areas: ↑  - mature sites | [35] |
| **Histological distinction between 3 dermal regions:**  - superficial dermis:  ↑ fibroblast activity, ↑ fibroblast migration, ↑ B- & T-lymphocytes  fine collagen bundles, parallel organisation  lowest type I collagen levels, highest type III collagen levels  - middle dermis:  ↑ fibroblast density, only few fibroblasts migrate  dense ECM, thickest layer, parallel organisation  increasing type I and decreasing type III collagen  - deep dermis:  atrophic and degenerate cells, no fibroblast migration  abnormally thick and hyalinized collagen bundles, random and loosely organised  highest type I collagen levels, lowest type III collagen levels | [20]* |
| **Superficial, central and basal keloid dermis:**  - central fibroblasts: ↑ proliferation, ↑ cell density, formed 3D structure by contracting self-  produced ECM; ↓ apoptosis  - superficial and basal fibroblasts: normal proliferation, do not develop 3D structure; ↓ apoptosis | [32]* |
| **Superficial and deep fibroblasts response to hydrocortisone**  - superficial fibroblasts: normal ↓ elastin  - deep nodular fibroblasts: ↑ elastin | [39, 41]* |
| **Expression of fibrosis genes:**  - deep dermal fibroblasts: ↑ collagen I, TGF-β1, periostin, PAI-2, inhibin A genes  - superficial dermal fibroblasts: slightly ↑ follistatin, secreted frizzled-related protein 2 genes | [48]‡ |
| **Full thickness keloid skin equivalent implanted in animal model:**  - with deep dermal fibroblasts: ↑ dermal thickness, ↑ collagen I  - with superficial deep dermal fibroblasts: ↑ area (spreading), normal collagen I | [48]‡ |
| **Three distinct structural parts** underneath normal dermal region; differ in fibroblast, blood vessel, inflammatory cell & mast cell density: from top to bottom  - keloidal collagen  - organizing collagen  - proliferating core collagen: this core thought to be key for tumour growth and recurrence | [9, 10] |
| **Active and inactive regions:**  - active, clinically aggressive areas: ↓ SFRP1, ↓ SFRP2, ↑ IGFBP5;  strong redness and itching, greatly upregulated collagen III expression  - inactive, relatively quiescent areas: normal SFRP1, SFRP2, IGFBP5 | [40]; [37] |
| **Regions within keloid nodules:**  - hypoxic inner zone: ↓ vascularity, ↑ HIF-1α; ↑ autophagy and glycolysis in fibroblasts  - normoxic outer zone: circular layer of collagen bundles rich in vascularity, ↑ HIF-2α;  ↓ autophagy and glycolysis in fibroblasts | [42] |

**Supplemental table 2B.** Intralesional keloid heterogeneity otherwise defined, parameter expression listed as compared to control groups (normal skin and/or normal scar). Table contains all publications in which different regions were distinguished within keloid scars, which could not be classified as peripheral or central keloid regions. Legend; +: present, normal expression or values; ↑: increased; −: absent; ↓: decreased; ≈: similar to normal skin and/or normal scar; *explant fibroblast cell isolation; unless stated otherwise, fibroblasts were isolated via enzymatic digestion; ‡: results based on n=1. Abbreviations in alphabetical order; 3D: three dimensional; ECM: extracellular matrix; FOXO1: forkhead box protein O1; HIF-1/2α: hypoxia-inducible factor 1/2 alpha; IGFBP: insulin-like growth factor binding protein; Nskin: normal skin; PAI-2: plasminogen activator inhibitor 2; SFRP1/2: secreted frizzled-related protein 1/2 (Wnt inhibitor); TGF-β1: transforming growth factor beta 1. N.B. any additional information on parameters listed in abbreviations are all derived from cited literature in table.

**References**

1. Aiba S, Tagami H (1997) Inverse correlation between CD34 expression and proline-4 hydroxyase immunoreactivity on spindle cells noted in hypertrophic scars and keloids. J Cutan Pathol 24:65–69

2. Akaishi S, Akimoto M, Ogawa R, Hyakusoku H (2008) The relationship between keloid growth pattern and stretching tension: visual analysis using the finite element method. Ann Plast Surg 60:445–451

3. Akasaka Y, Fujita K, Ishikawa Y, et al (2001) Detection of apoptosis in keloids and a comparative study on apoptosis between keloids, hypertrophic scars, normal healed flat scars, and dermatofibroma. Wound Repair Regen 9:501–506

4. Appleton I, Brown NJ, Willoughby DA (1996) Apoptosis, necrosis, and proliferation: possible implications in the etiology of keloids. Am J Pathol 149:1441–1447

5. Ashcroft KJ, Syed F, Bayat A (2013) Site-specific keloid fibroblasts alter the behaviour of normal skin and normal scar fibroblasts through paracrine signalling. PLoS One 8:e75600

6. Aya R, Yamawaki S, Yoshikawa K, et al (2015) The shear wave velocity on elastography correlates with the clinical symptoms and histopathological features of keloids. Plast Reconstr Surg - Glob Open 3:e464

7. Bella H, Heise M, Yagi KI, et al (2011) A clinical characterization of familial keloid disease in unique African tribes reveals distinct keloid phenotypes. Plast Reconstr Surg 127:689–702

8. Bux S, Madaree A (2010) Keloids show regional distribution of proliferative and degenerate connective tissue elements. Cells Tissues Organs 191:213–234

9. Chong Y, Kim CW, Kim YS, et al (2018) Complete excision of proliferating core in auricular keloids significantly reduces local recurrence: a prospective study. J Dermatol 45:139–144

10. Chong Y, Park TH, Seo SW, Chang CH (2015) Histomorphometric analysis of collagen architecture of auricular keloids in an Asian population. Dermatologic Surg 41:415–422

11. Gao Z, Wu X, Song N, et al (2010) Differential expression of growth differentiation factor-9 in keloids. Burns 36:1289–1295

12. Giugliano G, Pasquali D, Notaro A, et al (2003) Verapamil inhibits interleukin-6 and vascular endothelial growth factor production in primary cultures of keloid fibroblasts. Br J Plast Surg 56:804–809

13. Granick M, Kimura M, Kim S, et al (2011) Telomere dynamics in keloids. Eplasty 11:e15

14. Hollywood KA, Maatje M, Shadi IT, et al (2010) Phenotypic profiling of keloid scars using FT-IR microspectroscopy reveals a unique spectral signature. Arch Dermatol Res 302:705–715

15. Huang C, Akaishi S, Hyakusoku H, Ogawa R (2014) Are keloid and hypertrophic scar different forms of the same disorder? A fibroproliferative skin disorder hypothesis based on keloid findings. Int Wound J 11:517–522

16. Ikeda K, Torigoe T, Matsumoto Y, et al (2013) Resveratrol inhibits fibrogenesis and induces apoptosis in keloid fibroblasts. Wound Repair Regen 21:616–623

17. Imaizumi R, Akasaka Y, Inomata N, et al (2009) Promoted activation of matrix metalloproteinase (MMP)-2 in keloid fibroblasts and increased expression of MMP-2 in collagen bundle regions: Implications for mechanisms of keloid progression. Histopathology 54:722–730

18. Iqbal SA, Syed F, McGrouther DA, et al (2010) Differential distribution of haematopoietic and nonhaematopoietic progenitor cells in intralesional and extralesional keloid: do keloid scars provide a niche for nonhaematopoietic mesenchymal stem cells? Br J Dermatol 162:1377–1383

19. Javad F, Day PJ (2012) Protein profiling of keloidal scar tissue. Arch Dermatol Res 304:533–540

20. Jiao H, Zhang T, Fan J, Xiao R (2017) The superficial dermis may initiate keloid formation: histological analysis of the keloid dermis at different depths. Front Physiol 8:1–9

21. Jumper N, Hodgkinson T, Paus R, Bayat A (2017) Site-specific gene expression profiling as a novel strategy for unravelling keloid disease pathobiology. PLoS One 12:e0172955

22. Jumper N, Hodgkinson T, Paus R, Bayat A (2017) A role for Neuregulin-1 in promoting keloid fibroblast migration. Acta Derm Venereol 97:675–684

23. Kischer C (1984) Comparative ultrastructure of hypertrophic scars and keloids. Scan Electron Microsc (Pt 1):423–431

24. Kurokawa N, Ueda K, Tsuji M (2010) Study of microvascular structure in keloid and hypertrophic scars: density of microvessels and the efficacy of three-dimensional vascular imaging. J Plast Surg Hand Surg 44:272–277

25. Kuwahara H, Tosa M, Murakami M, et al (2016) Examination of epithelial mesenchymal transition in keloid tissues and possibility of keloid therapy target. Plast Reconstr Surg Glob Open 4:1–7

26. Ladin DA, Hou Z, Patel D, et al (1998) P53 and apoptosis alterations in keloids and keloid fibroblasts. Wound Repair Regen 6:28–37

27. Le AD, Zhang Q, Wu Y, et al (2004) Elevated vascular endothelial growth factor in keloids: relevance to tissue fibrosis. Cells Tissues Organs 176:87–94

28. Li H, Nahas Z, Feng F, et al (2013) Tissue engineering for in vitro analysis of matrix metalloproteinases in the pathogenesis of keloid lesions. JAMA Facial Plast Surg 15:448–456

29. Limandjaja GC, Broek LJ van den, Waaijman T, et al (2018) Reconstructed human keloid models show heterogeneity within keloid scars. Arch Dermatol Res 310:815–826

30. Louw L, van der Westhuizen J, Duyvene de Wit L, Edwards G (1997) Keloids: peripheral and central differences in cell morphology and fatty acid compositions of lipids. Adv Exp Med Biol 407:515–520

31. Lu F, Gao J, Ogawa R, et al (2007) Biological differences between fibroblasts derived from peripheral and central areas of keloid tissues. Plast Reconstr Surg 120:625–630

32. Luo S, Benathan M, Raffoul W, et al (2001) Abnormal balance between proliferation and apoptotic cell death in fibroblasts derived from keloid lesions. Plast. Reconstr. Surg. 107:87–96

33. Mendoza J, Sebastian A, Allan E, et al (2012) Differential cytotoxic response in keloid fibroblasts exposed to photodynamic therapy is dependent on photosensitiser precursor, fluence and location of fibroblasts within the lesion. Arch Dermatol Res 304:549–562

34. Milsom JP, Craig RDP (1973) Collagen degradation in cultured keloid and hypertrophic scar tissue. Br J Dermatol 89:635–644

35. Mori R, Tanaka K, De Kerckhove M, et al (2014) Reduced FOXO1 expression accelerates skin wound healing and attenuates scarring. Am J Pathol 184:2465–2479

36. Na GY, Seo SK, Lee SJ, et al (2004) Upregulation of the NNP-1 (novel nuclear protein-1, D21S2056E) gene in keloid tissue determined by cDNA microarray and in situ hybridization. Br J Dermatol 151:1143–1149

37. Naitoh M, Hosokawa N, Kubota H, et al (2001) Upregulation of HSP47 and collagen type III in the dermal fibrotic disease, keloid. Biochem Biophys Res Commun 280:1316–1322

38. Peltonen J, Hsiao LL, Jaakkola S, et al (1991) Activation of collagen gene expression in keloids: co-localization of type I and VI collagen and transforming growth factor-beta 1 mRNA. J. Invest. Dermatol. 97:240–248

39. Russell SB, Trupin JS, Myers JC, et al (1989) Differential glucocorticoid regulation of collagen mRNAs in human dermal fibroblasts. Keloid-derived and fetal fibroblasts are refractory to down-regulation. J Biol Chem 264:13730–13735

40. Russell SB, Russell JD, Trupin KM, et al (2010) Epigenetically altered wound healing in keloid fibroblasts. J Invest Dermatol 130:2489–2496

41. Russell SB, Trupin JS, Kennedy RZ, et al (1995) Glucocorticoid regulation of elastin synthesis in human fibroblasts: down-regulation in fibroblasts from normal dermis but not from keloids. J Invest Dermatol 104:241–245

42. Ryoko O, Ito Y, Eid N, et al (2018) Upregulation of autophagy and glycolysis markers in keloid hypoxic-zone fibroblasts: morphological characteristics and implications. Histol Histopathol 33:1075–1087

43. Saffari TM, Bijlard E, van Bodegraven EAM, et al (2018) Sensory perception and nerve fibre innervation in patients with keloid scars: an investigative study. Eur J Dermatology 28:828–829

44. Santucci M, Borgognoni L, Reali UM, Gabbiani G (2001) Keloids and hypertrophic scars of Caucasians show distinctive morphologic and immunophenotypic profiles. Virchows Arch 438:457–463

45. Sayah DN, Soo C, Shaw WW, et al (1999) Downregulation of apoptosis-related genes in keloid tissues. J Surg Res 87:209–216

46. Schierle HP, Sholz D, Lemperle G (1997) Elevated levels of testosterone receptors in keloid tissue: an experimental investigation. Plast Reconstr Surg 100:390–395

47. Seifert O, Bayat A, Geffers R, et al (2008) Identification of unique gene expression patterns within different lesional sites of keloids. Wound Repair Regen 16:254–265

48. Supp DM, Hahn JM, Glaser K, et al (2012) Deep and superficial keloid fibroblasts contribute differentially to tissue phenotype in a novel in vivo model of keloid scar. Plast Reconstr Surg 129:1259–1271

49. Suttho D, Mankhetkorn S, Binda D, et al (2017) 3D modeling of keloid scars in vitro by cell and tissue engineering. Arch Dermatol Res 309:55–62

50. Syed F, Ahmadi E, Iqbal SA, et al (2011) Fibroblasts from the growing margin of keloid scars produce higher levels of collagen I and III compared with intralesional and extralesional sites: clinical implications for lesional site-directed therapy. Br J Dermatol 164:83–96

51. Tan KT, McGrouther DA, Day AJ, et al (2011) Characterization of hyaluronan and TSG-6 in skin scarring: differential distribution in keloid scars, normal scars and unscarred skin. J Eur Acad Dermatology Venereol 25:317–327

52. Theocharidis G, Drymoussi Z, Kao AP, et al (2016) Type VI collagen regulates dermal matrix assembly and fibroblast motility. J Invest Dermatol 136:74–83

53. Touchi R, Ueda K, Kurokawa N, Tsuji M (2016) Central regions of keloids are severely ischaemic. J Plast Reconstr Aesthetic Surg 69:e35–e41

54. Tsujita-Kyutoku M, Uehara N, Matsuoka Y, et al (2005) Comparison of transforming growth factor-beta/Smad signaling between normal dermal fibroblasts and fibroblasts derived from central and peripheral areas of keloid lesions. In Vivo (Brooklyn) 19:959–963

55. Tucci-Viegas VM, Hochman B, Frana JP, Ferreira LM (2010) Keloid explant culture: a model for keloid fibroblasts isolation and cultivation based on the biological differences of its specific regions. Int Wound J 7:339–348

56. Varmeh S, Egia A, McGrouther D, et al (2011) Cellular senescence as a possible mechanism for halting progression of keloid lesions. Genes and Cancer 2:1061–1066
