# Supplementary material for: The Keloid Disorder: Heterogeneity, Histopathology, Mechanisms and Models
Source: Front Cell Dev Biol. 2020 May 26;8:360. doi: 10.3389/fcell.2020.00360 (PMC7264387; doi:10.3389/fcell.2020.00360)
Supplement: Supplementary file 3 [file Table_3.DOCX]

**Supplemental table 3A.** Cellular abnormalities in keloid epidermis

| **Cell type** | **Material** | **Keloid parameters** | **Reference** |
| --- | --- | --- | --- |
| **Keratinocytes** | tissue | **epidermal thickness**  normal, ↓ epidermal cell layers | [12, 83, 151, 160], [19] |
|  |  | ↑ epidermal cell layers in some | [54, 145, 206] |
|  |  | ↑ epidermal cell layers | [20, 24, 42, 64, 77, 97, 127, 133, 134, 177, 205, 210, 218, 222, 224] |
|  | tissue | **abnormal intercellular adhesion features**  ↓ hemidesmosomes and altered desmosome shape  ↓ desmosomal proteins | [77]  [66] |
|  | tissue | **stratum corneum function**  ↑ high frequency conductance, ↑ TEWL, ↑ SC turnover  ↑ SC disorganization | [215, 219]  [134] |
|  | tissue  monolayer | **proliferation**  ↑ K6/K16, ↑ Ki67; normal K6/K16/K17, normal Ki67  normal viability (MTT assay), normal proliferation | [27, 177]; [133, 134]  [66], [134] |
|  | tissue | **apoptosis**  ↑ apoptosis; ↑ p65; Bcl-2 +, c-jun +; p53 –, + | [14]; [143]; [229]; [229], [76] |
|  | tissue | **differentiation**  K1/K10 +, K2e variable ↑;  ↑ involucrin; normal loricrin, filaggrin, SPRR2, SKALP | [27, 134, 177];  [133, 134]; [134] |
|  | monolayer | ↑ involucrin | [134] |
|  | monolayer | **migration**  cells form looser colonies, more dispersed  cells more rounded, less adherent to culture plastic | [67, 221]  [221] |
|  |  | **epithelial-mesenchymal transition** |  |
|  | tissue | ↑ EMT-marker proteins  - E-cadherin +, ↓  - ↑ β-catenin  - ↑ vimentin, ↑ α-SMA, ↑ FSP1  - ↑ TGF-β1, ↑ SMAD3 | [111], [67, 260]  [42, 67]  [67, 111, 260]  [260] |
|  | tissue | ↑ EMT-marker genes  - normal FGFR2-IIIb, ↑ FGFR-IIIc, ↓ TAp63  - normal snail 1, ↑ snail 2  - ↑ microRNA mir-21-5p | [260]  [260]  [259] |
|  | monolayer | ↑ EMT-marker genes (e.g. HAS2, VIM, FZD7, ADAM19, IL-6) | [67] |
|  |  | **ECM** |  |
|  | tissue | ↑ tenascin C | [48] |
|  | tissue | ↑ hyaluronan, ↑ HAS, ↑ HYAL | [24, 156], [210], [210] |
|  | monolayer | ↑ HAS 2, normal HAS 1/3 | [221] |
|  | monolayer | ↑ MMP-1/2/3/9, MT1-MMP | [123]‡ |
|  | tissue | ↑ PAI-1 | [234] |
|  |  | **wound healing mediators** |  |
|  | tissue  monolayer | ↑ CTGF  ↑ CTGF, normal CTGF | [104]  [104], [104] |
|  | tissue  monolayer | ↑ HGF, ↑ c-Met  ↑ HGF, ↑ c-Met | [165]  [165] |
|  | tissue  monolayer | ↑ VEGF  ↑ VEGF, ↑ PLGF | [178]  [178] |
|  | tissue | ↑ TGF-β1 | [2] |
|  | tissue  monolayer | + / ↑ HDGF  normal HDGF | [180]  [180] |
|  | monolayer | ↓ FGF-2 | [166] |
|  | tissue | ↑ TGF-β1 | [2] |
|  | tissue | IGF-1R ↑, normal | [82, 263], [174] |
|  | tissue | ↓ IL-10 | [50] |
|  | tissue | ↑ IL-18, IL-18Rα | [50] |
|  | tissue  monolayer | normal STAT3  normal STAT3, ↓ p-STAT3 (Tyr705) | [127]  [128]‡ |
|  | tissue | ↑ ERK1/2; ↑ Gα1, ↑ Gα3; ↓ Nrf2; ↑ TSLP | [152]; [274]; [121]; [208] |
|  | monolayer | ↓ activin-A (TGF-β superfamily), ↑ follistatin (antagonist) | [164] |

**Supplemental table 3A.** Cellular abnormalities in keloid epidermis – continued

| **Cell type** | **Material** | **Keloid parameters** | **Reference** |
| --- | --- | --- | --- |
| **Keratinocytes** |  | **cytokine array on supernatant** |  |
|  | monolayer | - ↑ OPG, TIMP-2; ↓ GM-CSF, CCL15, IL-8  - normal TIMP-1 | [128]‡ |
|  | monolayer | **hypoxic culture conditions**  - acquire elongated, spindle, fibroblast-like shape  - EMT marker expression: ↑ HIF-1α, vimentin, fibronectin;  ↓ zonula occludens-1, E-cadherin; ↑ migration | [141] |
|  |  | **microarray studies** |  |
|  | monolayer | 15 genes ↑, 16 genes ↓ with > 2.5-fold differential expression  - ↑ wound healing, cellular motility, vascular development,  EMT gene expression  - ↓ transcription factor, cell adhesion, intermediate filament  gene expression | [66] |
|  | monolayer | functional analysis of 523 differentially expressed genes  ↑ HOXA7; ↓ PSMA4, PSMB2, CDK1 | [124] |
|  | tissue | - ↓↓ melanocortin-1 receptor  - ↓ vitamin D receptor | [138]  [68] |
|  |  | **receptors** |  |
|  | tissue | CD34 – | [19] |
|  | tissue  monolayer | c-Kit +, ↑  ↑ c-Kit | [19], [161]  [161] |
|  | tissue | ↓ adiponectin receptor | [139] |
|  |  | **KK + supernatant** |  |
|  | monolayer | + KF-supernatant:  ↑ oncostatin M | [34]* |
|  |  | **various** |  |
|  | tissue | ↑ proopiomelanocortin | [211] |
|  | tissue | ↑ adiponectin | [139] |
|  | tissue | ↑↑ leptin, ↑ S100A12 | [203], [277] |
|  | tissue | ↑ iNOS | [81] |
|  | tissue | - COX-1 (100%), +/– –, +  - COX-2 (87%), ↑, + | [1], [186], [181]  [1], [186], [181] |
|  | tissue | ↑ PAR-1, ↑ PAR-2 | [145] |
|  | tissue | ↑ aldo-keto reductase enzyme AKR1B10 | [98] |
|  | tissue  monolayer | ↑ caspase-1, ↓ caspase-3  ↑ caspase-1 | [50]  [50] |
|  | monolayer | ↑ metallothioneins (MT-1A, MT-1E, MT-1F, MT-2A) | [130] |
|  | monolayer | ↑ HIPK2, HIPK2 activity | [278] |
|  | monolayer | ↓ retinoic acid synthesis | [98] |
| **Melanocytes** | tissue | normal negative HMB-45 expression | [214] |
|  | in vivo | normal melanin volume % | [232] |

**Supplemental table 3A.** This table summarizes the reported intrinsic cellular abnormalities in keloid epidermis, although this includes Langerhans cells, these are listed in supplemental table 3D on immune cell abnormalities. Material definitions: ‘fresh cells’ refers to freshly isolated cells from tissue that are immediately used for experimental analysis; in contrast, ‘monolayer’ refers to in vitro cultured monolayers of cells; ‘tissue’ refers to studies in which the whole biopsy is studied, usually for immunohistochemistry and immunofluorescence, but also for gene expression analysis; in vivo: non-invasive in vivo analysis. Note that we made no distinction between protein or gene expression to avoid compromising readability of the table. Legend; +: present, normal expression or values; ↑: increased; −: absent; ↓: decreased; + / ↑: variable expression, both normal and increased expression observed (within same study). Abbreviations in alphabetical order; ADAM19: a disintegrin and metalloprotease domain 19; α-SMA: alpha smooth muscle actin; Bcl-2: B-cell lymphoma 2; CCL15: C-C motif chemokine ligand 15; CTGF: connective tissue growth factor; COX: cyclo-oxygenase; CDK1: cyclin-dependent kinase 1; ECM: extracellular matrix; EMT: epithelial-mesenchymal transition; ERK1/2: extracellular signal-related kinase 1/2; FGF: fibroblast growth factor; FGFR: fibroblast growth factor receptor; FDZ7: frizzled 7; Gα: G-protein α subunits; GM-CSF: granulocyte-macrophage colony-stimulating factor; HAS: hyaluronan synthase; HDGF: hepatoma-derived growth factor; HIPK2: homeodomain-interacting protein kinase 2; HOXA7: homeobox A7; HYAL: hyaluronidase; IGF-1R: insulin-like growth factor 1 receptor; IL: interleukin; iNOS: inducible nitric oxide synthase; K: keratin; MMP: matrix metalloproteinase; MTT assay: 3-(4,5-dimethylthiazol-2-yl)-2,5-diphenyltetrazolium bromide (cell proliferation assay); NK: normal keratinocytes; Nrf2: nuclear factor erythoid 2-related factor 2; OPG: osteoprotegerin; PAI-1: plasminogen activator inhibitor 1; PAR: protease activated receptor; PLGF: placental growth factor; PSMA4: proteasome subunit alpha type 4; PSMB2: proteasome subunit beta type 2; SC: stratum corneum; SMAD3: mothers against decapentaplegic homolog 3; STAT3: signal transducer and activator of transcription 3; TAp63: p63 isoform; TEWL: transepidermal water loss; TGF-β: transforming growth factor beta; TIMP: tissue inhibitor of metalloproteinase; TSLP: thymic stromal lymphopoietin; VIM: vimentin; VEGF: vascular endothelial growth factor.

**Supplemental table 3B.** Cellular abnormalities in keloid fibroblasts

| **Cell type** | **Material** | **Keloid parameters** | **Reference** |
| --- | --- | --- | --- |
| **Fibroblasts** |  | **morphology** |  |
|  | tissue | ↑ fibroblasts, + / ↑ | [93, 152, 228, 238], [206] |
|  | monolayer | ↑ length, width, cell size, nucleus size of KF  ↑ vacuoles and dense bodies in KF | [11] |
|  |  | normal fibroblast cell volume | [188]* |
|  | monolayer | Kscar tissue is stiff, but  KF are softer with loss of stiffness sensing, and  ↑ responsiveness to dermis-equivalent matrix stiffness | [80]* |
|  | monolayer | ↑ type I fibroblast (migratory, small)  ↑ type II fibroblast (adhesive, large)  epithelial outgrowth −; ↑ mucin | [45]* |
|  | tissue  monolayer | **proliferation**  normal Ki67  normal proliferation, normal in PKF  normal proliferation (10% FBS), (serum −) | [133]  [33, 131, 176, 205, 254] [47, 139, 175, 188]* [23]*‡, [243]  [192], [261]* |
|  | tissue  monolayer | ↑ Ki67  ↑ proliferation, ↑ in CKF  ↑ in proliferative cell cycle phase (G2-M/S) in PKF  ↑ proliferation (low serum), ↑ proliferation (+ serum)  ↑ proliferation to greater number of different cytokines | [6, 7, 95]  [28, 44, 96, 100, 127, 128, 151, 176, 225, 248, 255]  [8, 35, 36, 63, 65, 70, 71, 75, 185, 245, 269]*, [140]*  [236]*  [192], [261]*  [128]‡ |
|  | monolayer | ↓ proliferation, ↓ in CKF  ↓ regardless of medium/density | [45]* [149], [243]  [150]*‡ |
|  | monolayer | normal density-dependent growth inhibition | [188]* |
|  | monolayer | minimal ↓ proliferative capacity with cell aging,  but ↓ cell density results in earlier ↓ proliferation | [149] |
|  | monolayer | **senescence, telomere dysfunction**  normal lifespan | [190]* |
|  | tissue  monolayer | ↑ p16  ↑ senescence | [133]  [243] |
|  | monolayer  tissue | ↓ telomere length, ↑ ROS  ↓ telomere length, normal absence of telomerase activity | [56]  [56] |
|  | monolayer | ↑ telomerase, ↑ telomerase activity  ↑ telomere length, ↑ β-catenin | [266, 273]  [266] |
|  | monolayer | **apoptosis**  normal apoptosis  normal ceramide-induced apoptosis  normal caspase-3, caspase-9 (serum +) | [41]  [137]*  [6, 7] |
|  | monolayer | ↓ apoptosis  ↓ apoptosis genes (Fas, FLICE, RIP, FAF, FAP)  ↑ apoptosis-resistance (Fas-mediated)  ↑ apoptosis-resistance (C2-ceramide-induced)  ↑ apoptosis-inhibitor c-IAP-1, AVEN (in PKF) | [60, 112, 140, 236, 245]* [40, 155]  [155]  [137]*  [174]  [154], [202] |
|  | tissue  monolayer  tissue  monolayer | ↑ apoptosis  ↑ apoptosis (serum −)  ↑ apoptosis-inducer ADAM12 in CsKF  ↑ caspase-1, caspase-3, ↑ caspase-2, ↑ caspase-9  ↑ caspase-3, ↑ caspase-9 (serum −)  normal caspase-3 activity | [6, 7]  [6, 7]  [202]  [6, 7, 50], [6, 7, 50], [6, 7], [6, 7]  [6, 7]  [60]* |
|  | tissue  monolayer | ↑ p53 in PKF, p53 +/–  ↑ p53, ↑ p53 gene mutations; ↓ p53, normal p53 | [112], [76]  [112]*, [193]*; [40], [101] |
|  | tissue | ↑ Fas in CKF | [112] |
|  | tissue  monolayer | ↑ Bcl-2 in PKF  ↑ Bcl-2, ↓ Bcl-2, normal Bcl-2 | [112]  [112]* [96], [101], [137]* |
|  | monolayer | ↓ Bax, ↑ Bax:Bcl-2 ratio (apoptosis susceptibility) | [101] |
|  | tissue  monolayer | ↑ Bcl-x in PKF  − Bcl-x | [112]  [112]* |

**Supplemental table 3B.** Cellular abnormalities in keloid fibroblasts – continued

| **Cell type** | **Material** | **Keloid parameters** | **Reference** |
| --- | --- | --- | --- |
| **Fibroblasts** | tissue | **apoptosis – continued**  ↑ PML in PKF, normal PML in CKF | [243] |
|  | monolayer | ↓ p21, ↑ p27 | [101], [91] |
|  | monolayer | **migration**  normal migration | [139]* |
|  |  | ↑ migration | [55, 99, 127, 225, 248]  [58, 80, 245, 247]* |
|  |  | ↓ wound closure rate | [172] |
|  | tissue | ↑ RUNX2, ↓ caveolin-1 | [80] |
|  | monolayer | ↑ RUNX2 | [80]* |
|  | monolayer | **metabolism**  ↑ metabolic activity | [151] |
|  | monolayer | - ↑ glycolysis and glycolytic enzymes  - ↑ ATP biosynthesis  - ↑ glucose consumption, ↑ lactate accumulation | [244] |
|  | monolayer | **ECM components**  ↑ collagen | [176] |
|  | monolayer | normal collagen I | [139]* [132, 240] |
|  | tissue | ↑ collagen I | [95, 275] |
|  | monolayer | ↑ collagen I | [40, 49, 55, 81, 118, 131, 136, 139, 200, 218, 240, 253] [46, 57, 72, 80, 269]* [9]‡ [150]*‡ |
|  |  | ↑ collagen I in PKF, ↑ collagen I in 44% of cases | [224], [4] |
|  |  | ↓ collagen I | [129] |
|  | monolayer | ↑ collagen II | [131] [257]* |
|  | monolayer | normal collagen III | [43]* [240] |
|  |  | ↓ collagen III | [132, 136] |
|  |  | ↑ collagen III, ↑ collagen III in PKF | [118, 129, 131, 200] [80, 269]* [9]‡, [224] |
|  | monolayer | ↑ collagen I:III ratio | [3, 118, 240] [57, 269]* |
|  | monolayer | ↑ collagen IX, XI, VIII, XVII, IX; XI | [257]*; [80] [257]* |
|  |  | normal collagen V, normal collagen XIV | [129], [48]* |
|  | monolayer | normal fibronectin | [48, 49] [48]* [23]*‡ |
|  | tissue  monolayer | ↑ fibronectin  ↑ fibronectin | [176, 209] [125]*  [17, 28, 41, 80, 107, 125, 139, 209, 218]* [55] [108]*‡ |
|  | monolayer | ↓ fibronectin | [176] |
|  | monolayer | normal elastin, normal elastin fiber-required factors | [85]* |
|  |  | ↑ elastin | [118] [189, 191]* |
|  | tissue | ↑ hyaluronic acid  + serum: ↑ hyaluronic acid | [257]  [156]* |
|  | monolayer | ↓ hyaluronic acid | [156]* |
|  |  | ↑ hyaluronan and proteoglycan link protein 1/2 | [217] |
|  | monolayer | ↑ glycosaminoglycans | [23]*‡ |
|  | tissue  monolayer | normal decorin, + serum: ↓ decorin  ↓ decorin | [166, 227], [166]  [152] [227]* |
|  | tissue  monolayer | normal biglycan  normal biglycan | [227]  [227] |
|  | tissue  monolayer | ↑ versican  normal versican, ↑ versican | [257]  [227], [257]* |
|  | monolayer | ↑ aggrecan | [257]* |
|  | monolayer | ↑ lumican | [257]* |
|  | monolayer | normal syndecan, + serum: normal ↑ syndecan | [166] |
|  | monolayer | ↑ proteoglycan 1 | [202] |
|  | monolayer | ↑ periostin | [142]* |
|  | monolayer | ↑ tenascin C | [47, 48]* [48] |
|  | monolayer | ↓ dermatopontin | [190]* |
|  | monolayer | ↑ thrombospondin  ↑ THBS-1, ↑ THBS-5  ↓ THBS-2 in PKF | [257]*  [40], [87]*  [202] |
|  | monolayer | ↓ desmin; ↑ HSP27; ↑ Sp1 | [47]*; [28, 217, 218], [163] |

**Supplemental table 3B.** Cellular abnormalities in keloid fibroblasts – continued

| **Cell type** | **Material** | **Keloid parameters** | **Reference** |
| --- | --- | --- | --- |
| **Fibroblasts** |  | **ECM receptors** |  |
|  | monolayer | ↑ α2β1 integrin collagen receptor | [28, 217, 218, 226] |
|  | fresh cells  tissue | ↑ α1β1 integrin collagen receptor  ↑ α1β1 integrin collagen receptor | [226]  [226] |
|  |  | ↑ α5β1 integrin collagen receptor  ↑ αVβ3 integrin collagen receptor | [226]  [158] |
|  | monolayer | ↑ fibronectin receptor | [17]* |
|  | monolayer | **ECM synthesis/deposition**  normal collagen synthesis | [190]* [183] |
|  |  | normal proα2(I) collagen gene copy numbers | [240] |
|  |  | collagen synthesis ↑ in 56%, normal in 44% of cases | [4] |
|  |  | ↑ collagen synthesis | [17, 58, 75]* [3, 149, 176, 223] [23]*‡ [9]‡ |
|  | monolayer | ↑ iNOS and NOS activity | [81] |
|  | monolayer | ↑ hyaluronan synthase, normal | [202], [210] |
|  | tissue  monolayer | ↑ hyaluronan synthase  ↑ hyaluronic acid synthesis | [210]  [10] |
|  | tissue  monolayer | ↑ PAI-1  ↑ PAI-1, ↑ PAI-2 | [234]  [150]*‡ [234, 235]* [55, 78, 218, 271], [150]*‡ [217, 218] |
|  | tissue  monolayer | ↓ uPA, ↑ uPAR  ↓ uPA | [234], [114]  [234, 235]* |
|  | monolayer | ↑ lysine hydroxylation of collagen I (crosslinking) | [242]*‡ |
|  | monolayer | ↑ prolyl hydroxylase activity in 50% (collagen stability)  ↑ hydroxyproline (collagen stability) | [4]  [55] |
|  | monolayer | **ECM degradation**  normal collagen degradation | [17]* [4] |
|  |  | ↓ collagen degradation in 33% of samples | [4] |
|  | monolayer  tissue | normal collagenase  ↓ collagenase  ↑ cathepsin K | [4]  [23]*‡  [187] |
|  | monolayer | ↓ MMP-1, normal absent MMP-1  ↓ MMP-2, ↓ MMP-8, ↓ MMP-13 | [150]*‡ [237, 261]*, [31]  [262]*, [237]*, [217] |
|  |  | ↓ MMP-3  ↓ MMP-3: ↓ in CsKF, ↓↓ in CdKF/PKF | [150]*‡ [202, 217] [190]*  [237]* [202] |
|  |  | ↑ MMP-1, ↑ MMP-2, ↑ MMP-3, ↑ MMP-9  ↑ MMP-13, ↑ MT1-MMP  ↑ MMP-19, ↑ MMP-19 in PKF | [58]* [123]‡, [58, 86]* [123]‡ [31], [123]‡ [31], [123]‡,  [237]* [31], [123]‡  [202, 218] |
|  | tissue | normal MMP-1, ↑ MMP-2, normal MMP-9 | [86] |
|  | monolayer | ↓ TIMP-2, ↑ TIMP-1 | [51, 262]*, [58]* [31] |
|  | tissue | normal absence of TIMP-1/2/3 | [86] |
|  | monolayer  tissue | normal hyaluronidase  ↑ hyaluronidase | [210]  [210] |
|  | tissue | **wound healing mediators**  ↑ TGF-β | [158, 201] |
|  | monolayer | ↑ TGF-β1 | [29, 31, 53, 55, 101, 129, 136, 218, 254] [35, 36, 59, 60, 71, 148, 157, 245]* |
|  |  | ↑ TGF-β2, normal TGF-β2 | [31, 55, 218, 252], [29, 101] |
|  |  | ↑ TGF-β3, normal TGF-β3, ↓ TGF-β3 | [101], [218], [29, 31] |
|  |  | ↑ TGFβRI, normal TGFβRI  ↑ TGFβRII, normal TGFβRII, ↓ TGFβRII | [233, 254] [39]*, [31]  [233] [39]*, [41], [31] |
|  | monolayer | normal SMAD2, ↑ SMAD 2 | [267]*, [129, 233, 254] [39]* |
|  |  | ↑ SMAD3, ↓ SMAD 3 | [233] [39]*, [129, 233] [267]* |
|  |  | normal SMAD 4, ↑ SMAD4 | [233] [267]*, [129] |
|  |  | ↓ SMAD 6 | [233] [267]* |
|  |  | ↓ SMAD 7; ↓ / + SIP1 | [53, 129, 233] [267]*; [275] |
|  |  | ↑ CTGF | [55, 101, 104, 253, 254] [104, 190]* |

**Supplemental table 3B.** Cellular abnormalities in keloid fibroblasts – continued

| **Cell type** | **Material** | **Keloid parameters** | **Reference** |
| --- | --- | --- | --- |
| **Fibroblasts** |  | **wound healing mediators** – continued |  |
|  | monolayer | ↑ KGF | [34]* |
|  | monolayer | normal PDGF, normal low PDGF-α/β | [263]*, [153] |
|  | tissue | ↑ PDGF | [158] |
|  | monolayer | normal PDGFRα; ↑ PDGFRβ, ↓ PDGFRβ | [153] [69]*; [153], [158] |
|  | monolayer  tissue | normal HGF; + serum: ↑ HGF  ↑ HGF | [95]* [165]; [165]  [95] |
|  | monolayer  tissue  monolayer | normal c-Met, + serum: normal ↑ c-Met  ↑ c-Met, p-Met  ↑ Met, p-Met | [165]  [95]*  [95]* |
|  | monolayer | ↑ HDGF | [180] |
|  | monolayer | normal bFGF, ↓ bFGF | [71, 148]*, [35, 36, 173]* |
|  | monolayer | normal FGF-2 | [166] |
|  | tissue | ↑ VEGF | [158] |
|  | monolayer | ↑ VEGF, ↑ VEGF in CKF, PDGF − | [49, 178, 251] [59]*, [65]*, [178] |
|  | tissue | ↑ EGF, ↑ EGFR | [158] |
|  | tissue | normal FGF-1, ↑ FGF-2; ↓ FGFR | [158]; [158] |
|  | tissue | ↓ TNF-α, ↑ TNF-β | [154, 158], [158] |
|  | monolayer | ↑ TNF-α, TNF-receptor-associated factors | [154] |
|  | tissue | ↑ CXCL1, CXCR2 | [172] |
|  | monolayer | normal CXCL1, CXCR2 | [172]* |
|  | tissue | ↑ MCP-1, CCR2 | [126] |
|  | monolayer | ↓ MCP-1 | [262]* |
|  |  | ↑ MCP-1; ↑↑↑ CdKF, ↑↑ CsKF, ↑ PKF | [202]; [202] |
|  | tissue | ↑ IL-6, ↑ IL-6R; ↑ IL-6 downstream signalling targets | [158, 273] [63]*; [158] [63]* |
|  | monolayer | ↑ IL-6, ↑ IL-6 in CKF  downstream IL-6 signalling targets (e.g. JAK1, STAT3) | [63, 256]* [50], [49]  [65]* [63]* |
|  | tissue | ↑ IL-18, IL-18Rα | [50] |
|  | monolayer | ↑ IL-8, IL-10; IL-18 − | [50] |
|  | tissue | normal IGF-1 absence, ↑ IGF-1R | [158], [82, 174, 263] |
|  | monolayer | normal IGF-1, ↑ IGF-1R | [182], [82, 174, 182] [263]* |
|  | monolayer | ↑ IGF-binding and IGF-binding-related proteins (e.g. IGFBP 2/5/7, STAT1),  ↑ IGFBP5, ↑ IGFBP5 only in active areas  ↑ IGFBP2, IGFBP4; ↓ IGFBP3  ↑ STAT1 | [213]*  [190]* [182, 202], [190]  [182]; [182]  [202] |
|  | tissue  monolayer | ↑ STAT3  ↑ STAT3, ↓ p-STAT3 (Tyr705) | [127, 202]  [128]‡ |
|  | tissue | ↑ JAG1, Notch-1/2 | [225] |
|  | monolayer | ↑ JAG1, Notch-1/2 | [225] [190]*, [225] |
|  | monolayer | ↑ JAK2 | [127] |
|  | tissue | ↑ ERK phosphorylation | [105] |
|  | monolayer | ↑ ERK1, ERK2; ERK phosphorylation  normal PI3K and MEK-ERK pathways | [152]; [105]  [131] |
|  | monolayer | ↑ NF-κB; ↑ NF-κB activity, ↑ NF-κB binding activity | [154]; [143, 154] |
|  | monolayer | normal glucocorticoid receptors levels,  binding specificity and number of binding sites | [61]* |
|  | tissue | ↑ β-catenin | [84, 201] |
|  | tissue  monolayer | ↑ Wnt5a  ↑ Wnt5a, ↓ Wnt pathway inhibitors (e.g. SFRP1) | [84]  [84]*, [213]* |
|  | monolayer | ↑ stem cell factor | [161] |
|  | tissue | ↓ SFRP1, ↓ SFRP2 | [190] |
|  | monolayer | ↑ SFRP1, ↓ SFRP1 | [247]*, [190]* |
|  | monolayer | ↓ prostaglandin E2 | [261]* |
|  | monolayer | ↑ HIF-1α; ↑ FAP-α | [271]; [49] |
|  | monolayer | ↓ adiponectin, adiponectin receptor | [139]* |
|  | monolayer | ↑ activin-A (TGF-β superfamily), follistatin (antagonist) | [164] |
|  | monolayer | ↑ RAC activity | [248] |
|  | monolayer | ↑ GDF-9 in PKF | [62] |
|  | tissue | ↑ HSP47; ↑ Gα1/3; ↑ NICD; ↑ HtrA1; ↑ MIF | [169]; [274]; [106]; [258]; [74] |

**Supplemental table 3B.** Cellular abnormalities in keloid fibroblasts – continued

| **Cell type** | **Material** | **Keloid parameters** | **Reference** |
| --- | --- | --- | --- |
| **Fibroblasts** | monolayer | **microarray studies**  canonical pathway analysis: 345 genes ↑, 86 genes ↓  - the osteoblasts, osteoclasts and chondrocytes as most important  signalling pathway: ↑ RUNX2, NKX3-2, MLXIPL | [80]* |
|  | monolayer | 374 genes ↑, 758 genes ↓  - ↑ involved in ECM, growth factors and their receptors,  transcription factors and proteins binding with DNA,  apoptosis  - ↓ growth factors and their receptors, transcription factors  and proteins binding with DNA, MMP, cellular skeleton  and movement | [87]*‡ |
|  | monolayer | 250 differentially expressed genes  - ↑ collagen I and III, collagen processing genes  - ↑ cancer, cellular movement, cellular growth and  proliferation, tissue development, connective tissue  function, cell death genes  - ↓ cancer, reproductive system disease, tissue  development, cell growth and proliferation, cell-to-cell  signalling genes | [249] |
|  | monolayer | 43 genes ↑, 5 genes ↓  - ↑ annexin A2 (migration), transgelin  - ↑ ribosomal protein S18/10, ribosomal protein L23A  - ↑ tumour-related genes (e.g. TCTP, MRF-15)  - ↑ collagen I, fibronectin, lysyl oxidase (ECM)  - ↑ cytoskeleton-related proteins (β/γ actin, tropomyosin)  - ↑ ASY (pro-apoptotic), PEA15 (anti-apoptotic)  - ↑ IGFBP3 | [198] |
|  | monolayer | 97 genes ↑, 26 genes ↓, e.g.  - ↑ collagens, fibronectin, proteoglycans, P4H, TIMP3, MMP-14  - ↑ INHBA, FAP-α, CTGF, VEGF, TGF-β1, IGFBP5/7;  - ↓ EGFR, TGFβRIII | [202] |
|  | monolayer | 12 genes ↑, 13 genes ↓ with > 2.5-fold differential expression  - ↑ proliferation, migration, cell shape, invasion, EMT  - ↓ endocannabinoid signalling  - both ↑ and ↓ of various genes involved in ECM, cell-cell  signalling, regulation of cell proliferation, organ  morphogenesis, integral to plasma membrane | [66] |
|  | monolayer | functional analysis of 658 differentially expressed genes  - ↓ SMAD2, CDKN1A; ↑ BMP4, HOXA9 | [124] |
|  | monolayer | total RNA sequencing + bioinformatics analysis:  - ↑ pathways for hepatic fibrosis, Wnt, TGF-β, STAT3, EMT,  epithelial adherens junction signalling | [122]‡ |
|  | fresh tissue  monolayer | **multiple genes expression** **analysis**  19 genes selected from bioinformatics analysis:  - ↑ aggrecan, asporin, (ECM)  - ↑ inhibin β A, TNF-α inducible protein 6, HIF-1α, IGF-  binding protein 7, pleiotrophin, galactin-1, serpin  peptidase inhibitor clade H (WHM)  - ↑ chromosome 5 open reading frame 13  - passage 2 KF show normal expression of the above | [207] |
|  | tissue | **epithelial-mesenchymal transition genes**  - normal FGFR2-IIIb, ↑ FGFR-IIIc, normal absent TAp63  - ↑ snail 1, ↑ snail 2 | [260] |
|  | monolayer | **epigenetic alterations**  - hypermethylation of genes in the homeotic (HOX)A  cluster 🡪 correlated with ↓ HOXA9, HOXA10  - hypomethylation of asporin, thrombin-like receptor and  MMP-3 🡪 correlated with ↑ asporin, thrombin-like  receptor; ↓ MMP-3 | [190]* |
|  |  | - ↑ DNMT1, ↑ TGF-β1, ↓ SMAD 7 🡪 reversible by  DNA methylation inhibition | [53] |

**Supplemental table 3B.** Cellular abnormalities in keloid fibroblasts – continued

| **Cell type** | **Material** | **Keloid parameters** | **Reference** |
| --- | --- | --- | --- |
| **Fibroblasts** | monolayer | **proteomic profiling**  16 differentially expressed proteins  - ↑↑ HSP70-9B, HSP70-1L, vimentin, dihydropyrimidinase-  like 2; ↑ crocalbin-like protein, tropomyosin 1, HSP70-1A | [116] |
|  |  | 21 biomarker proteins selected from previous microarray:  - ↑ MMP-19, PAI-2; collagen I, fibronectin 1, α-SMA,  α2β1 integrin, vinculin; TGF-β1, TGF-β2; normal TGF-β3 | [218] |
|  |  | **↑ sensitivity of KF to soluble mediators/stimulation** |  |
|  | monolayer | KF + IL-18:  - ↑ collagen secretion (NF −) | [50] |
|  |  | KF + HDGF:  - ↑ proliferation (NF −) | [180] |
|  |  | KF + VEGF:  - ↑ PAI-1 (NF −) | [251] |
|  |  | KF + CTGF:  - ↑ proliferation, migration, ECM synthesis (NF −) | [139]* |
|  |  | KF + TGF-β1:  - ↑ PDGFα receptor (NF −) | [153] |
|  |  | KF + BMP4:  - reprogramming to adipocyte (HsF −)* | [184] |
|  |  | KF + TGF-β:  - ↑↑ TGFβRII; ↑↑ DNA synthesis, collagen I and III (NF +) | [39]*; [25, 46]* |
|  |  | KF + HGF:  - ↑↑ migration, invasion, proliferation, collagen I (NF +) | [95]* |
|  |  | KF + PDGF:  - ↑↑ mitogenicity, migration (NF +) | [69]* |
|  |  | KF + IL-18:  - ↑↑ IL-6, IL-8 (NF +) | [50] |
|  |  | KF + MIF:  - ↑↑ arachidonic acid release (NF +) | [74] |
|  |  | KF + NO:  - ↑↑ collagen I (NF +) | [81] |
|  |  | KF + hypoxia:  - ↑↑ PAI-1, ERK1/2 (NF +) | [272] |
|  |  | **KF + supernatant** |  |
|  | monolayer | ↑ IL-18 | [55] |
|  | monolayer | KF + BM-MSC supernatant:  - ↓ cell metabolic activity, proliferation, α-SMA  - ↓ migration, CTGF, PAI-1, TGF-β1/2,  - ↓ collagen I, fibronectin, ↓ hydroxyproline  - ↑ TGF-β3, decorin | [55, 250]  [55]  [55, 250]  [55] |
|  | monolayer | KF + WJ-MSC supernatant:  - ↑ PAI-1, TGF-β2, IL-6, IL-8; ↑ proliferation  - ↓ TGF-β3 | [15]* |
|  | monolayer | KF + KK-supernatant:  - normal ↑ proliferation, normal ↓ apoptosis  - ↑ ERK1/2, JNK1/2 | [60]* |
|  | monolayer | KF + mast cell line (HMB-1) membrane fragments:  - ↑↑ HIF-1α, VEGF | [270] |
|  |  | **effects of KF supernatant** |  |
|  | monolayer | NF + NK/KF supernatant:  - ↑ collagen I, FAP, migration, OSM, p-STAT3 (Tyr705) | [34]* |
|  | monolayer | NF/NsF + KF supernatant:  - ↑ proliferation, migration; collagen I, fibronectin, α-SMA,  PAI-1, TGF-β, CTGF | [16] |
|  | monolayer | NF + KF supernatant: ↑ collagen synthesis | [223] |
|  | monolayer | normal endothelial cells + KF supernatant: ↑ migration | [59]* |
|  |  | **cytokine array on KF secretions** |  |
|  | monolayer | - ↑ IL-6, IL-8, HGF  - ↓ CCL15, Ang, FGF-4/6, IGFBP2, OPG, TGF-β2/3  - normal TIMP-1/2, IGFBP4, MCP-1 | [128]‡ |

**Supplemental table 3B.** Cellular abnormalities in keloid fibroblasts – continued

| **Cell type** | **Material** | **Keloid parameters** | **Reference** |
| --- | --- | --- | --- |
| **Fibroblasts** | monolayer | **3D structures**  CKF formed stratified 3D structure | [65]* |
|  |  | formed 3D dermis-like structure with  continued ↑ collagen I and III (normal levels in monolayer) | [200]‡ |
|  |  | ↑ LH2b, ↑ crosslinking in 3D collagen structure | [212] |
|  | PA gel | ↑ RUNX2, collagen III and XI, fibronectin | [80]* |
|  | PEG-  collagen gel | ↑ collagen I, glycosaminoglycans; normal MMP1/2/3 and MTP-MMP, ↑ MMP9/13 | [123]‡ |
|  | Matrigel ® matrix | ↑ collagen I, glycosaminoglycans; normal MMP1/2/3 and MTP-MMP, normal ↓ MMP9/13 | [123]‡ |
|  | collagen gel | ↑ contraction, ↑ contraction in CKF | [28, 144, 161, 196, 220]  [72, 73, 102, 195, 276]*, [222]* |
|  |  | + serum: ↑↑ contraction | [161] |
|  |  | + KK or KF supernatant: slight ↑ contraction  + KK/KF supernatant: ↑↑ contraction | [162] |
|  |  | + IGF-1: ↑ invasion | [263]* |
|  |  | ↑ α-SMA  + KK/KF supernatant: ↑ α-SMA | [73]*  [162] |
|  |  | partial detachment, self-shrinkage, ↑ α-SMA, normal apoptosis | [41] |
|  |  | + TGF-β1 + adipose stromal cell supernatant:  - ↓ contraction | [216] |
|  |  | ↓ stathmin, normal contraction with high passage (P8)  cells | [199] |
|  |  | ↓ collagen (vs fibrin gel), ↑ uPA, ↑ PAI-1 | [234]* |
|  | fibrin gel | ↑ collagen production, sensitive to TGF-β regulation | [264]* |
|  |  | ↑ collagen, ↑ PAI-1, ↓ uPA | [234]* |
|  |  | no plasmin-mediated fibrinolysis of fibrin gel | [235]* |
|  |  | + TGF-β:  - ↑ contraction; normal contraction without | [265]* |
|  | fibrin-  collagen gel | ↓ collagen (vs fibrin gel) | [234]* |
|  | spheroid | transfected with relaxin-expressing adenovirus:  - ↓ collagen I and III, fibronectin, elastin | [119] |
|  | nano-patterned scaffold | fibrin alignment induces:  - ↓ proliferation, matrix synthesis (collagen I & III, MMP2),  α-SMA (vs flat surface culture)  - smaller, more dendritic/elongated morphology (vs large,  flatter, more spread out morphology in flat surface  culture) | [168]* |
|  |  | **3D invasion assays** |  |
|  | collagen gel | ↑ invasion | [49] |
|  | BM-coating | ↑ invasion | [225] |
|  | Matrigel ® | ↑ invasion  + GA: ↑ invasion | [75]*  [246] |

**Supplemental table 3B.** Cellular abnormalities in keloid fibroblasts – continued

| **Cell type** | **Material** | **Keloid parameters** | **Reference** |
| --- | --- | --- | --- |
| **Fibroblasts** | tissue  monolayer | **mesenchymal cell markers**  vimentin+  vimentin+ | [133]  [95] [142]* |
|  | fresh cells | - ↑ non-haematopoietic MSCs  (CD13+/CD29+/CD44+/CD90+)  - ↓ haematopoietic MSCs (CD34/CD90/CD117) | [89] |
|  | fresh cells  monolayer | MSCs (CD34−/CD73+/CD90+/CD105+) capable of differentiating into adipocytes and osteocytes | [88] |
|  | monolayer | - mesenchymal stem cell markers +  (CD13/CD29/CD44/CD90/fibronectin/vimentin)  - differentiate into neural and mesodermal lineage cells | [159] |
|  | tissue | CD34− | [133] |
|  | tissue | **myofibroblast marker**  α-SMA− | [54, 226, 230] |
|  | monolayer | normal α-SMA | [139]* |
|  | tissue | ↑ α-SMA variable (33-81%) | [12, 103, 117, 160, 197, 208] |
|  |  | ↑ α-SMA | [115, 133] [72]* |
|  | monolayer | ↑ α-SMA | [40, 41, 139, 176, 205, 212, 218] [115]* |
|  | monolayer | ↓ α-SMA | [176] |
|  |  | α-SMA+ in all cultured KF | [95] |
|  | tissue | PAR-1+, PAR-2+ in myofibroblasts | [145] |
| **Fibrocytes** | tissue | ↓ fibrocytes | [238] |
|  | tissue | ↑ CD45+/pro-collagen I+/CXCR4+ | [208] |
|  | tissue  monolayer  fresh cells | ↑ CD45+/collagen I+/CXCR4+  ↑ CD45RO+/25F9+/MRP8/9+  ↑ CD45RO+/25F9+/MRP8/9+ | [88] |
|  | monolayer | - ↑ fibrocyte markers (CD34+/CD86+, N.B. – in NF)  - fibroblast markers + (CD80+/CD14+) | [146] |
|  | monolayer | - ↑ LSP-1+/ collagen type-1+ differentiation (from PBMCs)  - with ↑ resistance against inhibition by SAP | [170] |
|  | plasma | normal levels of fibrocyte inhibitor SAP | [170] |

**Supplemental table 3B.** This table summarizes the most relevant intrinsic cellular abnormalities in keloid fibroblasts. Due to the sheer multitude of available studies on keloid fibroblasts, we focused on those reporting intrinsic abnormalities in the major themes of proliferation, apoptosis, migration, extracellular matrix synthesis/degradation and wound healing mediators. This table does not include dermal cell population (included in supplemental histopathology table 1), as it does not specify the specific type of cell involved. Material definitions: ‘fresh cells’ refers to freshly isolated cells from tissue that are immediately used for experimental analysis; in contrast, ‘monolayer’ refers to in vitro cultured monolayers of cells; ‘tissue’ refers to studies in which the whole biopsy is studied, usually for immunohistochemistry and immunofluorescence, but also for gene expression analysis. Note that we made no distinction between protein or gene expression to avoid compromising readability of the table. Legend; +: present, normal expression or values; ↑: increased; −: absent; ↓: decreased; + / ↑: variable expression, both normal and increased expression observed (within same study); *explant fibroblast cell isolation; unless stated otherwise, fibroblasts were isolated via enzymatic digestion. Abbreviations in alphabetical order; Ang: angiopoietin; α-SMA: alpha smooth muscle actin; ATP: adenosine triphosphate; Bcl-2: B-cell lymphoma 2; Bcl-x: B-cell lymphoma extra large; BM-coating: basement membrane coating; BM-MSC: bone marrow derived mesenchymal stem cells; BMP4: bone morphogenetic protein 4; CCL15: C-C motif chemokine ligand 15; CCR2: C-C chemokine receptor type 2; CDKN1A: cyclin dependent kinase inhibitor 1A; CKF: central keloid fibroblasts; CTGF: connective tissue growth factor; CXCL1: C-X-C motif chemokine ligand 1; CXCR2: C-X-C chemokine receptor type 2; DNMT1: DNA methyltransferase 1; ECM: extracellular matrix; EGF: epidermal growth factor; EGFR: epidermal growth factor receptor; EMT: epithelial-mesenchymal transition; ERK: extracellular signal–regulated kinases (pathway activated by periostin to promote angiogenesis); FAF: Fas-associated factor; FAP: Fas-associated phosphatase or fibroblast activation protein; FAP-α: fibroblast activation protein alpha; FBS: fetal bovine serum; FGF: fibroblast growth factor; FGFR: fibroblast growth factor receptor; FLICE: Fas-associated death domain-like interleukin-1β converting enzyme; GA: gallic acid; GDF-9: growth differentiation factor 9; HDGF: hepatoma-derived growth factor; HGF: hepatocyte growth factor; HIF-1α: hypoxia-inducible factor 1 alpha; HOXA9: homeobox A9; HsF: hypertrophic scar fibroblasts; HSP: heat shock protein; HtrA1: HtrA serine peptidase 1; IGF-1: insulin-like growth factor 1; IGFBP: insulin-like growth factor binding protein; IL: interleukin; IL-6R: interleukin 6 receptor; INHBA: inhibin beta A; iNOS: inducible nitric oxide synthase; JAG1: jagged 1 protein (Notch ligand); JAK2: Janus kinase 2; JNK: c-Jun N-terminal kinases; KF: keloid fibroblast; KGF: keratinocyte growth factor; Kscar: keloid scar; LSP-1: leukocyte specific protein 1; MCP-1: monocyte chemoattractant protein 1; MEK-ERK: mitogen-activated protein kinase-extracellular-signal-regulated kinase; MIF: macrophage migration inhibition factor; MLXIPL: MLX-interacting protein-like; MMP: matrix metalloproteinase; MRF-15: myelin regulatory factor; MSCs: mesenchymal stromal/stem cells; NF: normal fibroblasts; NICD: Notch intracellular domain (involved in cell fate determination, modulates e.g. proliferation, apoptosis, migration); NKX3-2: NK3 homeobox 2; NO: nitric oxide; NOS: nitric oxide synthase; Notch: JAG receptor; NsF: normotrophic scar fibroblasts; OPG: osteoprotegerin; OSM: oncostatin M; P1P: procollagen type 1C peptide (marker for collagen production); P4H: proline-4-hydroxylase (marker for active collagen synthesis); PAR: protease-activated receptor; PAI-1/2: plasminogen activator inhibitor 1/2; PDGF: platelet-derived growth factor; PDGFRα/β: platelet-derived growth factor receptor alpha/beta; PEA15: proliferation and apoptosis adaptor protein 15; PI3K: phosphoinositide 3-kinases; PKF: peripheral keloid fibroblasts; p-Met: phosphorylated Met; PML: promyelocytic leukaemia; p-STAT3 (Tyr705): Tyr phosphorylated (activated) STAT3; RAC: Ras-related C3 botulinum toxin substrate (enzyme); RIP: receptor interacting protein; ROS: reactive oxygen species; RUNX2: Runt-related transcription factor 2 (involved in osteogenesis, chondrogenesis); SIP1: SMAD interacting protein 1 (transcription factor); SFRP1/2: secreted frizzled related protein 1/2; SMAD: mothers against decapentaplegic homolog 1 (Drosophila), major effectors of TGF-β signalling; SP: substance P (neuropeptide); STAT: signal transducer and activator of transcription; TCTP: translationally controlled tumour protein; TGF-β: transforming growth factor beta; TGFβR: transforming growth factor beta receptor; THBS: thrombospondin; TIMP: tissue inhibitor of metalloproteinase; TNF-α/β: tumour necrosis factor alpha/beta; uPAR: urokinase-type plasminogen activator receptor; VEGF: vascular endothelial growth factor; WHM: wound healing mediators; WJ-MSC: human umbilical cord Wharton’s jelly-derived mesenchymal stem cells; Wnt3a: Wnt family member 3a. N.B. any additional information on parameters listed in abbreviations are all derived from cited literature in table.

**Supplemental table 3C.** Cellular abnormalities in keloid neurovasculature

| **Cell type** | **Material** | **Keloid parameters** | **Reference** |
| --- | --- | --- | --- |
| **Endothelial** | tissue | flattening of vascular lumen | [110] |
| **cells** |  | ↑ endothelial cells (vascular density) | [12, 13, 19, 54, 145, 178, 228] |
|  |  | ↓ endothelial cells (vascular density) | [22, 32, 110, 230, 239] |
|  | tissue | ↑ microvascular density | [13] |
|  |  | ↓ microvascular density | [32] |
|  | tissue | vascular density ↓ in centre, ↑ periphery  ↑↑ HIF-1α in centre, ↑ in periphery | [231] |
|  | tissue | ↑ oxidative stress | [121] |
|  | tissue | ↑ CD31/vimentin (EndoMT marker) | [120] |
|  | tissue | normal VEGF  ↑ VEGF | [158, 231]  [231] |
|  | tissue | ↑ endothelin | [109] |
|  | tissue | normal IGF-1, ↑ IGF-1R | [158], [263] |
|  | tissue | - ↓ PDGF, IL-1β, FGF-1, FGF-2, PDGFRβ, IL-6R  - normal absence TGF-β, TNF-β, TNF-α, VEGF, IL-6  - normal EGF, EGFR, FGFR, αVβ3 integrin | [158] |
|  | tissue | ↑ NICD | [106] |
|  | blood | ↑ circulating endothelial progenitor cells (CD45−/CD34+/CD133+/VEGFR2+) | [268] |
| **Nerve cells** | tissue | - ↓ nerve fiber density (PGP 9.5)  - ↑ α1-adrenoceptor/PGP 9.5+ nerve fibers  - ↑ S100+ nerve fibers | [194]  [52]  [79] |
|  | tissue | - nerve fibers (S100+) thinner and longer  - ↑ depth of most superficial nerve fibers | [79] |
| **Whole biopsy** |  | **wound healing mediators** |  |
|  | tissue | ↑ mTOR, phospho-mTOR and downstream targets | [176] |
|  |  | ↑ VEGF, PLGF | [178, 251], [178] |
|  |  | ↑ TNF-α convertase, stem cell factor, c-Kit | [161] |
|  |  | ↑ CTGF | [104] |
|  |  | ↑ Met, p-Met  normal c-Met, ↑  normal HGF, ↑ | [95]  [95], [165]  [95], [165] |
|  |  | ↑ IL-6, IL-17, CXCL1, IL-1β, CCL15, CCL5, SCF,  TGF-β1, TNF-α, angiogenin II, VEGF, PDGF-BB | [273] |
|  |  | ↑ IL-18, IL-18Rα/β, caspase-1 | [55] |
|  |  | angiotensin II +, ↑ AT_1_ receptor | [171] |
|  |  | ↑ oncostatin M | [34] |
|  |  | **ECM-related** |  |
|  | tissue | ↑ HIF-1α, PAI-1 | [271] |
|  |  | ↑ collagen I, FAP | [34] |
|  |  | normal collagen III | [43] |
|  |  | ↑ chondroitin sulphate, hyaluronic acid, keratan sulphate | [85] |
|  |  | ↑ Sp1 | [163] |
|  |  | - ↑ POSTN, PXDN, ADAM19, LOXL2, CDH11  - ↓ HOXC11, HOXA7, CNR1, TGFBR3 | [66] |

**Supplemental table 3C.** This table summarizes the reported intrinsic cellular abnormalities in keloid endothelial cells, nerve cells and tissue extracts from whole biopsies. Material definitions: ‘fresh cells’ refers to freshly isolated cells from tissue that are immediately used for experimental analysis; in contrast, ‘monolayer’ refers to in vitro cultured monolayers of cells; ‘tissue’ refers to studies in which the whole biopsy is studied, usually for immunohistochemistry and immunofluorescence, but also for gene expression analysis. Note that we made no distinction between protein or gene expression to avoid compromising readability of the table. Legend; +: present, normal expression or values; ↑: increased; −: absent; ↓: decreased; ‡: n=1 keloid. Abbreviations in alphabetical order; ADAM19: ADAM metallopeptidase domain 19; CCL: C-C motif chemokine ligand; CDH11: cadherin 11, type 2, OB-cadherin (osteoblast); CNR1: cannabinoid receptor 1 (brain); CTGF: connective tissue growth factor; CXCL1: C-X-C motif chemokine ligand 1; EGF: epidermal growth factor; EndoMT: endothelial-mesenchymal transition; EGFR: epidermal growth factor receptor; FAP: fibroblast activation protein; FGF: fibroblast growth factor; FGFR: fibroblast growth factor receptor; HGF: hepatocyte growth factor; HIF-1α: hypoxia-inducible factor 1 alpha; HOXA7: homeobox A7; HOXC11: homeobox C11; IGF: insulin-like growth factor; IGF-1R: insulin-like growth factor 1 receptor; IL: interleukin; IL-6R: interleukin 6 receptor; IL-18Rα/β: interleukin 18 receptor alpha or beta; LOXL2: lysyl oxidase-like 2; mTOR: mechanistic target of rapamycin; NICD: Notch intracellular domain (involved in cell fate determination, modulates e.g. proliferation, apoptosis, migration); PAI-1: plasminogen activator inhibitor 1; PDGF: platelet-derived growth factor; PDGFR-β: platelet-derived growth factor receptor beta; PLGF: placental growth factor; p-Met: phosphorylated Met; POSTN: periostin; PXDN: peroxidasin homolog (Drosophila); SCF: stem cell factor; Sp1: transcription factor, regulates several ECM promoters; TGFBR3: TGF-β receptor 3; TNF-α/β: tumour necrosis factor alpha/beta; VEGF: vascular endothelial growth factor. N.B. any additional information on parameters listed in abbreviations are all derived from cited literature in table.

**Supplemental table 3D.** Cellular abnormalities in keloid immune cells and serum

| **Cell type** | **Material** | **Keloid parameters** | **Reference** |
| --- | --- | --- | --- |
| **Infiltrate** | tissue | - ↑ inflammatory cell infiltrate  - few immune cells | [12, 92, 204, 206, 228]  [14] |
|  |  | presence of distinctive lymphoid aggregates (15%) | [18] |
| **PBMCs** | monolayer | - ↑ IL-6, TNF-α, IFN-β  - ↓ IFN-α, IFN-γ, TNF-β  - normal Il-1 and IL-2 | [147] |
| **LCs** | tissue  tissue | normal quantities  ↑ CD1a+ | [18]  [92] |
| **Dendritic cells** | tissue | ↑ CD1a+/CD36+/HLA-DR+/ICAM-1+ | [197] |
|  |  | ↑ HLA-DR+, CD1a+, FXIIIa+ | [37, 92, 179] |
| **Macrophages** | tissue | CD68 − | [5] |
|  | tissue | ↑ macrophages | [30, 92, 204] |
|  | fresh cells | - ↑ CD14+  - express ↑ M1 (IL-12, iNOS) & M2 (IL-10, TGF-β) factors | [94] |
|  | tissue | ↑ M2 (CD68+/CD163+) | [18] |
|  | monolayer | + T-cells (from peripheral blood):  - ↑ FOXP3 expression in T-cells | [94] |
| **Mononuclear** | tissue | ↑ proopiomelanocortin | [211] |
| **cells** | monolayer | monocytes + NF:  - ↑ proliferation, MCP-1 secretion in NF | [126]* |
|  |  | monocytes + KK/KF (or NK/NF) model:  - ↑ M2 macrophage differentiation | [135] |
|  | blood | circulating lymphocytes + known T-cell mitogens:  - ↓ mitogenic response | [26] |
|  | blood | circulating lymphocytes:  - antinuclear antibodies against fibroblasts + | [90] |
| **T-lymphocytes** | tissue | ↑ CD3+ | [92, 204] |
|  |  | ↑ CD4+ | [167] |
|  |  | ↑ CD4+:CD8+ ratio | [18, 30] |
|  |  | ↑ CD3+/CD4+/CD8+ | [18, 30] |
|  |  | ↑ CD3+/CD45RO+/CD4+/HLA-DR+/LFA-1+ | [197] |
|  | fresh cells | ↑ CD8+/CD45RO+ memory T-cells, which  produce ↑ IFN-γ and ↓ TNF-α | [38] |
|  | fresh cells | CD8−/CD45RO+/FOXP3+ memory T-cells:  ↓ CD25, CTLA-4 expression; ↓ IL-10 production | [38] |
|  | fresh cells | CD8−/CD45RO+/FOXP3− memory T-cells:  ↑ IFN-γ; ↓ TNF-α, IL-13 production  - ↑ Th1-like with ↓ Th2-like CD8−/CD45RO+/FOXP3− | [38] |
|  | fresh cells | ↑ CD8+/CD103+ memory T-cells | [38] |
|  | fresh cells  tissue | ↑ FOXP3+/CD4+ regulatory T-cells  ↑ FOXP3+/CD4+ regulatory T-cells | [94]  [167] |
|  | blood | circulating CD45RO+ memory T-cells show:  - normal cell quantities  - normal cytokine production profiles | [38] |
|  | blood | ↓ CD4+/CD25^high^/FOXP3+ regulatory T-cells in patients with multiple keloid scars | [38] |
|  | blood | circulating CD3+ T-cells < keloid tissue | [94] |
| **B-lymphocytes** | tissue | ↑ CD20+ in dermis, − in epidermis | [18, 92] |
|  |  | few CD20+; CD20 − | [204]; [18] |
| **Mast cells** | tissue | normal quantities | [14, 21] |
|  |  | ↑ mast cells  ↑ tryptase+ mast cells | [18, 103, 160, 204]  [13, 144] |
|  |  | ↑ mast cells in 36-50%, normal in 27% | [206] [103]‡ ^,^ [103]‡ |
|  |  | ↓ mast cells | [77, 204] |
| **Antibodies** | tissue | ↑ IgA, IgM, C3, C1q deposits | [92] |
| **Serum/Plasma** | serum | ↑ IgM, C3; ↓ IgA, C4 | [26] |
|  |  | ↑ HLA-B14 & HLA-Bw16 antigens | [113] |
|  |  | ↑ anti-hnRNPA2B1 autoantibody | [92] |
|  |  | normal MMP-1/2/9, TIMP-1/2 | [241] |
|  |  | ↑ KGF | [34]* |
|  | plasma | ↑ VEGF | [268] |

**Supplemental table 3D.** This table summarizes the reported intrinsic cellular abnormalities in keloid immune cells and keloid patients’ serum. Material definitions: ‘fresh cells’ refers to freshly isolated cells from tissue that are immediately used for experimental analysis; in contrast, ‘monolayer’ refers to in vitro cultured monolayers of cells; ‘tissue’ refers to studies in which the whole biopsy is studied, usually for immunohistochemistry and immunofluorescence, but also for gene expression analysis. Note that we made no distinction between protein or gene expression to avoid compromising readability of the table. Legend; +: present, normal expression or values; ↑: increased; −: absent; ↓: decreased; ‡: n=1 keloid. Abbreviations in alphabetical order; C1q/3/4: complement component 1q/3/4; CTLA-4: cytotoxic T-lymphocyte–associated antigen 4; FXIIIa: factor XIIIa; FOXP3: forkhead box P3; HLA: human leukocyte antigen; ICAM-1: intercellular adhesion molecule 1; IFN: interferon; IgA/M: immunoglobulin A/M; IL: interleukin; iNOS: inducible nitric oxide synthase; KK/KF model: keloid keratinocyte/keloid fibroblast full thickness *in vitro* model; LFA-1: lymphocyte function-associated antigen 1; M1: classically activated pro-inflammatory macrophage subtype; M2: alternatively activated, pro-fibrotic macrophage subtype; MCP-1: monocyte chemoattractant protein 1; MMP: matrix metalloproteinase; NK/NF model: normal keratinocyte/normal fibroblast full thickness *in vitro* model; TGF-β: transforming growth factor beta; Th1/2: T-helper cells 1/2; TIMP: tissue inhibitor of metalloproteinase; TNF-α/β: tumour necrosis factor alpha/beta; VEGF: vascular endothelial growth factor.

**References**

1. Abdou AG, Maraee AH, Saif HFA-E (2014) Immunohistochemical evaluation of COX-1 and COX-2 expression in keloid and hypertrophic scar. Am J Dermatopathol 36:311–317

2. Abdou AG, Maraee AH, Al-Bara AM, Diab WM (2011) Immunohistochemical expression of TGF-β1 in keloids and hypertrophic scars. Am J Dermatopathol 33:84–91

3. Abergel RP, Chu ML, Bauer EA, Uitto J (1987) Regulation of collagen gene expression in cutaneous diseases with dermal fibrosis: evidence for pretranslational control. J Invest Dermatol 88:727–731

4. Abergel RP, Pizzurro D, Meeker CA, et al (1985) Biochemical composition of connective tissue in keloids and analysis of collagen metabolism in keloid fibroblast cultures. J Invest Dermatol 84:384–390

5. Aiba S, Tabata N, Ishii H, et al (1992) Dermatofibrosarcoma protuberans is a unique fibrohistiocytic tumour expressing CD34. Br J Dermatol 127:79–84

6. Akasaka Y, Ishikawa Y, Ichiro O, et al (2000) Enhanced expression of caspase-3 in hypertrophic scars and keloid: induction of caspase-3 and apoptosis in keloid fibroblasts in vitro. Lab Investig 80:345–357

7. Akasaka Y, Ito K, Fujita K, et al (2005) Activated caspase expression and apoptosis increase in keloids: cytochrome c release and caspase-9 activation during the apoptosis of keloid fibroblast lines. Wound Repair Regen 13:373–382

8. Akino K, Akita S, Yakabe A, et al (2008) Human mesenchymal stem cells may be involved in keloid pathogenesis. Int J Dermatol 47:1112–1117

9. Ala-Kokko L, Rintala A, Savolainen ER (1987) Collagen gene expression in keloids: analysis of collagen metabolism and type I, III, IV, and V procollagen mRNAs in keloid tissue and keloid fibroblast cultures. J. Invest. Dermatol. 89:238–244

10. Alaish SM, Yager DR, Diegelmann RF, Cohen IK (1995) Hyaluronic acid metabolism in keloid fibroblasts. J Pediatr Surg 30:949–952

11. Ali SS, Hajrah NH, Ayuob NN, et al (2010) Morphological and morphometric study of cultured fibroblast from treated and untreated abnormal scar. Saudi Med J 31:874–881

12. Amadeu T, Braune A, Mandarim-de-Lacerda C, et al (2003) Vascularization pattern in hypertrophic scars and keloids: a stereological analysis. Pathol Res Pract 199:469–473

13. Ammendola M, Zuccalà V, Patruno R, et al (2013) Tryptase-positive mast cells and angiogenesis in keloids: a new possible post-surgical target for prevention. Updates Surg 65:53–57

14. Appleton I, Brown NJ, Willoughby DA (1996) Apoptosis, necrosis, and proliferation: possible implications in the etiology of keloids. Am J Pathol 149:1441–1447

15. Arno AI, Amini-Nik S, Blit PH, et al (2014) Effect of human Wharton’s jelly mesenchymal stem cell paracrine signaling on keloid fibroblasts. Stem Cells Transl Med 3:299–307

16. Ashcroft KJ, Syed F, Bayat A (2013) Site-specific keloid fibroblasts alter the behaviour of normal skin and normal scar fibroblasts through paracrine signalling. PLoS One 8:e75600

17. Babu M, Diegelmann R, Oliver N (1989) Fibronectin is overproduced by keloid fibroblasts during abnormal wound healing. Mol Cell Biol 9:1642–50

18. Bagabir R, Byers RJ, Chaudhry IH, et al (2012) Site-specific immunophenotyping of keloid disease demonstrates immune upregulation and the presence of lymphoid aggregates. Br J Dermatol 167:1053–1066

19. Bakry OA, Samaka RM, Basha MA, et al (2014) Hematopoietic stem cells: do they have a role in keloid pathogenesis? Ultrastruct Pathol 38:55–65

20. Beausang E, Floyd H, Dunn KW, et al (1998) A new quantitative scale for clinical scar assessment. Plast. Reconstr. Surg. 102:1954–1961

21. Beer TW, Baldwin H, West L, et al (1998) Mast cells in pathological and surgical scars. Br J Opthalmology 82:691–694

22. Beer TW, Baldwin H, Goddard JR, et al (1998) Angiogenesis in pathological and surgical scars. Hum Pathol 29:1273–1278

23. Berman B, Duncan MR (1989) Short-term keloid treatment in vivo with human interferon alfa-2b results in a selective and persistent normalization of keloidal fibroblast collagen, glycosaminoglycan, and collagenase production in vitro. J Am Acad Dermatol 21:694–702

24. Bertheim U, Hellström S (1994) The distribution of hyaluronan in human skin and mature, hypertrophic and keloid scars. Br J Plast Surg 47:483–489

25. Bettinger DA, Yager DR, Diegelmann RF, Cohen IK (1996) The effect of TGF-β on keloid fibroblast proliferation and collagen synthesis. Plast. Reconstr. Surg. 98:827–833

26. Bloch EF, Hall MC, Denson MJ, Slay-Solomon V (1984) General immunoreactivity in keloid patients. Plast Reconstr Surg 73:448–451

27. Bloor BK, Tidman N, Leigh IM, et al (2003) Expression of keratin K2e in cutaneous and oral lesions: association with keratinocyte activation, proliferation, and keratinization. Am J Pathol 162:963–975

28. Blume-Peytavi U, Geilen CC, Sommer C, et al (1997) The phospholipid analogue hexadecylphosphocholine (HePC) inhibits proliferation of keloid fibroblasts in vitro and modulates their fibronectin and integrin synthesis. Arch Dermatol Res 289:164–169

29. Bock O, Yu H, Zitron S, et al (2005) Studies of transforming growth factors beta 1-3 and their receptors I and II in fibroblast of keloids and hypertrophic scars. Acta Derm Venereol 85:216–220

30. Boyce DE, Ciampolini J, Ruge F, et al (2001) Inflammatory cell subpopulations in keloid scars. Br J Plast Surg 54:511–516

31. Bran GM, Goessler UR, Baftiri A, et al (2010) Effect of transforming growth factor-β1 antisense oligonucleotides on matrix metalloproteinases and their inhibitors in keloid fibroblasts. Otolaryngol - Head Neck Surg 143:66–71

32. Bux S, Madaree A (2010) Keloids show regional distribution of proliferative and degenerate connective tissue elements. Cells Tissues Organs 191:213–234

33. Calderon M, Lawrence WT, Banes AJ (1996) Increased proliferation in keloid fibroblasts wounded in vitro. J Surg Res 61:343–347

34. Canady J, Arndt S, Karrer S, Bosserhoff AK (2013) Increased KGF expression promotes fibroblast activation in a double paracrine manner resulting in cutaneous fibrosis. J Invest Dermatol 133:647–657

35. Carroll LA, Hanasono MM, Mikulec AA, et al (2002) Triamcinolone stimulates bFGF production and inhibits TGF-β1 production by human dermal fibroblasts. Dermatologic Surg 28:704–709

36. Carroll LA, Koch RJ (2003) Heparin stimulates production of bFGF and TGF-β1 by human normal, keloid, and fetal dermal fibroblasts. Med Sci Monit 9:BR97-108

37. Chen D, Wang Q, Bao W, et al (2003) Role of HLA-DR and CD1a molecules in pathogenesis of hypertrophic scarring and keloids. Chin Med J (Engl) 116:314–315

38. Chen ZD, Zhou L, Won T, et al (2018) Characterization of CD45RO+ memory T lymphocytes in keloid disease*. Br J Dermatol 178:940–950

39. Chin GS, Liu W, Peled Z, et al (2001) Differential expression of transforming growth factor-β receptors I and II and activation of Smad 3 in keloid fibroblasts. Plast. Reconstr. Surg. 108:423–429

40. Chipev CC, Simman R, Hatch G, et al (2000) Myofibroblast phenotype and apoptosis in keloid and palmar fibroblasts in vitro. Cell Death Differ 7:166–76

41. Chipev CC, Simon M (2002) Phenotypic differences between dermal fibroblasts from different body sites determine their response to tension and TGFβ1. BMC Dermatol 2:13

42. Chua AWC, Ma D, Gan SU, et al (2011) The role of R-spondin2 in keratinocyte proliferation and epidermal thickening in keloid scarring. J Invest Dermatol 131:644–654

43. Clore JN, Cohen IK, Diegelmann RF (1979) Quantitative assay of types I and III collagen synthesized by keloid biopsies and fibroblasts. Biochim Biophys Acta 586:384–390

44. Concannon MJ, Barrett BB, Adelstein EH, et al (1993) The inhibition of fibroblast proliferation by a novel monokine: an in vitro and in vivo study. J Burn Care Rehabil 14:141–147

45. Conway H, Gillette R, Smith JW, Findley A (1960) Differential diagnosis of keloids and hypertrophic scars by tissue culture technique with notes on therapy of keloids by surgical excision and decadron. Plast Reconstr Surg 25:117–132

46. Daian T, Ohtsuru A, Rogounovitch T, et al (2003) Insulin-like growth factor-I enhances transforming growth factor-β-induced extracellular matrix protein production through the P38/activating transcription factor-2 signaling pathway in keloid fibroblasts. J Invest Dermatol 132:956–962

47. Dalkowski A, Fimmel S, Beutler C, Zouboulis CC (2003) Cryotherapy modifies synthetic activity and differentiation of keloidal fibroblasts in vitro. Exp Dermatol 12:673–681

48. Dalkowski A, Schuppan D, Orfanos CE, Zouboulis CC (1999) Increased expression of tenascin C by keloids in vivo and in vitro. Br J Dermatol 141:50–56

49. Dienus K, Bayat A, Gilmore BF, Seifert O (2010) Increased expression of fibroblast activation protein-alpha in keloid fibroblasts: implications for development of a novel treatment option. Arch Dermatol Res 302:725–731

50. Do D V., Ong CT, Khoo YT, et al (2012) Interleukin-18 system plays an important role in keloid pathogenesis via epithelial-mesenchymal interactions. Br J Dermatol 166:1275–1288

51. Dohi T, Miyake K, Aoki M, et al (2015) Tissue inhibitor of metalloproteinase-2 suppresses collagen synthesis in cultured keloid fibroblasts. Plast Reconstr Surg - Glob Open 3:e520

52. Drummond PD, Dawson LF, Wood FM, Fear MW (2017) Up-regulation of α1-adrenoceptors in burn and keloid scars. Burns 44:582–588

53. E Y, Qipa Z, Hengshu Z (2014) The expression of DNMT1 in pathologic scar fibroblasts and the effect of 5-aza-2-deoxycytidine on cytokines of pathologic scar fibroblasts. Wounds 26:139–46

54. Ehrlich HP, Desmoulière A, Diegelmann RF, et al (1994) Morphological and immunochemical differences between keloid and hypertrophic scar. Am J Pathol 145:105–113

55. Fang F, Huang R, Zheng Y, et al (2016) Bone marrow derived mesenchymal stem cells inhibit the proliferative and pro fi brotic phenotype of hypertrophic scar fibroblasts and keloid fibroblasts through paracrine signaling. J Dermatol Sci 83:95–105

56. De Felice B, Wilson RR, Nacca M (2009) Telomere shortening may be associated with human keloids. BMC Med Genet 10:110

57. Friedman DW, Boyd CD, Mackenzie JW, et al (1993) Regulation of collagen gene expression in keloids and hypertrophic scars. J. Surg. Res. 55:214–222

58. Fujiwara M, Muragaki Y, Ooshima A (2005) Keloid-derived fibroblasts show increased secretion of factors involved in collagen turnover and depend on matrix metalloproteinase for migration. Br J Dermatol 153:295–300

59. Fujiwara M, Muragaki Y, Ooshima A (2005) Upregulation of transforming growth factor-β1 and vascular endothelial growth factor in cultured keloid fibroblasts: relevance to angiogenic activity. Arch Dermatol Res 297:161–169

60. Funayama E, Chodon T, Oyama A, Sugihara T (2003) Keratinocytes promote proliferation and inhibit apoptosis of the underlying fibroblasts: an important role in the pathogenesis of keloid. J Invest Dermatol 121:1326–1331

61. Gadson PF, Russell JD, Russel SB (1984) Glucocorticoid receptors in human fibroblasts derived from normal dermis and keloid tissue. J Biol Chem 259:11236–11241

62. Gao Z, Wu X, Song N, et al (2010) Differential expression of growth differentiation factor-9 in keloids. Burns 36:1289–1295

63. Ghazizadeh M, Tosa M, Shimizu H, et al (2007) Functional implications of the IL-6 signaling pathway in keloid pathogenesis. J Invest Dermatol 127:98–105

64. Gira AK, Brown LF, Washington C V., et al (2004) Keloids demonstrate high-level epidermal expression of vascular endothelial growth factor. J Am Acad Dermatol 50:850–853

65. Giugliano G, Pasquali D, Notaro A, et al (2003) Verapamil inhibits interleukin-6 and vascular endothelial growth factor production in primary cultures of keloid fibroblasts. Br J Plast Surg 56:804–809

66. Hahn JM, Glaser K, McFarland KL, et al (2013) Keloid-derived keratinocytes exhibit an abnormal gene expression profile consistent with a distinct causal role in keloid pathology. Wound Repair Regen 21:530–544

67. Hahn JM, Mcfarland KL, Combs KA, Supp DM (2016) Partial epithelial-mesenchymal transition in keloid scars: regulation of keloid keratinocyte gene expression by transforming growth factor-β1. Burn Trauma 4:1–17

68. Hahn JM, Supp DM (2017) Abnormal expression of the vitamin D receptor in keloid scars. Burns 43:1506–1515

69. Haisa M, Okochi H, Grotendorst GR (1994) Elevated levels of PDGF-α receptors in keloid fibroblasts contribute to an enhanced response to PDGF. J Invest Dermatol 103:560–563

70. Hanasono MM, Lum J, Carroll LA, et al (2004) The effect of silicone gel on basic fibroblast growth factor levels in fibroblast cell culture. Arch Facial Plast Surg 6:88–93

71. Hanasono MM, Kita M, Mikulec AA, et al (2003) Autocrine growth factor production by fetal, keloid, and normal dermal fibroblasts. Arch Facial Plast Surg 5:26–30

72. Hasegawa T, Nakao A, Sumiyoshi K, et al (2003) IFN-γ fails to antagonize fibrotic effect of TGF-β on keloid-derived dermal fibroblasts. J Dermatol Sci 32:19–24

73. Hasegawa T, Nakao A, Sumiyoshi K, et al (2005) SB-431542 inhibits TGF-β-induced contraction of collagen gel by normal and keloid fibroblasts. J Dermatol Sci 39:33–38

74. Hayashi T, Nishihira J, Koyama Y, et al (2006) Decreased prostaglandin E2 production by inflammatory cytokine and lower expression of EP2 receptor result in increased collagen synthesis in keloid fibroblasts. J Invest Dermatol 126:990–997

75. He S, Yang Y, Liu X, et al (2012) Compound Astragalus and Salvia Miltiorrhiza extract inhibits cell proliferation, invasion and collagen synthesis in keloid fibroblasts by mediating transforming growth factor-β/Smad pathway. Br J Dermatol 166:564–574

76. Heitzer E, Seidl H, Bambach I, et al (2012) Infrequent p53 gene mutation but UV gradient-like p53 protein positivity in keloids. Exp Dermatol 21:277–280

77. Hellström M, Hellström S, Engström-Laurent A, Bertheim U (2014) The structure of the basement membrane zone differs between keloids, hypertrophic scars and normal skin: a possible background to an impaired function. J Plast Reconstr Aesthetic Surg 67:1564–1572

78. Higgins PJ, Slack JK, Diegelmann RF, Staiano-Coico L (1999) Differential regulation of PAI-1 gene expression in human fibroblasts predisposed to a fibrotic phenotype. Exp Cell Res 248:634–642

79. Hochman B, Nahas FX, Sobral CS, et al (2008) Nerve fibres: a possible role in keloid pathogenesis. Br J Dermatol 158:624–657

80. Hsu C, Lin H, Harn HI, et al (2018) Caveolin-1 controls hyperresponsiveness to mechanical stimuli and activation in keloid fibroblasts. J Invest Dermatol 138:208–218

81. Hsu YC, Hsiao M, Wang LF, et al (2006) Nitric oxide produced by iNOS is associated with collagen synthesis in keloid scar formation. Nitric Oxide - Biol Chem 14:327–334

82. Hu Z-C, Tang B, Guo D, et al (2014) Expression of insulin-like growth factor-1 receptor in keloid and hypertrophic scar. Clin Exp Dermatol 39:822–828

83. Huang C, Akaishi S, Hyakusoku H, Ogawa R (2014) Are keloid and hypertrophic scar different forms of the same disorder? A fibroproliferative skin disorder hypothesis based on keloid findings. Int Wound J 11:517–522

84. Igota S, Tosa M, Murakami M, et al (2013) Identification and characterization of Wnt signaling pathway in keloid pathogenesis. Int J Med Sci 10:344–354

85. Ikeda M, Naitoh M, Kubota H, et al (2009) Elastic fiber assembly is disrupted by excessive accumulation of chondroitin sulfate in the human dermal fibrotic disease, keloid. Biochem Biophys Res Commun 390:1221–1228

86. Imaizumi R, Akasaka Y, Inomata N, et al (2009) Promoted activation of matrix metalloproteinase (MMP)-2 in keloid fibroblasts and increased expression of MMP-2 in collagen bundle regions: Implications for mechanisms of keloid progression. Histopathology 54:722–730

87. Inui S, Shono F, Nakajima T, et al (2011) Identification and characterization of cartilage oligomeric matrix protein as a novel pathogenic factor in keloids. Am J Pathol 179:1951–1960

88. Iqbal SA, Sidgwick GP, Bayat A (2012) Identification of fibrocytes from mesenchymal stem cells in keloid tissue: A potential source of abnormal fibroblasts in keloid scarring. Arch Dermatol Res 304:665–671

89. Iqbal SA, Syed F, McGrouther DA, et al (2010) Differential distribution of haematopoietic and nonhaematopoietic progenitor cells in intralesional and extralesional keloid: do keloid scars provide a niche for nonhaematopoietic mesenchymal stem cells? Br J Dermatol 162:1377–1383

90. Janssen de Limpens AMP, Cormane RH (1982) Studies on the immunologic aspects of keloids and hypertrophic scars. Arch Dermatol Res 274:259–266

91. Javad F, Marriage F, Bayat A (2012) Perturbation of cell cycle expression in keloid fibroblast. Skinmed 10:152–159

92. Jiao H, Fan J, Cai J, et al (2015) Analysis of characteristics similar to autoimmune disease in keloid patients. Aesthetic Plast Surg 39:818–825

93. Jiao H, Zhang T, Fan J, Xiao R (2017) The superficial dermis may initiate keloid formation: histological analysis of the keloid dermis at different depths. Front Physiol 8:1–9

94. Jin Q, Gui L, Niu F, et al (2018) Macrophages in keloid are potent at promoting the differentiation and function of regulatory T-cells. Exp Cell Res 362:472–476

95. Jin Z (2014) Increased c-Met phosphorylation is related to keloid pathogenesis: implications for the biological behaviour of keloid fibroblasts. Pathology 46:25–31

96. Jing C, Jia-han W, Hong-Xing Z (2010) Double-edged effects of neuropeptide substance P on repair of cutaneous trauma. Wound Repair Regen 18:319–324

97. Jumper N, Paus R, Bayat A (2015) Functional histopathology of keloid disease. Histol Histopathol 30:1033–1057

98. Jumper N, Hodgkinson T, Arscott G, et al (2016) The aldo-keto reductase AKR1B10 is up-regulated in keloid epidermis, implicating retinoic acid pathway dysregulation in the pathogenesis of keloid disease. J Invest Dermatol 136:1500–1512

99. Jumper N, Hodgkinson T, Paus R, Bayat A (2017) A role for Neuregulin-1 in promoting keloid fibroblast migration. Acta Derm Venereol 97:675–684

100. Jurzak M, Adamczyk K (2013) Influence of genistein on c-jun, c-fos and fos-b of ap-1 subunits expression in skin keratinocytes, fibroblasts and keloid fibroblasts cultured in vitro. Acta Pol Pharm - Drug Res 70:205–213

101. Jurzak M, Adamczyk K, Antończak P, et al (2014) Evaluation of genistein ability to modulate CTGF mRNA/protein expression, genes expression of TGF-β isoforms and expression of selected genes regulating cell cycle in keloid fibroblasts in vitro. Acta Pol Pharm - Drug Res 71:972–986

102. Kamamoto F, Oliveira Paggiaro A, Rodas A, et al (2003) A wound contraction experimental model for studying keloids and wound-healing modulators. Artif Organs 27:701–705

103. Kamath NV, Ormsby A, Bergfeld WF, House NS (2002) A light microscopic and immunohistochemical evaluation of scars. J Cutan Pathol 29:27–32

104. Khoo YT, Ong CT, Mukhopadhyay A, et al (2006) Upregulation of secretory connective tissue growth factor (CTGF) in keratinocyte-fibroblast coculture contributes to keloid pathogenesis. J Cell Physiol 208:336–343

105. Kim J, Kim B, Kim SM, et al (2019) Hypoxia-induced epithelial-to-mesenchymal transition mediates fibroblast abnormalities via EKR activation in cutaneous wound healing. Int J Mol Sci 20:1–14

106. Kim JE, Lee JH, Jeong KH, et al (2014) Notch intracellular domain expression in various skin fibroproliferative diseases. Ann Dermatol 26:332–337

107. Kischer CW, Hendrix MJC (1983) Fibronectin (FN) in hypertrophic scars and keloids. Cell Tissue Res 231:29–37

108. Kischer CW, Pindur J (1990) Effects of platelet derived growth factor (PDGF) on fibronectin (FN) production by human skin and scar fibroblasts. Cytotechnology 3:231–238

109. Kiya K, Kubo T, Kawai K, et al (2016) Endothelial cell-­derived endothelin-1 is involved in abnormal scar formation by dermal fibroblasts through RhoA/Rho-kinase pathway. Exp Dermatol 26:705–712

110. Kurokawa N, Ueda K, Tsuji M (2010) Study of microvascular structure in keloid and hypertrophic scars: density of microvessels and the efficacy of three-dimensional vascular imaging. J Plast Surg Hand Surg 44:272–277

111. Kuwahara H, Tosa M, Murakami M, et al (2016) Examination of epithelial mesenchymal transition in keloid tissues and possibility of keloid therapy target. Plast Reconstr Surg Glob Open 4:1–7

112. Ladin DA, Hou Z, Patel D, et al (1998) P53 and apoptosis alterations in keloids and keloid fibroblasts. Wound Repair Regen 6:28–37

113. Laurentaci G, Dioguardi D (1977) HLA antigens in keloids and hypertrophic scars. Arch Dermatol 113:1726

114. Leake D, Doerr TD, Scott G (2003) Expression of urokinase-type plasminogen activator and its receptor in keloids. Arch Otolaryngol – Head Neck Surg 129:1334–1338

115. Lee CH, Hong CH, Chen YT, et al (2012) TGF-beta1 increases cell rigidity by enhancing expression of smooth muscle actin: keloid-derived fibroblasts as a model for cellular mechanics. J Dermatol Sci 67:173–180

116. Lee JH, Shin JU, Jung I, et al (2013) Proteomic profiling reveals upregulated protein expression of Hsp70 in keloids. Biomed Res Int 2013:621538

117. Lee JYY, Yang CC, Chao SC, Wong TW (2004) Histopathological differential diagnosis of keloid and hypertrophic scar. Am J Dermatopathol 26:379–384

118. Lee KS, Song JY, Suh MH (1991) Collagen mRNA expression detected by in situ hybridization in keloid tissue. J Dermatol Sci 2:316–323

119. Lee WJ, Kim YO, Choi IK, et al (2011) Adenovirus-relaxin gene therapy for keloids: implication for reversing pathological fibrosis. Br J Dermatol 165:673–677

120. Lee WJ, Park JH, Shin JU, et al (2015) Endothelial-to-mesenchymal transition induced by Wnt3a in keloid pathogenesis. Wound Repair Regen 23:435–442

121. Lee YJ, Kwon SB, Kim CH, et al (2015) Oxidative damage and nuclear factor erythroid 2-related factor 2 protein expression in normal skin and keloid tissue. Ann Dermatol 27:507–516

122. Lee YS, Liang YC, Wu P, et al (2019) STAT3 signalling pathway is implicated in keloid pathogenesis by preliminary transcriptome and open chromatin analyses. Exp Dermatol 28:480–484

123. Li H, Nahas Z, Feng F, et al (2013) Tissue engineering for in vitro analysis of matrix metalloproteinases in the pathogenesis of keloid lesions. JAMA Facial Plast Surg 15:448–456

124. Li M, Wu L (2016) Functional analysis of keratinocyte and fibroblast gene expression in skin and keloid scar tissue based on deviation analysis of dynamic capabilities. Exp Ther Med 12:3633–3641

125. Liang C, Yen Y, Hung L, et al (2013) Thalidomide inhibits fibronectin production in TGF-β1-treated normal and keloid fibroblasts via inhibition of the p38/SMAD3 pathway. Biochem Pharmacol 85:1594–1602

126. Liao WT, Yu HS, Arbiser JL, et al (2010) Enhanced MCP-1 release by keloid CD14+ cells augments fibroblast proliferation: role of MCP-1 and Akt pathway in keloids. Exp Dermatol 19:e142–e150

127. Lim CP, Phan TT, Lim IJ, Cao X (2006) Stat3 contributes to keloid pathogenesis via promoting collagen production, cell proliferation and migration. Oncogene 25:5416–5425

128. Lim CP, Phan TT, Lim IJ, Cao X (2009) Cytokine profiling and Stat3 phosphorylation in epithelial-mesenchymal interactions between keloid keratinocytes and fibroblasts. J Invest Dermatol 129:851–861

129. Lim CK, Halim AS, Yaacob NS, et al (2013) Keloid pathogenesis via Drosophila similar to mothers against decapentaplegic (SMAD) signaling in a primary epithelial-mesenchymal in vitro model treated with biomedical-grade chitosan porous skin regenerating template. J Biosci Bioeng 115:453–458

130. Lim D, Phan TT, Yip GW, Bay BH (2006) Up-regulation of metallothionein isoforms in keloid keratinocytes. Int J Mol Med 17:385–389

131. Lim IJ, Phan TT, Tan EK, et al (2003) Synchronous activation of ERK and phosphatidylinositol 3-kinase pathways is required for collagen and extracellular matrix production in keloids. J Biol Chem 278:40851–40858

132. Lim IJ, Phan T-T, Bay B-H, et al (2002) Fibroblasts cocultured with keloid keratinocytes: normal fibroblasts secrete collagen in a keloidlike manner. Am J Physiol Cell Physiol 283:C212–C222

133. Limandjaja GC, Belien JM, Scheper RJ, et al (2019) Hypertrophic and keloid scars fail to progress from the CD34-/α-smooth muscle actin (α-SMA)+ immature scar phenotype and show gradient differences in α-SMA and p16 expression. Br J Dermatol Jun 17:[Epub ahead of print]

134. Limandjaja GC, van den Broek LJ, Waaijman T, et al (2017) Increased epidermal thickness and abnormal epidermal differentiation in keloid scars. Br J Dermatol 176:116–126

135. Limandjaja GC, Waaijman T, Roffel S, et al (2019) Monocytes co-cultured with reconstructed keloid and normal skin models skew towards M2 macrophage phenotype. Arch Dermatol Res 311:615–627

136. Lin L, Wang Y, Liu W, Huang Y (2015) BAMBI inhibits skin fibrosis in keloid through suppressing TGF-β1-induced hypernomic fibroblast cell proliferation and excessive accumulation of collagen I. Int J Clin Exp Med 8:13227–13234

137. Lu F, Gao J, Ogawa R, et al (2007) Fas-mediated apoptotic signal transduction in keloid and hypertrophic scar. Plast Reconstr Surg 119:1714–1721

138. Luo LF, Shi Y, Zhou Q, et al (2013) Insufficient expression of the melanocortin-1 receptor by human dermal fibroblasts contributes to excess collagen synthesis in keloid scars. Exp Dermatol 22:764–766

139. Luo L, Li J, Liu H, et al (2017) Adiponectin is involved in connective tissue growth factor-induced proliferation, migration and overproduction of the extracellular matrix in keloid fibroblasts. Int J Mol Sci 18:1–21

140. Luo S, Benathan M, Raffoul W, et al (2001) Abnormal balance between proliferation and apoptotic cell death in fibroblasts derived from keloid lesions. Plast. Reconstr. Surg. 107:87–96

141. Ma X, Chen J, Xu B, et al (2015) Keloid-derived keratinocytes acquire a fibroblast-like appearance and an enhanced invasive capacity in a hypoxic microenvironment in vitro. Int J Mol Med 35:1246–1256

142. Maeda D, Kubo T, Kiya K, et al (2019) Periostin is induced by IL-4/IL-13 in dermal fibroblasts and promotes RhoA/ROCK pathway-mediated TGF-β1 secretion in abnormal scar formation. J Plast Surg Hand Surg May 8:1–7

143. Makino S, Mitsutake N, Nakashima M (2008) DHMEQ, a novel NF-kappaB inhibitor, suppresses growth and type I collagen accumulation in keloid fibroblasts. J Dermatol Sci 51:171—180

144. Mantel A, Newsome A, Thekkudan T, et al (2016) The role of aldo-keto reductase 1C3 (AKR1C3)-mediated prostaglandin D2 (PGD2) metabolism in keloids. Exp Dermatol 25:38–43

145. Materazzi S, Pellerito S, Di Serio C, et al (2007) Analysis of protease-activated receptor-1 and -2 in human scar formation. J Pathol 212:440–449

146. Mathangi Ramakrishnan K, Meenakshi Janakiraman M, Babu M (2012) Expression of fibrocyte markers by keloid fibroblasts: an insight into fibrosis during burn wound healing - a preliminary study. Ann Burns Fire Disasters 25:148–151

147. McCauley RL, Vimlarani C, Ying-Yue L, et al (1992) Altered cytokines production in black patients with keloids. J Clin Immunol 12:300–308

148. Mccormack MC, Nowak KC, Koch RJ (2001) The effect of copper tripeptide and tretinoin on growth factor production in a serum-free fibroblast model. Arch Facial Plast Surg 3:28–32

149. McCoy BJ, Galdun J, Cohen K (1982) Effects of density and cellular aging on collagen synthesis and growth kinetics in keloid and normal skin fibroblasts. In Vitro 18:79–86

150. McFarland KL, Glaser K, Hahn JM, et al (2011) Culture medium and cell density impact gene expression in normal skin and abnormal scar-derived fibroblasts. J Burn Care Res 32:498–508

151. Meenakshi J, Jayaraman V, Ramakrishnan KM, Babu M (2005) Ultrastructural differentiation of abnormal scars. Ann Burn Fire Disasters 18:83–88

152. Meenakshi J, Vidyameenakshi S, Ananthram D, et al (2009) Low decorin expression along with inherent activation of ERK1,2 in earlobe keloids. Burns 35:519–526

153. Messadi DV, Le A, Berg S, et al (1998) Effect of TGF-β1 on PDGF receptor expression in human scar fibroblasts. Front Biosci 3:a16–a22

154. Messadi D V., Doung HS, Zhang Q, et al (2004) Activation of NFκB signal pathways in keloid fibroblasts. Arch Dermatol Res 296:125–133

155. Messadi D V., Le A, Berg S, et al (1999) Expression of apoptosis-associated genes by human dermal scar fibroblasts. Wound Repair Regen 7:511–517

156. Meyer LJM, Russell SB, Russell JD, et al (2000) Reduced hyaluronan in keloid tissue and cultured keloid fibroblasts. J Invest Dermatol 114:953–959

157. Mikulec AA, Hanasono MM, Lum J, et al (2001) Effect of tamoxifen on transforming growth factor β1 production by keloid and fetal fibroblasts. Arch Facial Plast Surg 3:111–114

158. Mills BG, Frausto A, Brien E (2000) Cytokines associated with the pathophysiology of aggressive fibromatosis. J Orthop Res 18:655–662

159. Moon J-H, Kwak SS, Park G, et al (2008) Isolation and characterization of multipotent human keloid-derived mesenchymal-like stem cells. Stem Cells Dev 17:713–724

160. Moshref S, Mufti ST (2009) Keloid and hypertrophic scars: comparative histopathological and immunohistochemical study. J King Abdulaziz Univ - Med Sci 17:3–22

161. Mukhopadhyay A, Do D V., Ong CT, et al (2011) The role of stem cell factor and c-KIT in keloid pathogenesis: do tyrosine kinase inhibitors have a potential therapeutic role? Br J Dermatol 164:372–386

162. Mukhopadhyay A, Tan EKJ, Khoo YTA, et al (2005) Conditioned medium from keloid keratinocyte/keloid fibroblast coculture induces contraction of fibroblast-populated collagen lattices. Br J Dermatol 152:639–645

163. Mukhopadhyay A, Khoo A, Cheong HH, et al (2007) Targeting of Sp1 transcription factor: a novel therapeutic approach for keloids, an in vitro analysis. Exp Dermatol 16:1023–1031

164. Mukhopadhyay A, Chan SY, Lim IJ, et al (2007) The role of the activin system in keloid pathogenesis. Am J Physiol Cell Physiol 292:C1331–C1338

165. Mukhopadhyay A, Fan S, Dang VD, et al (2010) The role of hepatocyte growth factor/c-Met system in keloid pathogenesis. J Trauma - Inj Infect Crit Care 69:1457–1466

166. Mukhopadhyay A, Wong MY, Chan SY, et al (2010) Syndecan-2 and decorin: proteoglycans with a difference-implications in keloid pathogenesis. J Trauma - Inj Infect Crit Care 68:999–1008

167. Murao N, Seino K ichiro, Hayashi T, et al (2014) Treg-enriched CD4+ T cells attenuate collagen synthesis in keloid fibroblasts. Exp Dermatol 23:266–271

168. Muthusubramaniam L, Zaitseva T, Paukshto M, et al (2014) Effect of collagen nanotopography on keloid fibroblast proliferation and matrix synthesis: implications for dermal wound healing. Tissue Eng Part A 20:2728–2736

169. Naitoh M, Hosokawa N, Kubota H, et al (2001) Upregulation of HSP47 and collagen type III in the dermal fibrotic disease, keloid. Biochem Biophys Res Commun 280:1316–1322

170. Naylor MC, Lazar DA, Zamora IJ, et al (2012) Increased in vitro differentiation of fibrocytes from keloid patients is inhibited by serum amyloid P. Wound Repair Regen 20:277–283

171. Niazi F, Hooshyar SH, Hedayatyanfard K, et al (2018) Detection of angiotensin II and AT1 receptor concentrations in keloid and hypertrophic scar. J Clin Aesthet Dermatol 11:36–39

172. Nirodi CS, Devalaraja R, Nanney LB, et al (2000) Chemokine and chemokine receptor expression in keloid and normal fibroblasts. Wound Repair Regen 8:371–382

173. Nowak KC, McCormack M, Koch RJ (2000) The effect of superpulsed carbon dioxide laser energy on keloid and normal dermal fibroblast secretion of growth factors: a serum-free study. Plast Reconstr Surg 105:2039–2048

174. Ohtsuru A, Yoshimoto H, Ishihara H, et al (2000) Insulin-like growth factor-I (IGF-I)/IGF-I receptor axis and increased invasion activity of fibroblasts in keloid. Endocr J 47 Suppl:S41–S44

175. Oku T, Takigawa M, Yamada M (1987) Cell proliferation kinetics of cultured human keratinocytes and fibroblasts measured using a monoclonal antibody. Br J Dermatol 116:673–679

176. Ong CT, Khoo YT, Mukhopadhyay A, et al (2007) mTOR as a potential therapeutic target for treatment of keloids and excessive scars. Exp Dermatol 16:394–404

177. Ong CT, Khoo YT, Mukhopadhyay A, et al (2010) Comparative proteomic analysis between normal skin and keloid scar. Br J Dermatol 162:1302–1315

178. Ong CT, Khoo YT, Tan EK, et al (2007) Epithelial–mesenchymal interactions in keloid pathogenesis modulate vascular endothelial growth factor expression and secretion. J Pathol 211:95–108

179. Onodera M, Ueno M, Ito O, et al (2007) Factor XIIIa-positive dermal dendritic cells in keloids and hypertrophic and mature scars. Pathol Int 57:337–342

180. Ooi BNS, Mukhopadhyay A, Masilamani J, et al (2010) Hepatoma-derived growth factor and its role in keloid pathogenesis. J Cell Mol Med 14:1328–1337

181. Pavelecini M, Zettler CG, Fernandes MC, Ely PB (2019) Experimental immunohistochemical expression of cyclooxygenases in hypertrophic scars and keloids. Plast Reconstr Surg Glob Open 7:1–7

182. Phan TT, Lim IJ, Bay BH, et al (2003) Role of IGF system of mitogens in the induction of fibroblast proliferation by keloid-derived keratinocytes in vitro. Am J Physiol Cell Physiol 284:C860–C869

183. Phan TT, Lim IJ, Bay BH, et al (2002) Differences in collagen production between normal and keloid-derived fibroblasts in serum-media co-culture with keloid-derived keratinocytes. J Dermatol Sci 29:26–34

184. Plikus MV, Guerrero-Juarez CF, Ito M, et al (2017) Regeneration of fat cells from myofibroblasts during wound healing. Science 355:748–752

185. Romero-Valdovinos M, Cárdenas-Mejía A, Gutiérrez-Gómez C, et al (2011) Keloid skin scars: the influence of hyperbaric oxygenation on fibroblast growth and on the expression of messenger RNA for insulin like growth factor and for transforming growth factor. Vitr Cell Dev Biol - Anim 47:421–424

186. Rossiello L, D’Andrea F, Grella R, et al (2009) Differential expression of cyclooxygenases in hypertrophic scar and keloid tissues. Wound Repair Regen 17:750–757

187. Rünger TM, Quintanilla-Dieck MJ, Bhawan J (2007) Role of cathepsin K in the turnover of the dermal extracellular matrix during scar formation. J Invest Dermatol 127:293–297

188. Russell JD, Witt WS (1976) Cell size and growth characteristics of cultured fibroblasts isolated from normal and keloid tissue. Plast Reconstr Surg 57:207–212

189. Russell SB, Trupin JS, Myers JC, et al (1989) Differential glucocorticoid regulation of collagen mRNAs in human dermal fibroblasts. Keloid-derived and fetal fibroblasts are refractory to down-regulation. J Biol Chem 264:13730–13735

190. Russell SB, Russell JD, Trupin KM, et al (2010) Epigenetically altered wound healing in keloid fibroblasts. J Invest Dermatol 130:2489–2496

191. Russell SB, Trupin JS, Kennedy RZ, et al (1995) Glucocorticoid regulation of elastin synthesis in human fibroblasts: down-regulation in fibroblasts from normal dermis but not from keloids. J Invest Dermatol 104:241–245

192. Russell SB, Trupin KM, Rodríguez-Eaton S, et al (1988) Reduced growth-factor requirement of keloid-derived fibroblasts may account for tumor growth. Proc Natl Acad Sci U S A 85:587–91

193. Saed GM, Ladin D, Olson J, et al (1998) Analysis of p53 gene mutations in keloids using polymerase chain reaction-based single-strand conformational polymorphism and DNA sequencing. Arch Dermatol 134:963–967

194. Saffari TM, Bijlard E, van Bodegraven EAM, et al (2018) Sensory perception and nerve fibre innervation in patients with keloid scars: an investigative study. Eur J Dermatology 28:828–829

195. Sahara K, Kucukcelebi A, Ko F, et al (1993) Suppression of in vitro proliferative scar fibroblast contraction by interferon alfa‐2b. Wound Repair Regen 1:22–27

196. Saito M, Yamazaki M (2012) Pirfenidone suppresses keloid fibroblast-embedded collagen gel contraction. Arch Dermatol Res 304:217–222

197. Santucci M, Borgognoni L, Reali UM, Gabbiani G (2001) Keloids and hypertrophic scars of Caucasians show distinctive morphologic and immunophenotypic profiles. Virchows Arch 438:457–463

198. Satish L, Lyons-Weiler J, Hebda PA, Wells A (2006) Gene expression patterns in isolated keloid fibroblasts. Wound Repair Regen 14:463–470

199. Sato H, Suzuki A, Funahashi M, et al (1996) Characteristics of growth, morphology, contractility, and protein expression of fibroblasts derived from keloid. Wound Repair Regen 4:103–114

200. Sato M, Ishikawa O, Miyachi Y (1998) Distinct patterns of collagen gene expression are seen in normal and keloid fibroblasts grown in three-dimensional culture. Br J Dermatol 138:938–943

201. Sato M (2006) Upregulation of the Wnt/β-catenin pathway induced by transforming growth factor-β in hypertrophic scars and keloids. Acta Derm Venereol 86:300–307

202. Seifert O, Bayat A, Geffers R, et al (2008) Identification of unique gene expression patterns within different lesional sites of keloids. Wound Repair Regen 16:254–265

203. Seleit I, Bakry OA, Samaka RM, Tawfik AS (2016) Immunohistochemical evaluation of leptin expression in wound healing: a clue to exuberant scar formation. Appl Immunohistochem Mol Morphol 24:296–306

204. Shaker SA, Ayuob NN, Hajrah NH (2011) Cell talk: a phenomenon observed in the keloid scar by immunohistochemical study. Appl Immunohistochem Mol Morphol 19:153–159

205. Shang T, Yao B, Gao D, et al (2018) A novel model of humanised keloid scarring in mice. Int Wound J 15:90–94

206. Sharquie KE, Al-Dhalimi MA (2003) Keloid in Iraqi patients: a clinicohistopathologic study. Dermatologic Surg 29:847–851

207. Shih B, McGrouther DA, Bayat A (2010) Identification of novel keloid biomarkers through profiling of tissue biopsies versus cell cultures in keloid margin specimens compared to adjacent normal skin. Eplasty 10:e24

208. Shin JU, Kim SH, Kim H, et al (2016) TSLP is a potential initiator of collagen synthesis and an activator of CXCR4/SDF-1 axis in keloid pathogenesis. J Invest Dermatol 136:507–515

209. Sible JC, Eriksson E, Smith SP, Oliver N (1994) Fibronectin gene expression differs in normal and abnormal human wound healing. Wound Repair Regen 2:3–19

210. Sidgwick GP, Iqbal SA, Bayat A (2013) Altered expression of hyaluronan synthase and hyaluronidase mRNA may affect hyaluronic acid distribution in keloid disease compared with normal skin. Exp Dermatol 22:377–379

211. Slominski A, Wortsman J, Mazurkiewicz JE, et al (1993) Detection of proopiomelanocortin-derived antigens in normal and pathologic human skin. J Lab Clin Med 122:658–66

212. Slot AJ Van Der, Zuurmond A, Bogaerdt AJ Van Den, et al (2004) Increased formation of pyridinoline cross-links due to higher telopeptide lysyl hydroxylase levels is a general fibrotic phenomenon. Matrix Biol 23:251–257

213. Smith JC, Boone BE, Opalenik SR, et al (2008) Gene profiling of keloid fibroblasts shows altered expression in multiple fibrosis-associated pathways. J Invest Dermatol 128:1298–1310

214. Smoller BR, McNutt NS, Hsu A (1989) HMB-45 recognizes stimulated melanocytes. J Cutan Pathol 16:49–53

215. Sogabe Y, Akimoto S, Abe M, et al (2002) Functions of the stratum corneum in systemic sclerosis as distinct from hypertrophic scar and keloid functions. J Dermatol Sci 29:49–53

216. Spiekman M, Przybyt E, Plantinga JA, et al (2014) Adipose tissue-derived stromal cells inhibit TGF-β1-induced differentiation of human dermal fibroblasts and keloid scar-derived fibroblasts in a paracrine fashion. Plast Reconstr Surg 134:699–712

217. Suarez E, Syed F, Alonso-Rasgado T, et al (2013) Up-regulation of tension-related proteins in keloids: knockdown of HSP27, α2β1-integrin, and PAI-2 shows convincing reduction of extracellular matrix production. Plast Reconstr Surg 131:158–173

218. Suarez E, Syed F, Alonso-Rasgado T, Bayat A (2015) Identification of biomarkers involved in differential profiling of hypertrophic and keloid scars versus normal skin. Arch Dermatol Res 307:115–133

219. Suetake T, Sasai S, Zhen YX, et al (1996) Functional analyses of the stratum corneum in scars. Sequential studies after injury and comparison among keloids, hypertrophic scars, and atrophic scars. Arch Dermatol 132:1453–1458

220. Sumi Y, Muramatsu H, Hata KI, et al (2000) Secretory leukocyte protease inhibitor is a novel inhibitor of fibroblast-mediated collagen gel contraction. Exp Cell Res 256:203–212

221. Supp DM, Hahn JM, McFarland KL, Glaser K (2014) Inhibition of hyaluronan synthase 2 reduces the abnormal migration rate of keloid keratinocytes. J Burn Care Res 35:84–92

222. Suttho D, Mankhetkorn S, Binda D, et al (2017) 3D modeling of keloid scars in vitro by cell and tissue engineering. Arch Dermatol Res 309:55–62

223. Suzawa H, Kikuchi S, Arai N, Koda A (1992) The mechanism involved in the inhibitory action of tranilast on collagen biosynthesis of keloid fibroblasts. Jpn J Pharmacol 60:91–96

224. Syed F, Ahmadi E, Iqbal SA, et al (2011) Fibroblasts from the growing margin of keloid scars produce higher levels of collagen I and III compared with intralesional and extralesional sites: clinical implications for lesional site-directed therapy. Br J Dermatol 164:83–96

225. Syed F, Bayat A (2012) Notch signaling pathway in keloid disease: enhanced fibroblast activity in a Jagged-1 peptide-dependent manner in lesional vs. extralesional fibroblasts. Wound Repair Regen 20:688–706

226. Szulgit G, Rudolph R, Wandel A, et al (2002) Alterations in fibroblast α1β1 integrin collagen receptor expression in keloids and hypertrophic scars. J Invest Dermatol 118:409–415

227. Tan EML, Hoffren J, Rouda S, et al (1993) Decorin, versican, and biglycan gene expression by keloid and normal dermal fibroblasts: differential regulation by basic fibroblast growth factor. Exp. Cell Res. 209:200–207

228. Tanaka A, Hatoko M, Tada H, et al (2004) Expression of p53 family in scars. J Dermatol Sci 34:17–24

229. Teofoli P, Barduagni S, Ribuffo M, et al (1999) Expression of Bcl-2, p53, c-jun and c-fos protooncogenes in keloids and hypertrophic scars. J Dermatol Sci 22:31–37

230. Theoret CL, Acvs D, Olutoye OO, et al (2013) Equine exuberant granulation tissue and human keloids: a comparative histopathologic study. Vet Surg 42:783–789

231. Touchi R, Ueda K, Kurokawa N, Tsuji M (2016) Central regions of keloids are severely ischaemic. J Plast Reconstr Aesthetic Surg 69:e35–e41

232. Tseng S-H, Hsu C-K, Yu-Yun Lee J, et al (2012) Noninvasive evaluation of collagen and hemoglobin contents and scattering property of in vivo keloid scars and normal skin using diffuse reflectance spectroscopy: pilot study. J Biomed Opt 17:0770051

233. Tsujita-Kyutoku M, Uehara N, Matsuoka Y, et al (2005) Comparison of transforming growth factor-beta/Smad signaling between normal dermal fibroblasts and fibroblasts derived from central and peripheral areas of keloid lesions. In Vivo (Brooklyn) 19:959–963

234. Tuan TL, Wu H, Huang EY, et al (2003) Increased plasminogen activator inhibitor-1 in keloid fibroblasts may account for their elevated collagen accumulation in fibrin gel cultures. Am J Pathol 162:1579–1589

235. Tuan TL, Zhu JY, Sun B, et al (1996) Elevated levels of plasminogen activator inhibitor-1 may account for the altered fibrinolysis by keloid fibroblasts. J Invest Dermatol 106:1007–1011

236. Tucci-Viegas VM, Hochman B, Frana JP, Ferreira LM (2010) Keloid explant culture: a model for keloid fibroblasts isolation and cultivation based on the biological differences of its specific regions. Int Wound J 7:339–348

237. Uchida G, Yoshimura K, Kitano Y, et al (2003) Tretinoin reverses upregulation of matrix metalloproteinase-13 in human keloid-derived fibroblasts. Exp Dermatol 12 Suppl 2:35–42

238. Ueda K, Furuya E, Yasuda Y, et al (1999) Keloids have continuous high metabolic activity. Plast. Reconstr. Surg. 104:694–678

239. Ueda K, Yasuda Y, Furuya E, Oba S (2004) Inadequate blood supply persists in keloids. Scand J Plast Reconstr Surg Hand Surg 38:267–271

240. Uitto J, Perejda AJ, Abergel RP, et al (1985) Altered steady-state ratio of type I/III procollagen mRNAs correlates with selectively increased type I procollagen biosynthesis in cultured keloid fibroblasts. Proc Natl Acad Sci U S A 82:5935–9

241. Ulrich D, Ulrich F, Unglaub F, et al (2010) Matrix metalloproteinases and tissue inhibitors of metalloproteinases in patients with different types of scars and keloids. J Plast Reconstr Aesthetic Surg 63:1015–1021

242. Uzawa K, Yeowell HN, Yamamoto K, et al (2003) Lysine hydroxylation of collagen in a fibroblast cell culture system. Biochem Biophys Res Commun 305:484–487

243. Varmeh S, Egia A, McGrouther D, et al (2011) Cellular senescence as a possible mechanism for halting progression of keloid lesions. Genes and Cancer 2:1061–1066

244. Vincent AS, Phan TT, Mukhopadhyay A, et al (2008) Human skin keloid fibroblasts display bioenergetics of cancer cells. J Invest Dermatol 128:702–709

245. Wang X, Liu Y, Chen X, et al (2013) Impact of miR-21 on the expression of FasL in the presence of TGF-β1. Aesthetic Surg J 33:1186–1198

246. Wang X, Liu K, Ruan M, et al (2018) Gallic acid inhibits fibroblast growth and migration in keloids through the AKT/ERK signaling pathway. Acta Biochim Biophys Sin 50:1114–1120

247. Wen A, Chua C, Uin S, et al (2011) Keloid fibroblasts are more sensitive to Wnt3a treatment in terms of elevated cellular growth and fibronectin expression. J Dermatol Sci 64:199–209

248. Witt E, Maliri A, McGrouther DA, Bayat A (2008) RAC activity in keloid disease: comparative analysis of fibroblasts from margin of keloid to its surrounding normal skin. Eplasty 8:e19

249. Wong VW, You F, Januszyk M, et al (2014) Transcriptional profiling of rapamycin-treated fibroblasts from hypertrophic and keloid scars. Ann Plast Surg 72:711–719

250. Wu Y, Peng Y, Gao D, et al (2015) Mesenchymal stem cells suppress fibroblast proliferation and reduce skin fibrosis through a TGF-β3-dependent activation. Int J Low Extrem Wounds 14:50–62

251. Wu Y, Zhang Q, Ann DK, et al (2004) Increased vascular endothelial growth factor may account for elevated level of plasminogen activator inhibitor-1 via activating ERK1/2 in keloid fibroblasts. Am J Physiol Cell Physiol 286:C905–C912

252. Xia W, Longaker MT, Yang GP (2005) P38 MAP kinase mediates transforming growth factor-β2 transcription in human keloid fibroblasts. Am J Physiol Regul Integr Comp Physiol 290:R501–R508

253. Xia W, Kong W, Wang Z, et al (2007) Increased CCN2 transcription in keloid fibroblasts requires cooperativity between AP-1 and SMAD binding sites. Ann Surg 246:886–895

254. Xia W, Phan T-T, Lim IJ, et al (2004) Complex epithelial-mesenchymal interactions modulate transforming growth factor-beta expression in keloid-derived cells. Wound Repair Regen 12:546–556

255. Xin Y, Wang X, Zhu M, et al (2017) Expansion of CD26 positive fibroblast population promotes keloid progression. Exp Cell Res 356:104–113

256. Xue H, McCauley RL, Zhang W (2000) Elevated interleukin-6 expression in keloid fibroblasts. J Surg Res 89:74–77

257. Yagi Y, Muroga E, Naitoh M, et al (2013) An ex vivo model employing keloid-derived cell-seeded collagen sponges for therapy development. J Invest Dermatol 133:386–393

258. Yamawaki S, Naitoh M, Kubota H, et al (2018) HtrA1 is specifically up-regulated in active keloid lesions and stimulates keloid development. Int J Mol Sci 19:1–12

259. Yan L, Cao R, Liu Y, et al (2016) MiR-21-5p links epithelial-mesenchymal transition phenotype with stem-like cell signatures via AKT signaling in keloid keratinocytes. Sci Rep 6:1–11

260. Yan L, Cao R, Wang L, et al (2015) Epithelial-mesenchymal transition in keloid tissues and TGF-β1-induced hair follicle outer root sheath keratinocytes. Wound Repair Regen 23:601–610

261. Yeh F, Shen H, Lin M, et al (2006) Keloid-derived fibroblasts have a diminished capacity to produce prostaglandin E2. Burns 32:299–304

262. Yeh FL, Shen H Der, Tai HY (2009) Decreased production of MCP-1 and MMP-2 by keloid-derived fibroblasts. Burns 35:348–351

263. Yoshimoto H, Ishihara H, Ohtsuru A, et al (1999) Overexpression of insulin-like growth factor-1 (IGF-I) receptor and the invasiveness of cultured keloid fibroblasts. Am J Pathol 154:883–889

264. Younai S, Nichter LS, Wellisz T, et al (1994) Modulation of collagen synthesis by transforming growth factor-β in keloid and hypertrophic scar fibroblasts. Ann Plast Surg 33:148–154

265. Younai S, Venters G, Vu S, et al (1996) Role of growth factors in scar contraction: an in vitro analysis. Ann Plast Surg 36:495–501

266. Yu D, Shang Y, Yuan J, et al (2016) Wnt/β-catenin signaling exacerbates keloid cell proliferation by regulating telomerase. Cell Physiol Biochem 39:2001–2013

267. Yu H, Bock O, Bayat A, et al (2006) Decreased expression of inhibitory SMAD6 and SMAD7 in keloid scarring. J Plast Reconstr Aesthetic Surg 59:221–229

268. Zhang G-Y, Wu L-C, Liao T, et al (2016) Altered circulating endothelial progenitor cells in patients with keloid. Clin Exp Dermatol 41:152–155

269. Zhang G, Gao W, Li X, et al (2009) Effect of camptothecin on collagen synthesis in fibroblasts from patients with keloid. Ann Plast Surg 63:94–99

270. Zhang Q, Oh CK, Messadi D V., et al (2006) Hypoxia-induced HIF-1 α accumulation is augmented in a co-culture of keloid fibroblasts and human mast cells: involvement of ERK1/2 and PI-3K/Akt. Exp Cell Res 312:145–155

271. Zhang Q, Wu Y, Ann DK, et al (2003) Mechanisms of hypoxic regulation of plasminogen activator inhibitor-1 gene expression in keloid fibroblasts. J Invest Dermatol 121:1005–1012

272. Zhang Q, Wu Y, Chau CH, et al (2004) Crosstalk of hypoxia-mediated signaling pathways in upregulating plasminogen activator inhibitor-1 expression in keloid fibroblasts. J Cell Physiol 199:89–97

273. Zhang Q, Yamaza T, Kelly AP, et al (2009) Tumor-like stem cells derived from human keloid are governed by the inflammatory niche driven by IL-17/IL-6 axis. PLoS One 4:e7798

274. Zhang Y, Zhang Z, Liu Y, et al (2015) Requirement of Gαi1/3-Gab1 signaling complex for keratinocyte growth factor-induced PI3K-AKT-mTORC1 activation. J Invest Dermatol 135:181–191

275. Zhang ZF, Zhang YG, Hu DH, et al (2011) Smad interacting protein 1 as a regulator of skin fibrosis in pathological scars. Burns 37:665–672

276. Zhang Z, Garron TM, Li XJ, et al (2009) Recombinant human decorin inhibits TGF-β1 induced contraction of collagen lattice by keloid fibroblasts. Wounds 21:47–56

277. Zhao J, Zhong A, Friedrich EE, et al (2017) S100A12 induced in the epidermis by reduced hydration activates dermal fibroblasts and causes dermal fibrosis. J Invest Dermatol 137:650–659

278. Zhao YX, Zhang GY, Wang AY, et al (2017) Role of homeodomain-interacting protein kinase 2 in the pathogenesis of tissue fibrosis in keloid-derived keratinocytes. Burn Surg Res 79:546–551
