# Supplementary material for: The Keloid Disorder: Heterogeneity, Histopathology, Mechanisms and Models
Source: Front Cell Dev Biol. 2020 May 26;8:360. doi: 10.3389/fcell.2020.00360 (PMC7264387; doi:10.3389/fcell.2020.00360)
Supplement: Supplementary file 4 [file Table_4.DOCX]

**Supplemental table 4A.** Human in vivo keloid models

| **Method** | **Keloid scar parameters** | **References** |
| --- | --- | --- |
| **Non-invasive methods** | | |
| Multiphoton microscopy | live imaging: max. visible depth 20 μm  - much thicker, wavy collagen bundles  - collagen organised in swirls of numerous, closely bound fibrils;  random orientation | [16] |
| Multiphoton microscopy | collagen organisation scoring:  - ↑ collagen/elastin content  - ↓ collagen fiber-bundles | [15]† |
| Dermoscopy | identifies vascular structures:  - in 90% of keloids with arborizing, linear irregular or comma-  shaped structures | [102] |
| Diffuse reflectance spectroscopy | measures optical properties: probing depth < 1 mm  - ↑ average collagen, ↑ deoxygenated Hb with ↓ O_2_ saturation,  ↓ scattering coefficient, normal melanin volume | [95] |
| 3D photography | 3D imaging:  - 3D scar volume measurement combined with correcting formula  correlated highly with calculated volume measurement using  deformable modelling compound | [1, 5] |
| Stratum corneum function | bioengineering measurements:  - continued ↑ TEWL and high-frequency conductance  - in vivo water sorption-desorption test: ↑ uptake of water  - SC turnover time: ↑ | [89]^^ |
| Optical coherence tomography | imaging of skin architecture:  - disarray of hyperreflective areas, heterogenous morphology, no  vascular supply or lymphatics in dermis | [82] |
| Laser speckle contrast imaging | assessment mean perfusion:  - ↑ perfusion within keloids and adjacent skin | [53] |
| Digital reactive hyperaemia - Peripheral arterial tonometry | measures endothelial function:  - worse reactive hyperaemia and ↑ arterial stiffness | [71] |
| High-frequency ultrasonography | discriminates Nskin from Hscar/Kscar:  - echo-poor image in Hscar/Kscar, with ↓ densitometric values | [9]^^ |
| High-resolution ultrasonography | scar assessment after treatment:  - ↑ lesion thickness, variable echogenicity, vascularity − | [22]^^ |
| Ultrasound shear wave elastography | estimate of tissue stiffness:  - ↑ in active keloid areas with whorled, hyalinized collagen  - ↓ mature keloid areas with parallel collagen fibers | [7] |
| Allergy questionnaire | - ↑ allergy symptoms in keloid-scar formers | [88]^^ |
| Symptom and small nerve fiber function testing | (thermo)sensory testing:  - itch at keloid edge and surrounding skin, pain at keloid centre  - thermal probe: abnormal ↑ thermosensory thresholds to warmth, cold, heat pain  - fine filament brush strokes: 43% allodynia, 14% alloknesis | [35] |
| **Invasive methods** | | |
| FDG-PET | transmission scanning after FDG injection:  - ↑ FDG uptake in keloids (altered glucose metabolism) | [75] |
| Serial biopsies | evaluated keloid development over 1 mo – 3 yrs:  - ↑ TGF-β1, ↑ collagen I, ↑ collagen III and then ↓ after 3 mo | [51] |
| Wounding keloid-formers  (to study keloid development with serial biopsies from day 18-12 mo) | 15 cm incision (to subcutis) and 10 x 15 cm excision (3mm thick) on abdominal skin of known Hscar/Kscar-formers:  - flattened epithelium  - perivascular nodules; fibroplasia persists, ↑ and invades  - very hard consistency, large size, evolution over months-years | [55] |
| **Computational models with in vivo data input** | | |
| Finite element method + tracing/scanning of keloids | calculates stresses/stretching tension:  - keloid expansion occurs in direction it is pulled | [3, 70] |
| Finite element method + human mechanical testing +  existing literature | analyses distribution of mechanical stress and strain:  - ↑ position-associated mechanical loading in keloid-prone sites  - mechanical stress is ↑↑ in Kscar, mechanical strain is ↑↑ in sNskin | [19] |

**Supplemental table 4A.** Overview of in vivo testing of keloid scars, evaluating intrinsic keloid abnormalities. Legend; ↑: increased; ↓: decreased; ^^: the same model also constructed for hypertrophic scars, only qualitative dissimilarities noted here; † although tested on ex vivo explants, application was meant for non-invasive in vivo imaging and therefore included in this table. Abbreviations listed in alphabetical order; FDG: fluorine-18-fluorodeoxyglucose; Hb: haemoglobin; Hscar: hypertrophic scar; Kscar: keloid scar; mo: month(s); Nskin: normal skin; O_2_: oxygen; PET: positron emission tomography; sNskin: normal skin directly surrounding keloid scars; TEWL: transepidermal water loss (stratum corneum function); TGF-β1: transforming growth factor beta 1; yrs: years.

**Supplemental table 4B.** Human explant keloid models

| **Material** | **Culture system** | **Keloid scar parameters** | **References** |
| --- | --- | --- | --- |
| **Keloid explants cultured submerged** | | | |
| Keloid explant  (4-6 mm Ø) | submerged  (10 days) | - normal collagen degradation  - normal collagenase production | [57]^^ |
| Keloid explant  (5 mm width) | submerged  (28 days) | - use of adeno-associated virus vector to transfer and cause  expression of *lacZ* reporter gene, no specific keloid markers  studied | [54] |
| Keloid explant  (0.5-1 mm^2^) | submerged  (25 days) | - contraction with ↓ size (Nskin size unchanged) | [83] |
| Keloid dermis  (2 mm Ø) | submerged  (3 days) | transfected with relaxin-expressing adenovirus:  - ↓ collagen I and III, fibronectin, elastin | [36] |
| Keloid dermis  (3 x 2 x 2 mm) | submerged  (7 days) | + gallic acid: ↓ KF migration, ↓ collagen accumulation,  ↓ angiogenesis markers (CD31, VEGF, VEGFR) | [98] |
| Keloid dermis  (0.5 mm Ø) | submerged  short incubation  (18 hrs) | - normal cellularity (↑ in Hscar)  - ↓ general protein synthesis (↑ in Hscar)  - ↑ collagen synthesis rate  - ↑ ratio of collagen/non-collagen protein synthesis | [17]^^ |
| **Keloid explants cultured air-exposed** | | | |
| Keloid explant  (10 x 10 mm) | air-exposed  (7 days) | + TIMP-2 intradermal injection:  - ↓ dermal thickness, collagen bundle thickness, α-SMA | [20] |
| Keloid explant  (1 cm^2^) | air-exposed  (7 days) | - ↑ dermal thickness, collagen bundle thickness, α-SMA  - normal TIMP-1, MMP-1, MT1-MMP | [20] |
| Keloid explant  (4 mm Ø) | on plastic  - air-exposed  - submerged  (4-6 wk) | - inferior preservation vs. collagen gel method:  rapid degeneration & epidermal detachment after 1 week | [21] |
| **Keloid explants cultured air-exposed in collagen gel** | | | |
| Keloid explant  (6 mm Ø) | in collagen gel  air-exposed  (7 days) | - conservation of morphology  - epidermis: detached with few apoptotic cells, ↑ proliferation  (↑ apoptosis, proliferation + in Hscar)  - dermis: ↑ elastin (+ in Hscar), ↑ collagen I and III, ↓ MMP-3 | [56]^^ |
| Keloid explant  (3-6 mm Ø) | in collagen gel  - air-exposed  - submerged  (6 wk) | - least cytotoxicity, most proliferation in 4mm Ø cultured air-  exposed in William’s E medium (epidermal detachment in  submerged explants)  - preservation of keloid architecture, vasculature, RNA  content, collagen I and III expression  - expression of Kscar markers: CD3, CD20, CD31, CD34,  CD56, tryptase, Langerin, vimentin, neutrophil elastase,  CTGF and collagen | [8] |
| Keloid explant  (4 mm Ø) | in collagen gel  - air-exposed  - submerged  (4-6 wk) | - superior preservation compared with plastic method:  fewer abnormal cells, no epidermal detachment but loss of  LCs; preservation of majority of endothelial cells,  fibroblasts, mast cells; continued collagen I expression  - no differences between serum-free and serum-containing  media in air-exposed explants  - no morphological differences between air-exposed and  submerged explants maintained in serum-free media | [21] |
| **Keloid explants, culturing conditions unclear** | | | |
| Keloid explant  (4 mm^2^) | culturing unclear  (7 days) | - ↓ elastin | [26] |
| Keloid explant  (4 mm^2^) | culturing unclear  (10 days) | - ↓ elastin and elastic fibers  - densely packed collagen bundles | [62]^^ |
| **Use of fresh keloid tissue, uncultured** | | | |
| Keloid fresh tissue  (size unknown) | used within 1hr post-operatively | - ↑ O_2_ consumption rate in keloid hypertrophic scars | [28]† |

**Supplemental table 4B.** Available keloid explant models are listed in this table. When the keloid explant was specifically described as ‘keloid dermis’ in the paper, it is listed as such under the column ‘materials’. Legend; ↑: increased; ↓: decreased; ^^: the same model also constructed for hypertrophic scars, only qualitative dissimilarities noted here; † no distinction made between keloid and hypertrophic scars. Abbreviations listed in alphabetical order; α-SMA: alpha smooth muscle actin; hrs: hours; Hscar: hypertrophic scar; KF: keloid fibroblast; Kscar: keloid scar; lacZ: β-galactosidase; MMP: matrix metalloproteinase; Nskin: normal skin; O_2_: oxygen; TIMP: tissue inhibitor of metalloproteinases; VEGF: vascular endothelial growth factor; VEGFR: vascular endothelial growth factor receptor.

**Supplemental table 4C.** Animal in vivo keloid models

| **Material** | **Culture system** | **Keloid scar parameters** | **References** |
| --- | --- | --- | --- |
| **Implantation of full thickness human keloid explants** | | | |
| Keloid explants  (5 x 10 x 5 mm) | athymic nude mice  *subcutaneous implantation between shoulders*  (246 days) | - GAG distribution over time:  ↓ uronic acid, ↓ of initially increased chondroitin-4-  sulphate, ↑ dermatan sulphate, ↑ hyaluronic acid  - implant weight initial ↑ then ↓ | [86]^^ |
| Keloid explants  (5 x 10 x 5 mm) | athymic nude mice  *subcutaneous dorsal implantation*  (90 days) | - fibroblasts retained, no lymphocytes  + pirfenidone feeding: ↓ weight, GAGs unaltered, keloidal  collagen +  + triamcinolone injection: ↓ weight, ↓ GAGs, ↓ fibroblasts,  ↑ disorganisation of collagen bundles | [87] |
| Keloid explants  (8 mm Ø, 4 mm depth) | homozygote nude mice  *sutured onto back*  (3 mo) | - no infection or rejection  - partial/total depigmentation at 1 mo post-wounding  - keloidal collagen + | [80] |
| Keloid explants  (5 mm cubes) | BALB/c nude mice  *sutured onto dorsum*  (35 days) | injection of chondroitinase ABC (CS degradation):  - ↓ volume  - ↑ elastic fibers and fibrillin | [29] |
| Keloid explants  (10 x 10 x 5 mm) | athymic nude rats  *implanted into*  *sandwich-island flaps*  (3 wk maturation + 120 days) | injection of TGF-β2:  - at 30 days: ↑ endogenous TGF-β2, collagen I and III  - at 120 days: no difference in collagen levels | [99]^^ |
| Keloid explants  (6 x 4 x 3 mm) | athymic nude mice  *subcutaneous intraperitoneal implantation* (6 wk) | injection of BAMBI:  - ↓ volume, collagen I ratio, fiber size | [51] |
| Keloid explants  (8 mm Ø, 4 mm depth) | athymic mice  *subcutaneous dorsal implantation* (4 wk) | - injection of ASC-supernatant: shrinkage, ↓ inflammation  (CD68), ↓ vascularity, thin collagens  - thick collagen bundles with multiple orientations | [52]‡ |
| Keloid explant  (2 mm Ø) | hamsters  *subepithelial implantation*  *in cheek pouch*  (168 days) | - 26% fragments not integrated  - epidermis absent in 39% of integrated fragments  - vascularisation present after 5 days  - dark brown pigmentation visible | [27] |
| **Implantation of de-epithelialized human keloid explants** | | | |
| Keloid dermis  (5 x 8 x 5 mm) | athymic nude mice  *subcutaneous implantation between shoulders*  (2-246 days) | - no rejection, infection or inflammation; necrosis once  - nodules and cellular character maintained  - vascularisation +, occluded microvessels  - ↓ in implant size over time | [33]^^ |
| Keloid dermis  (5 x 8 x 5 mm) | athymic nude mice  *suprascapular subcutaneous implantation* (2-246 days) | - no rejection, viable implant, morphology intact  - peripheral vascularisation with anastomoses between  mice and implant, not into/throughout implant | [33]^^ |
| Keloid dermis  (8 x 8 x 5 mm) | BALB/c nude mice  *subcutaneous implantation*  *in axillary region* (16 days) | transfected with shRNA-HSP47 plasmids:  - ↓ HSP47, collagen I, volume | [12] |
| Keloid dermis  (1 μm slices)  or  Keloid fibroblasts | athymic nude mice *subcutaneous implantation over flank, thorax*  (2 mo) | - raised, firm subcutaneous masses  - dermal and fibroblast implants both resembled native  keloid, except for ↓ cell density  - keloidal collagen + in 88% | [23] |
| Keloid dermis  (4 x 4 x 3 mm) | athymic mice  *subcutaneous dorsal implantation*  (14 wk) | - ↑ volume, stabilising after 8 wk  - abundant collagen fibers, nodular appearance  - keloidal collagen +  + silicone gel sheeting: ↓ volume, ↓ keloidal collagen | [39] |
| Keloid dermis  (1 mm^3^) | athymic nude mice  *subcutaneous implantation*  *on thorax*  (8 wk) | - initial growth spurt (4 wk), then slow regression, implant  became oval soft white mass;  - no rejection, infection or cellular degeneration  - vascularisation +  - good take, reasonable histologic conservation with  some loss of collagen density, but collagen bundles  resemble native keloid | [96] |
| Keloid dermis  (5-10 mm) | athymic nude mice  *subcutaneous implantation between shoulders*  (60 days) | - no rejection or necrosis  - implanted tissue retained viability and original histology  - vascularisation +  - GAG distribution similar to native keloid | [85] |

**Supplemental table 4C.** Animal in vivo keloid models – continued

| **Material** | **Culture system** | **Keloid scar parameters** | **References** |
| --- | --- | --- | --- |
| **Implantation of human keloid-derived fibroblast suspension** | | | |
| Keloid fibroblasts | BALB/c nude mice  *injected into back*  (17 days) | - ↑ nodule size  - peripheral vascularisation around nodules  - ↑ collagen fibers | [76] |
| Keloid fibroblasts | athymic nude mice  *injected into back* (7 days) | - development of nodules with ↑ fibronectin, TGF-β1,  fibroblasts, capillary numbers | [40]* |
| Keloid fibroblasts | BALB/c nude mice  *injected into neck*  (128 days) | - formed transplanted tumours | [103] |
| Keloid fibroblasts | SCID mice  *Injected into back*  (14 days) | development of nodules with:  - ↑ weight, neovascularisation, cellularity, inflammatory  cells and endothelial cells, human collagen I, thick  collagen fibers | [24]* |
| **Implantation of human keloid-derived fibroblasts in 3D culture** | | | |
| Keloid fibroblasts  in matrix gel  + KF-supernatant | nude mice  *injected into back*  (6 wk) | - nodules +, nodule size ↑ in 2-hr vs 24-hr supernatant  - similar keloid morphology: dense fibroblasts, arranged  disorderly | [84] |
| Keloid fibroblasts  in hydrogel,  HA/TCP or  Gelfoam  (3 x 3 x 2 mm) | C57BL/6 nude mice  *subcutaneous dorsal implantation*  (8 wk) | - in Gelfoam: ↑ growing tumour-like mass, collagen I +,  but no keloidal collagen  - hydrogel + IL-6: ↑↑ growing tumour-like mass, **keloidal**  **collagen +**, ↑ telomerase activity, ↑ proliferation  - in hydrogel + naïve T-cell infusion: ↑↑ growth  - in HA/TCP: KF generated calcified nodules (bone  regenerative capability  - in hydrogel for serial transplantation to confirm stemness of  KF: connective tissue +, collagen I +, but no tumour | [105] |
| Keloid fibroblasts in PLGA scaffold  (5 × 5 × 0.5 mm) | athymic nude mice  *subcutaneous flank implantation* (180 days) | - continued ↑ implant size with palpable nodule  - ↑ KF, ↑ collagen I, PLGA degeneration, vascularisation +  - ↑ rough endoplasmatic reticulum in KF | [97]*‡ |
| Keloid fibroblasts in collagen sponge  (12 mm Ø) | athymic nude mice  *subcutaneous dorsal implantation* (1 mo) | - ↑ weight | [101]* |
| **Implantation of human (keloid) keratinocytes and keloid fibroblasts** | | | |
| KK/KF mixed-cell suspension in double chamber  (1 cm Ø) | NOG null mice  *sutured onto back*  (12 wk) | - nodules +, macroscopically similar to keloids,  - ↑ thickness, collagen I +, ↑ collagen III,  chaotic collagen fiber orientation, ↑ versican  - N.B. ↑ involucrin in Nskin, Nscar and Kscar models | [90] |
| Skin equivalent KK/KF  (2 x 2 cm) | athymic nude mice  *sutured onto flank*  (14 wk) | - in both constructs: thick disorganized collagen bundles  - KK/deep-KF: ↑ dermal thickness, ↑ collagen I  - KK/superficial-KF: ↑ surface area | [91]‡ |
| Skin equivalent  NK/KF  (2 x 2 cm) | athymic nude mice  *sutured onto flank*  (14 wk) | - in both constructs: thick disorganized collagen bundles  - NK/deep-KF: ↑ collagen I  - NK/superficial-KF: normal collagen I | [91]‡ |
| Skin equivalent KK/KF (1 wk)  (0.8 cm porous polyethylene ring-supported) | athymic nude mice  *sutured onto lower dorsum*  (17 wk) | implantation of plasma/fibrin-based KK/KF construct:  - human cells shown to survive in implant  - ↑ epidermal thickness, sporadic DEJ disruption  - ↑ dermal thickness, foci with ↑ dermal cellularity, ↑ large  collagen bundles, ↑ vascularisation, ↑ PAI-1, ↑ uPAR  - ↑ EMT-markers (TGF-β, FSP-1)  - ↑ (mouse) mφ; some α-SMA + dermal cells  - ↑ p-STAT3 (Tyr705) | [37]  [38] |
| Skin equivalent  NK/KF  (2 cm Ø porous polyethylene ring support) | athymic nude mice  *sutured onto lower dorsum*  (20wk) | KF induce partial keloid scar phenotype in model:  - ↑ overall implant size, collagen I, PAI-1, vascular  recruitment, epidermal thickness  - raised above host skin, mature dense collagen  accumulation  - capable of secondary human cell-dependent repair | [37] |

**Supplemental table 4C.** Animal in vivo keloid models – continued

| **Material** | **Culture system** | **Keloid scar parameters** | **References** |
| --- | --- | --- | --- |
| **Inducing keloid formation by wounding** | | | |
| Keloid inducement | rabbits  *full thickness flank excision*  (21 days) | - ↓ wound surface area over time  - thick bundle of collagen fibers, “some of which had  broad based configurations, reminiscent of keloid” | [2] |
| Keloid inducement | New Zealand rabbits  *full thickness excisional*  *wounds on ear*  (25 days or 8 mo) | - acute rabbit model (25 days) + TA injection:  ↓ scar area and thickness  - chronic rabbit model (8 mo + larger excision):  ↑ collagen, thickness, vascularity; mild chronic inflammation;  irregularly arranged collagen fibers  - no hyalinizing keloid collagen  - model “may parallel hypertrophic scarring in humans” | [63] |
| Keloid inducement | horse limbs  *full thickness excision*  (4 wk) | - ongoing low-grade inflammation in wounds  - granulation tissue present, but no exuberant granulation  tissue resembling keloids | [11] |
| Keloid inducement | horse limbs  *full thickness excision*  (wound closure, 6 wk) | - prolonged inflammatory response, ↑ angiogenesis, ↓  fibroblast organisation  - ↑ osteopontin; ↓ lumican; ↓ ANXA2 and MMP-1  - ↑ β-catenin and PECAM1 in developed exuberant  granulation tissue | [58–61] |
| Keloid inducement | BALB/c nude mice,  Waster rats,  Newsland rabbits  *deep cutaneous incisions + daily scratching irritation*  (7 wk) | - prolonged wound healing duration  - contraction +  - N.B. developed hypertrophic scars instead of keloids | [32] |

**Supplemental table 4C.** Available keloid animal models are listed in this table, note that this only includes in vivo models and excludes animal-derived in vitro models. When the keloid explant was specifically described as de-epithelialised in the paper, it is listed as ‘keloid dermis’ under the column ‘materials’. The culture system lists, from top to bottom, i. which animal is tested, ii. location of keloid implantation or method of keloid inducement, iii. duration of experiment. Legend; +: present, normal expression or values; ↑: increased; −: absent; ↓: decreased; ‡: n=1 keloid; *explant fibroblast cell isolation; unless stated otherwise, fibroblasts were isolated via enzymatic digestion. Abbreviations listed in alphabetical order; α-SMA: alpha smooth muscle actin; ANXA2: annexin A2; BAMBI: bone morphogenetic protein and activin membrane-bound inhibitor (pseudo-receptor of TGF-β1); CS: chondroitin sulphate; EMT: epithelial-mesenchymal transition (EMT); FSP-1: fibroblast-specific protein 1; GAG: glycosaminoglycans; HA/TCP: hydroxyapatite/tricalcium phosphate ceramic powder; HSP47: heat shock protein 47; KF: keloid fibroblast; KK: keloid keratinocyte; MMP: matrix metalloproteinase; mo: month(s); PAI-1: plasminogen activator inhibitor 1; PECAM1: platelet endothelial cell adhesion molecule 1; PLGA: poly(lactic-co-glycolic acid) copolymer; shRNA: small hairpin RNA; TA: triamcinolone acetonide; TGF-β: transforming growth factor beta; uPAR: urokinase receptor; wk: week(s).

**Supplemental table 4D.** Human indirect co-culture keloid models

| **Cell types** | **Culture system** | **Keloid scar parameters** | **References** |
| --- | --- | --- | --- |
| **Keloid keratinocyte monolayer / keloid fibroblast monolayer** | | | |
| Keloid keratinocytes  Keloid fibroblasts | monolayer†  monolayer  (7 days) | - in KF: ↑↑ proliferation, ↓↓ apoptosis, ↑↑ apoptosis  resistance (with ↓↓ active caspase-3), ↑↑ ERK & JNK  phosphorylation, ↑↑ TGF-β1  - in KF: ↑↑ Bcl-2 (vs NK/KF) | [25]* |
| Keloid keratinocytes  Keloid fibroblasts | monolayer  monolayer  (duration unknown) | - ↑ collagen III, ↓ collagen I, normal collagen V, ↑ TGF-β1 (vs  NK/NF)  - ↑↑ SMAD2, ↑↑ SMAD4, ↓↓ SMAD3, ↓↓ SMAD7 | [43] |
| **Keloid epidermis / keloid fibroblast monolayer** | | | |
| Keloid keratinocytes  Keloid fibroblasts | epidermis  monolayer  (15 days) | - in KF: ↑↑ collagen synthesis | [79] |
| Keloid keratinocytes  Keloid fibroblasts | epidermis  monolayer  (12 days) | - ↑↑ CTGF in supernatant  - in KF: ↓↓ CTGF (although normal levels in KK/KF vs NK/NF)  - in KK: CTGF unaffected | [31] |
| Keloid keratinocytes  Keloid fibroblasts | epidermis  monolayer  (12 days) | - ↑↑ HDGF in supernatant  - in KF: HDGF unaffected by co-culture | [74] |
| Keloid keratinocytes  Keloid fibroblasts | epidermis  monolayer  (12 days) | - ↑↑ VEGF in supernatant  - in KF: VEGF unaffected by co-culture  - in KK: normal ↑ VEGF, normal ↓ PLGF | [73] |
| Keloid keratinocytes  Keloid fibroblasts | epidermis  monolayer  (12 days) | - ↑ collagen, ↑ α-SMA, ↑ fibronectin in supernatant (vs NK/NF)  - with mTOR inhibitor: normal ↓ collagen, ↓↓ α-SMA, ↓↓  fibronectin  in supernatant (vs NK/NF) | [72] |
| Keloid keratinocytes  Keloid fibroblasts | epidermis  monolayer  (12 days) | - in KF: ↑↑ TGFβRI/II, SMAD3, SMAD 3 phosphorylation  p-SMAD 2, SMAD 2/3/4 binding complex;  SMAD2/4 unaffected by co-culture | [78] |
| Keloid keratinocytes  Keloid fibroblasts | epidermis  monolayer  (12 days) | - ↑↑ IL-18, caspase-1; ↓↓ IL-10 in supernatant  - in KF: ↑↑ IL-18  - in KK: ↑↑ IL-18 | [18] |
| Keloid keratinocytes  Keloid fibroblasts | epidermis  monolayer  (12 days) | - in KF: ↑↑ proliferation, collagen I/II/III secretion  ↑↑ PI3K and MEK-ERK pathways  - in KF: ↑ collagen I/II/III, laminin β2, fibronectin in supernatant | [45] |
| Keloid keratinocytes  Keloid fibroblasts | epidermis  monolayer  (13 days) | - ↑↑ R-spondin2 in supernatant  - in KK: R-spondin2 unaffected by co-culture  - in KF: ↑↑ R-spondin2 | [14] |
| Keloid keratinocytes  Keloid fibroblasts | epidermis  monolayer  (13 days) | - in KF: ↑↑ proliferation, IGFBP2, IGF-1R, p-MAPK, p-ELK1,  p-MEK1/2  - in KF: ↓↓ IGFBP3/5; IGFBP4, p-Akt, p-Raf unaffected by co-  culture  - normal ↑ IGFBP2, ↑↑ IGFBP4 in supernatant  - IGF-1, IGFBP3 in supernatant unaffected by co-culture | [77] |
| Keloid keratinocytes  Keloid fibroblasts | epidermis  monolayer  (15 days) | - ↑ HGF, c-Met in supernatant (vs NK/NF)  - in KK: normal ↑ HGF, c-Met unaffected by co-culture  - in KF: ↓ c-Met (vs KF monoculture)  - ↑ HGF in supernatant (vs KF monoculture) | [67] |
| Keloid keratinocytes  Keloid fibroblasts | epidermis  monolayer  (15 days) | - in KF: ↑↑ proliferation  - ↑↑ proliferation rate of entire co-culture | [44] |
| Keloid keratinocytes  Keloid fibroblasts | epidermis  monolayer  (15 days) | - in KK: ↑ TGF-β1/3, TGF-βRI; normal TGF-β2 (vs NK/NF)  - in KF: ↑↑ proliferation  - in KF: ↑ collagen I, CTGF, IGF-2/M6P receptor, TGF-β1,  TGF-β2, TGF-βRI, SMAD 2 (vs NK/NF)  - ↑ activated and total TGF-β in supernatant (vs NK/NF) | [100] |
| Keloid keratinocytes  Keloid fibroblasts | epidermis  monolayer  (12 days) | - normal ↑ collagen I and III in supernatant  - in KF: normal ↑ insoluble collagen I and III  - in KF: normal ↑ collagen I, but not collagen III mRNA | [46] |

**Supplemental table 4D.** Human indirect co-culture keloid models − continued

| **Cell types** | **Culture system** | **Keloid scar parameters** | **References** |
| --- | --- | --- | --- |
| **Keloid epidermis / keloid fibroblast monolayer - continued** | | | |
| Keloid keratinocytes  Keloid fibroblasts | epidermis  monolayer  (15 days) | - in KK: normal ↑ STAT3, ↓↓ p-STAT3 (Tyr705)  - in KF: ↑↑ STAT3, ↓↓ p-STAT3 (Tyr705)  - in supernatant (vs NK/NF): ↓ CCL15, IGFBP2;  ↑ ANG, OSM, VEGF, IGFBP1, OPG, TGF-β2;  normal IL-6, IL-8, MCP-1, TIMP-1, TIMP-2 | [42]‡ |
| Keloid keratinocytes  Keloid fibroblasts | epidermis  monolayer  (duration unknown) | - normal ↑ stem cell factor in supernatant  - ↑↑ c-Kit in supernatant (vs NK/NF)  - in KK: normal c-Kit; in KF: normal c-Kit  - in KK and KF total: ↑ TNF-α convertase, phosphorylated c-Kit  (vs NK/NF) | [64] |
| Keloid keratinocytes  Keloid fibroblasts | epidermis  monolayer  (duration unknown) | - in KF: ↓ Sp1 (vs NK/NF) | [66] |
| Keloid keratinocytes  Keloid fibroblasts | epidermis  monolayer  (duration unknown) | - in KK: ↑↑ FGF-2  - in KF: reduced ↑ FGF-2, syndecan or decorin unaffected by  co-culture  - ↑ syndecan-2 in supernatant (vs KF monoculture) | [68] |
| **Heterotypic co-culture with keloid keratinocytes or keloid fibroblasts** | | | |
| Keloid keratinocytes  Normal fibroblasts | monolayer  monolayer  (various: 7-12 days) | effect on NF:  - ↑ proliferation, ↓↓ apoptosis  - ↑↑ TGF-β1, ERK and JNK phosphorylation | [25]*†  [25]*† |
| Keloid keratinocytes  Normal fibroblasts | epidermis  monolayer  (various: 12-15 days) | effect on NF:  - ↑ collagen synthesis  - ↑ collagen I-III production  - ↓↓ intracellular collagen I and III  - secretion of collagen in keloid-like manner  - ↑↑ proliferation  - ↑ PI3K and MEK-ERK pathways  - ↓↓ intracellular CTGF, ↑↑ CTGF secretion  - ↑↑ intracellular R-spondin2  - ↑↑ intracellular IGFBP-3, ↑ downstream IGF signalling  effect on KK:  - ↑ intracellular TGF-β3, ↑↑ intracellular VEGF  in supernatant:  - ↑↑ collagen I and III  - ↑↑ activated TGF-β, ↑↑ VEGF  - ↑↑ R-spondin2  - ↑↑ IGFBP-3 | [79]  [45]  [46]  [46]  [44, 45, 77, 100]  [45]  [31]  [14]  [77]  [73, 100]  [46]  [73, 100]  [14]  [77] |
| Normal keratinocytes  Keloid fibroblasts | monolayer  monolayer  (various: 7-12 days) | effect on KF:  - ↑↑ proliferation, ↓↓ apoptosis  - ↑↑ TGF-β1, ERK and JNK phosphorylation | [25]*†  [25]*† |
| Normal keratinocytes  Keloid fibroblasts | epidermis  monolayer  (various: 7-15 days) | effect on KF:  - ↑↑ proliferation  - ↑ collagen synthesis  - ↓ intracellular collagen I and III  - ↑↑ intracellular TGF-β1, IGF2R/M6P  - ↓↓ intracellular CTGF, ↑ CTGF secretion  - ↑↑ intracellular R-spondin2  - ↑↑ intracellular IGFBP-2/4, ↓↓ IGFBP-3/5, ↑ downstream  IGF signalling  in supernatant:  - ↑ collagen I, ↓ collagen III  - ↑↑ total TGF-β, ↑↑ R-spondin2 | [44, 77, 100]  [79]  [46]  [100]  [31]  [14]  [77]  [46]  [14, 100] |
| HaCaT keratinocytes  Keloid fibroblasts | monolayer  monolayer  (6-8 days) | effect on HaCaT keratinocyte cell line:  - induce ↑↑ epithelial-mesenchymal transition (vimentin) | [34] |

**Supplemental table 4D.** Human indirect co-culture keloid models − continued

| **Cell types** | **Culture system** | **Keloid scar parameters** | **References** |
| --- | --- | --- | --- |
| **Co-culture with keloid-derived immune cells** | | | |
| CD4+ Tregs  Keloid fibroblasts | monolayer  monolayer  (6 days) | CD4+/FOXP3+ Tregs form keloid-formers’ PBMC: induced  - ↓ collagen I, TGF-β, α-SMA in KF  - ↑ IL-6 in KF | [69] |
| CD14+ monocytes  Normal fibroblasts | monolayer  monolayer  (48 hrs) | peripheral CD14+ monocytes: induced  - ↑ proliferation, MCP-1 secretion in NF | [41]* |
| CD14+ macrophages  CD3/4+ T-cells | monolayer  monolayer  (12 days) | CD14+ macrophages (from keloid tissue): induced  - ↑ FOXP3 in T-lymphocytes (from peripheral blood) | [30] |
| **Co-culture with other non-(epi)dermal cell types** | | | |
| MSCs  Keloid fibroblasts | monolayer  monolayer  (2 days) | effects on MSCs:  - ↑ migration toward underlying keloid fibroblasts  - ↑ myofibroblast-like ultrastructural changes (contractive  microfilaments, rough ER, thick collagen secreted granules)  - ↑ fibronectin | [4]* |
| BM-MSCs  Keloid fibroblasts | monolayer  monolayer  (7 days) | effects on keloid fibroblasts:  - ↑ PAI-1, TGF-β2; TGF-β1 unaffected by co-culture  - ↓ TGF-β3; decorin unaffected by co-culture | [6]* |
| Hair follicle cells  Keloid fibroblasts | monolayer  monolayer  (5 days) | effects on keloid fibroblasts:  - ↑ conversion into adipocytes | [81] |
| **Effect of co-culture with keloid keratinocyte / keloid fibroblast supernatant** | | | |
| KK/KF  Keloid fibroblasts | supernatant  collagen gel  (48 hrs) | - ↑↑ contraction  - with KK or KF monoculture supernatant: slight ↑ contraction | [65] |
| KK/KF  Keloid fibroblasts | supernatant  monolayer  (48 hrs) | - ↑ α-SMA | [65] |
| KK/KF  HUVECs | supernatant  monolayer  (44 hrs) | - ↑ proliferation (vs non-supernatant culture)  - ↑ differentiation into 3D capillary-like structures (vs non-  supernatant culture) | [73] |
| KK/KF or KK  MEF-SMAD null cells | supernatant or epidermis  collagen gel  (3 days) | KK/KF supernatant and KK co-culture have the same effects:  - in MEF-SMAD null cells in collagen gel: ↑ contraction | [78] |
| KK/KF or KK  MEF-SMAD null cells | supernatant or epidermis  monolayer  (3 days) | KK/KF supernatant and KK co-culture have the same effects:  - in MEF-SMAD null cells monolayer:  ↑ proliferation, collagen synthesis | [78] |

**Supplemental table 4D.** Overview of published indirect co-culture systems with keloid-derived cells. The column ‘culture system’ includes the duration of the experiments and includes the time it takes to prepare the individual monocultures prior to co-culture as well as the time needed for any experimental manipulation. Keloid scar parameters of the indirect co-cultures are presented as compared with monoculture control conditions, unless specified otherwise; therefore normal ↑ / ↓: upregulation / downregulation in co-cultures compared with monocultures, no differences between normal skin (NK/NF) or keloid (KK/KF) experimental groups; ↑↑ / ↓↓: increased upregulation / downregulation in keloid homotypic (KK/KF) or heterotypic (KK/NF or NK/KF) co-cultures compared with monocultures and compared with normal homotypic (NK/NF) co-cultures. Expression of keloid scar parameters was further defined as either extracellular (‘in supernatant’) or endogenous (‘in KF’ or ‘in KK’). Legend; +: present, normal expression or values; ↑: increased; −: absent; ↓: decreased; *explant fibroblast cell isolation; unless stated otherwise, fibroblasts were isolated via enzymatic digestion; ‡: n=1 keloid; †: keratinocytes were seeded onto a normal fibroblast feeder layer lethally treated with mitomycin C. Abbreviations are listed in alphabetical order; α-SMA: alpha smooth muscle actin; Bcl-2: B-cell lymphoma 2; CCL15: C-C motif chemokine ligand 15; c-Kit: receptor tyrosine kinase, also known as CD117 (embryonic stem cell marker); c-Met: hepatocyte growth factor receptor; CTGF: connective tissue growth factor; ERK: extracellular-signal-regulated kinase; FGF-2: fibroblast growth factor 2; FOXP3: forkhead box P3; HaCaT: immortalized human keratinocytes; HDGF: hepatoma-derived growth factor; HGF: hepatocyte growth factor; hrs: hours; IL: interleukin; IGF-1: insulin-like growth factor 1; IGFBP: insulin-like growth factor binding protein; IGF-2(R)/M6P: insulin-like growth factor type 2 (receptor)/mannose-6-phosphate; JNK: c-Jun N-terminal kinases; KF: keloid fibroblast; KK: keloid keratinocyte; MCP-1: monocyte chemoattractant protein 1; MEF: mouse embryo fibroblasts; TNF-α convertase: tumour necrosis factor-α converting enzyme; MEK-ERK: mitogen-activated protein kinase-extracellular-signal-regulated kinase; mTOR: mammalian target of rapamycin; NF: normal fibroblast; NK: normal keratinocyte; OPG: osteoprotegerin; OSM: oncostatin M; PAI-1: plasminogen activator inhibitor 1; p-Akt: phosphorylated Akt pathway; PBMCs: peripheral blood mononuclear cells; p-ELK1: phosphorylated ETS like1 pathway; PI3K: phosphoinositide 3-kinases; PLGF: placental growth factor; p-MAPK: phosphorylated mitogen-activated protein kinase pathway; p-MEK1/2: phosphorylated mitogen-activated protein kinase 1/2 pathway; p-Raf: phosphorylated rapidly accelerated fibrosarcoma pathway; p-STAT3 (Tyr705): Tyr phosphorylated (activated) STAT3; SMAD: mothers against decapentaplegic homolog 1 (Drosophila), major effectors of TGF-β signalling; Sp1: transcription factor, regulates several ECM promoters; TGF-β: transforming growth factor beta; TGFβR: transforming growth factor beta receptor; TIMP: tissue inhibitor of metalloproteinase; Tregs: regulatory T-cells; VEGF: vascular endothelial growth factor; WJ-MSCs: human umbilical cord Wharton’s jelly-derived mesenchymal stem cells; wk: week(s); N.B. any additional information on parameters listed in abbreviations are all derived from cited literature in table.

**Supplemental table 4E.** Human direct co-culture keloid models

| **Cell types** | **Culture system** | **Keloid scar parameters** | **References** |
| --- | --- | --- | --- |
| **Direct monolayer co-cultures** | | | |
| Keloid keratinocytes  Keloid fibroblasts | mixed monolayer  (12 weeks) | - ↑ keloid keratinocytes and fibroblasts  - continued ↑ keloid fibroblasts | [32] |
| MSCs  Keloid fibroblasts | monolayer  monolayer  (24 hrs) | effects on MSCs:  - ↑↑ myofibroblast-like ultrastructural changes  (abundant RER, collagen-like fiber secretion, actin-type  microfilaments) | [4]* |
| HMC-1  Keloid fibroblasts | mast cell monolayer  monolayer  (16 hrs in hypoxia) | - in KF: ↑↑ HIF-1α, VEGF  - effect also observed in KF cultured with HMC-1  membrane fragments in hypoxia  - effect not observed in indirect co-cultures | [104] |
| HUVECs  Keloid fibroblasts | seeded on  KF- Matrigel^®^ feedlayer  (4 hrs) | - ↑ HUVEC tube formation | [93] |
| **Heterotypic direct 3D co-cultures** | | | |
| Normal keratinocytes  Keloid fibroblasts  (6.5 mm Ø) | epidermis  collagen gel  (14-16 days) | after full thickness incisional wounding of constructs:  - cell migration into wound, normal contraction,  ↑ collagen deposition | [94] |
| Normal keratinocytes  Keloid fibroblasts  (24 mm Ø) | epidermis  collagen gel  (28 days) | - ↑ epidermal contraction, epidermal thickness,  ↑ dermal contraction, collagen density, dermal  thickness, α-SMA organization | [10] |
| Neonatal keratinocytes  Keloid fibroblasts  (15.6 mm Ø) | epidermis  collagen gel  (20 days) | - ↑ contraction, live-dead ratio (viability)  - ↑ collagen density (thicker and denser collagen fibers,  less organized) | [13] |
| Normal keratinocytes  Keloid fibroblasts  (2 x 2 cm) | epidermis  collagen-GAG  polymer substrate  (16 days) | - 2 constructs made: NK/deep-KF; NK/superficial-KF  - no significant differences in collagen I or contraction | [91]** |
| **Homotypic direct 3D co-cultures** | | | |
| Keloid keratinocytes  Keloid fibroblasts  (2 x 2 cm) | epidermis  collagen-GAG  polymer substrate  (16 days) | - 2 constructs made: KK/deep-KF; KK/superficial-KF  - no significant differences in collagen I or contraction | [91]** |
| Keloid keratinocytes  Keloid fibroblasts  (20 cm^2^) | epidermis  collagen-GAG  polymer sponge  (14 days) | - ↑ collagen I, periostin  - ↓ MMP-1, MMP-3  - 1 wk after wounding: variable keratinocyte migration into  wound edge, ↑ collagen deposition, ↑↑ periostin,  ↑↑ collagen I, MMP1/3 unaffected | [92] |
| Keloid keratinocytes  Keloid fibroblasts  (0.8 cm Ø porous polyethylene ring support) | epidermis  plasma-fibrin matrix  (1wk) | - ↑ collagen I, collagen organisation, dermal thickness,  cellularity | [37]** |
| Keloid keratinocytes  Keloid fibroblasts  + keloid monocytes | epidermis  collagen-elastin matrix  (5wk) | - non-significant trend of ↑ dermal thickness  - persistence of CD34−/α-SMA+/p16+ abnormal scar  phenotype***  - ↓ *COL4A2*, *HAS1*, *MMP3* gene expression  - ↓ HGF secretion  - heterogeneity within keloids: central deep KF generated  most aggressive keloid phenotype  - no effect on keloid phenotype in keloid models, but  ↑ M2 macrophage differentiation in both normal and  keloid monocytes co-cultured with their respective skin  models | [48]  [50]  [49] |

**Supplemental table 4E.** Overview of direct co-culture keloid models. Legend; ↑: increased; ↓: decreased; *explant fibroblast cell isolation; unless stated otherwise, fibroblasts were isolated via enzymatic digestion; ** evaluation of keloid full thickness skin equivalents prior to implantation into an animal model; *** this abnormal scar phenotype was previously established [47] in ex vivo keloids, it should be noted that CD34 was uniformly absent from all skin models including normal skin. Abbreviations listed in alphabetical order; α-SMA: alpha smooth muscle actin; *COL4A2*: collagen type IV alpha 2 chain; GAG: glycosaminoglycan; *HAS1*: hyaluronan synthase 1; HIF-1α: hypoxia-inducible factor 1 alpha; HMC-1: human mast cell line; hrs: hours; HUVEC(s): human umbilical vein endothelial cell(s); KF: keloid fibroblast; KK: keloid keratinocyte; MMP: matrix metalloproteinase; *MMP3*: matrix metalloproteinase 3; MSCs: mesenchymal stem cells; NK: normal keratinocytes; PAI-2: plasminogen activator inhibitor 2; RER: rough endoplasmatic reticulum; TGF-β1: transforming growth factor beta 1; VEGF: vascular endothelial growth factor; wk: week(s).

**References**

1. van der Aa T, Verhiel SHWL, Erends M, et al (2015) A simplified three-dimensional volume measurement technique in keloid scars: validity and reliability. J Plast Reconstr Aesthetic Surg 68:1574–1580

2. Aderounmu AO, Omonisi AE, Akingbasote JA, et al (2013) Wound-healing and potential anti-keloidal properties of the latex of Calotropis Procera (Aiton) Asclepiadaceae in rabbits. Afr J Tradit Complement Altern Med 10:574–579

3. Akaishi S, Akimoto M, Ogawa R, Hyakusoku H (2008) The relationship between keloid growth pattern and stretching tension: visual analysis using the finite element method. Ann Plast Surg 60:445–451

4. Akino K, Akita S, Yakabe A, et al (2008) Human mesenchymal stem cells may be involved in keloid pathogenesis. Int J Dermatol 47:1112–1117

5. Ardehali B, Nouraei SAR, Van Dam H, et al (2007) Objective assessment of keloid scars with three-dimensional imaging: quantifying response to intralesional steroid therapy. Plast Reconstr Surg 119:556–561

6. Arno AI, Amini-Nik S, Blit PH, et al (2014) Effect of human Wharton’s jelly mesenchymal stem cell paracrine signaling on keloid fibroblasts. Stem Cells Transl Med 3:299–307

7. Aya R, Yamawaki S, Yoshikawa K, et al (2015) The shear wave velocity on elastography correlates with the clinical symptoms and histopathological features of keloids. Plast Reconstr Surg - Glob Open 3:e464

8. Bagabir R, Syed F, Paus R, Bayat A (2012) Long-term organ culture of keloid disease tissue. Exp Dermatol 21:376–381

9. Bessonart MN, Macedo N, Carmona C (2005) High resolution B-scan ultrasound of hypertrophic scars. Ski Res Technol 11:185–188

10. Butler PD, Ly DP, Longaker MT, Yang GP (2008) Use of organotypic coculture to study keloid biology. Am J Surg 195:144–148

11. Celeste CJ, Deschene K, Riley CB, Theoret CL (2010) Regional differences in wound oxygenation during normal healing in an equine model of cutaneous fibroproliferative disorder. Wound Repair Regen 19:89–97

12. Chen JJ, Zhao S, Cen Y, et al (2007) Effect of heat shock protein 47 on collagen accumulation in keloid fibroblast cells. Br J Dermatol 156:1188–1195

13. Chiu LL, Sun CH, Yeh AT, et al (2005) Photodynamic therapy on keloid fibroblasts in tissue-engineered keratinocyte-fibroblast co-culture. Lasers Surg Med 37:231–244

14. Chua AWC, Ma D, Gan SU, et al (2011) The role of R-spondin2 in keratinocyte proliferation and epidermal thickening in keloid scarring. J Invest Dermatol 131:644–654

15. Cicchi R, Kapsokalyvas D, Giorgi V De, et al (2010) Scoring of collagen organization in healthy and diseased human dermis by multiphoton microscopy. J Biophotonics 3:34–43

16. Da Costa V, Wei R, Lim R, et al (2008) Nondestructive imaging of live human keloid and facial tissue using multiphoton microscopy. Arch Facial Plast Surg 10:38–43

17. Craig PRD, Schofield JD, Jackson SS (1975) Collagen biosynthesis in normal and hypertrophic scars and keloid as a function of the duration of the scar. Br J Surg 62:741–744

18. Do D V., Ong CT, Khoo YT, et al (2012) Interleukin-18 system plays an important role in keloid pathogenesis via epithelial-mesenchymal interactions. Br J Dermatol 166:1275–1288

19. Dohi T, Padmanabhan J, Akaishi S, et al (2019) The interplay of mechanical stress, strain, and stiffness at the keloid periphery correlates with increased caveolin-1/ROCK signaling and scar progression. Plast Reconstr Surg 144:58–67

20. Dohi T, Miyake K, Aoki M, et al (2015) Tissue inhibitor of metalloproteinase-2 suppresses collagen synthesis in cultured keloid fibroblasts. Plast Reconstr Surg - Glob Open 3:e520

21. Duong HS, Zhang Q, Kobi A, et al (2006) Assessment of morphological and immunohistological alterations in long-term keloid skin explants. Cells Tissues Organs 181:89–102

22. Elrefaie AM, Salem RM, Faheem MH (2019) High-resolution ultrasound for keloids and hypertrophic scar assessment. Lasers Med Sci [Epub ahead of print]

23. Estrem SA, Domayer M, Bardach J, Cram AE (1987) Implantation of human keloid into athymic mice. Laryngoscope 97:1214–1218

24. Fujiwara M, Muragaki Y, Ooshima A (2005) Upregulation of transforming growth factor-β1 and vascular endothelial growth factor in cultured keloid fibroblasts: relevance to angiogenic activity. Arch Dermatol Res 297:161–169

25. Funayama E, Chodon T, Oyama A, Sugihara T (2003) Keratinocytes promote proliferation and inhibit apoptosis of the underlying fibroblasts: an important role in the pathogenesis of keloid. J Invest Dermatol 121:1326–1331

26. Hinek A, Kim HJ, Wang Y, et al (2014) Sodium l-ascorbate enhances elastic fibers deposition by fibroblasts from normal and pathologic human skin. J Dermatol Sci 75:173–182

27. Hochman B, Cássia F, Bôas V, et al (2005) Keloid heterograft in the hamster (Mesocricetus auratus) cheek pouch. Acta Cirúrgica Bras 20:200–212

28. Ichioka S, Ando T, Shibata M, Sekiya N (2008) Oxygen consumption of keloids and hypertrophic Scars. Ann Plast Surg 60:194–197

29. Ishiko T, Naitoh M, Kubota H, et al (2013) Chondroitinase injection improves keloid pathology by reorganizing the extracellular matrix with regenerated elastic fibers. J Dermatol 40:380–383

30. Jin Q, Gui L, Niu F, et al (2018) Macrophages in keloid are potent at promoting the differentiation and function of regulatory T-cells. Exp Cell Res 362:472–476

31. Khoo YT, Ong CT, Mukhopadhyay A, et al (2006) Upregulation of secretory connective tissue growth factor (CTGF) in keratinocyte-fibroblast coculture contributes to keloid pathogenesis. J Cell Physiol 208:336–343

32. Khorshid FA (2005) Comparative study of keloid formation in humans and laboratory animals. Med Sci Monit 11:BR212-219

33. Kischer CW, Sheridan D, Pindur J (1989) Use of nude (athymic) mice for the study of hypertrophic scars and keloids: vascular continuity between mouse and implants. Anat Rec 225:189–196

34. Kuwahara H, Tosa M, Murakami M, et al (2016) Examination of epithelial mesenchymal transition in keloid tissues and possibility of keloid therapy target. Plast Reconstr Surg Glob Open 4:1–7

35. Lee SS, Yosipovitch G, Chan YH, Goh CL (2004) Pruritus, pain, and small nerve fiber function in keloids: a controlled study. J Am Acad Dermatol 51:1002–1006

36. Lee WJ, Kim YO, Choi IK, et al (2011) Adenovirus-relaxin gene therapy for keloids: implication for reversing pathological fibrosis. Br J Dermatol 165:673–677

37. Lee Y-S, Hsu T, Chiu W-C, et al (2015) Keloid-derived, plasma/fibrin-based skin equivalents generate de novo dermal and epidermal pathology of keloid fibrosis in a mouse model. Wound Repair Regen 24:302–316

38. Lee YS, Liang YC, Wu P, et al (2019) STAT3 signalling pathway is implicated in keloid pathogenesis by preliminary transcriptome and open chromatin analyses. Exp Dermatol 28:480–484

39. Li J, Fu R, Li L, et al (2014) Co-delivery of dexamethasone and green tea polyphenols using electrospun ultrafine fibers for effective treatment of keloid. Pharm Res 31:1632–1643

40. Liang C, Yen Y, Hung L, et al (2013) Thalidomide inhibits fibronectin production in TGF-β1-treated normal and keloid fibroblasts via inhibition of the p38/SMAD3 pathway. Biochem Pharmacol 85:1594–1602

41. Liao WT, Yu HS, Arbiser JL, et al (2010) Enhanced MCP-1 release by keloid CD14+ cells augments fibroblast proliferation: role of MCP-1 and Akt pathway in keloids. Exp Dermatol 19:e142–e150

42. Lim CP, Phan TT, Lim IJ, Cao X (2009) Cytokine profiling and Stat3 phosphorylation in epithelial-mesenchymal interactions between keloid keratinocytes and fibroblasts. J Invest Dermatol 129:851–861

43. Lim CK, Halim AS, Yaacob NS, et al (2013) Keloid pathogenesis via Drosophila similar to mothers against decapentaplegic (SMAD) signaling in a primary epithelial-mesenchymal in vitro model treated with biomedical-grade chitosan porous skin regenerating template. J Biosci Bioeng 115:453–458

44. Lim IJ, Phan TT, Song C, et al (2001) Investigation of the influence of keloid-derived keratinocytes on fibroblast growth and proliferation in vitro. Plast. Reconstr. Surg. 107:787–808

45. Lim IJ, Phan TT, Tan EK, et al (2003) Synchronous activation of ERK and phosphatidylinositol 3-kinase pathways is required for collagen and extracellular matrix production in keloids. J Biol Chem 278:40851–40858

46. Lim IJ, Phan T-T, Bay B-H, et al (2002) Fibroblasts cocultured with keloid keratinocytes: normal fibroblasts secrete collagen in a keloidlike manner. Am J Physiol Cell Physiol 283:C212–C222

47. Limandjaja GC, Belien JM, Scheper RJ, et al (2019) Hypertrophic and keloid scars fail to progress from the CD34-/α-smooth muscle actin (α-SMA)+ immature scar phenotype and show gradient differences in α-SMA and p16 expression. Br J Dermatol Jun 17:[Epub ahead of print]

48. Limandjaja GC, van den Broek LJ, Breetveld M, et al (2018) Characterization of in vitro reconstructed human normotrophic, hypertrophic, and keloid scar models. Tissue Eng - Part C Methods 24:242–253

49. Limandjaja GC, Waaijman T, Roffel S, et al (2019) Monocytes co-cultured with reconstructed keloid and normal skin models skew towards M2 macrophage phenotype. Arch Dermatol Res 311:615–627

50. Limandjaja GC, Broek LJ van den, Waaijman T, et al (2018) Reconstructed human keloid models show heterogeneity within keloid scars. Arch Dermatol Res 310:815–826

51. Lin L, Wang Y, Liu W, Huang Y (2015) BAMBI inhibits skin fibrosis in keloid through suppressing TGF-β1-induced hypernomic fibroblast cell proliferation and excessive accumulation of collagen I. Int J Clin Exp Med 8:13227–13234

52. Liu J, Ren J, Su L, et al (2017) Human adipose tissue-derived stem cells inhibit the activity of keloid fibroblasts and fibrosis in a keloid model by paracrine signaling. Burns 44:370–385

53. Liu Q, Wang X, Jia Y, et al (2016) Increased blood flow in keloids and adjacent skin revealed by laser speckle contrast imaging. Lasers Surg Med 48:360–364

54. Ma H, Xu R, Cheng H, et al (2003) Gene transfer into human keloid tissue with adeno-associated virus vector. J Trauma 54:569–73

55. Mancini RE, Quaife J V (1962) Histogenesis of experimentally produced keloids. J Invest Dermatol 38:143–181

56. Mendoza-Garcia J, Sebastian A, Alonso-Rasgado T, Bayat A (2015) Ex vivo evaluation of the effect of photodynamic therapy on skin scars and striae distensae. Photodermatol Photoimmunol Photomed 31:239–251

57. Milsom JP, Craig RDP (1973) Collagen degradation in cultured keloid and hypertrophic scar tissue. Br J Dermatol 89:635–644

58. Miragliotta V, Pirone A, Donadio E, et al (2016) Osteopontin expression in healing wounds of horses and in human keloids. Equine Vet J 48:72–77

59. Miragliotta V, Ipiña Z, Lefebvre-lavoie J, et al (2008) Equine CTNNB1 and PECAM1 nucleotide structure and expression analyses in an experimental model of normal and pathological wound repair. BMC Physiol 8:1–12

60. Miragliotta V, Lefebvre-Lavoie J, Lussier JG, Theoret CL (2008) Equine ANXA2 and MMP1 expression analyses in an experimental model of normal and pathological wound repair. J Dermatol Sci 51:103–112

61. Miragliotta V, Raphäel K, Lussier JG, Theoret CL (2009) Equine lumican (LUM) cDNA sequence and spatio-temporal expression in an experimental model of normal and pathological wound healing. Vet Dermatol 20:243–248

62. Mitts TF, Bunda S, Wang Y, Hinek A (2010) Aldosterone and mineralocorticoid receptor antagonists modulate elastin and collagen deposition in human skin. J Invest Dermatol 130:2396–2406

63. Morris DE, Wu L, Zhao LL, et al (1997) Acute and chronic animal models for excessive dermal scarring: Quantitative studies. Plast. Reconstr. Surg. 100:674–681

64. Mukhopadhyay A, Do D V., Ong CT, et al (2011) The role of stem cell factor and c-KIT in keloid pathogenesis: do tyrosine kinase inhibitors have a potential therapeutic role? Br J Dermatol 164:372–386

65. Mukhopadhyay A, Tan EKJ, Khoo YTA, et al (2005) Conditioned medium from keloid keratinocyte/keloid fibroblast coculture induces contraction of fibroblast-populated collagen lattices. Br J Dermatol 152:639–645

66. Mukhopadhyay A, Khoo A, Cheong HH, et al (2007) Targeting of Sp1 transcription factor: a novel therapeutic approach for keloids, an in vitro analysis. Exp Dermatol 16:1023–1031

67. Mukhopadhyay A, Fan S, Dang VD, et al (2010) The role of hepatocyte growth factor/c-Met system in keloid pathogenesis. J Trauma - Inj Infect Crit Care 69:1457–1466

68. Mukhopadhyay A, Wong MY, Chan SY, et al (2010) Syndecan-2 and decorin: proteoglycans with a difference-implications in keloid pathogenesis. J Trauma - Inj Infect Crit Care 68:999–1008

69. Murao N, Seino K ichiro, Hayashi T, et al (2014) Treg-enriched CD4+ T cells attenuate collagen synthesis in keloid fibroblasts. Exp Dermatol 23:266–271

70. Nagasao T, Aramaki-Hattori N, Shimizu Y, et al (2013) Transformation of keloids is determined by stress occurrence patterns on peri-keloid regions in response to body movement. Med Hypotheses 81:136–141

71. Noishiki C, Takagi G, Kubota Y, Ogawa R (2017) Endothelial dysfunction may promote keloid growth. Wound Repair Regen 25:976–983

72. Ong CT, Khoo YT, Mukhopadhyay A, et al (2007) mTOR as a potential therapeutic target for treatment of keloids and excessive scars. Exp Dermatol 16:394–404

73. Ong CT, Khoo YT, Tan EK, et al (2007) Epithelial–mesenchymal interactions in keloid pathogenesis modulate vascular endothelial growth factor expression and secretion. J Pathol 211:95–108

74. Ooi BNS, Mukhopadhyay A, Masilamani J, et al (2010) Hepatoma-derived growth factor and its role in keloid pathogenesis. J Cell Mol Med 14:1328–1337

75. Ozawa T, Okamura T, Harada T, et al (2006) Accumulation of glucose in keloids with FDG-PET. Ann Nucl Med 20:41–44

76. Park G, Yoon BS, Moon JH, et al (2008) Green tea polyphenol epigallocatechin-3-gallate suppresses collagen production and proliferation in keloid fibroblasts via inhibition of the STAT3-signaling pathway. J Invest Dermatol 128:2429–2441

77. Phan TT, Lim IJ, Bay BH, et al (2003) Role of IGF system of mitogens in the induction of fibroblast proliferation by keloid-derived keratinocytes in vitro. Am J Physiol Cell Physiol 284:C860–C869

78. Phan TT, Lim IJ, Aalami O, et al (2005) Smad3 signalling plays an important role in keloid pathogenesis via epithelial-mesenchymal interactions. J Pathol 207:232–242

79. Phan TT, Lim IJ, Bay BH, et al (2002) Differences in collagen production between normal and keloid-derived fibroblasts in serum-media co-culture with keloid-derived keratinocytes. J Dermatol Sci 29:26–34

80. Philandrianos C, Bertrand B, Andrac-Meyer L, et al (2015) Treatment of keloid scars with a 1210-nm diode laser in an animal model. Lasers Surg Med 47:798–806

81. Plikus MV, Guerrero-Juarez CF, Ito M, et al (2017) Regeneration of fat cells from myofibroblasts during wound healing. Science 355:748–752

82. Ring HC, Mogensen M, Hussain AA, et al (2015) Imaging of collagen deposition disorders using optical coherence tomography. J Eur Acad Dermatology Venereol 29:890–898

83. Sato H, Suzuki A, Funahashi M, et al (1996) Characteristics of growth, morphology, contractility, and protein expression of fibroblasts derived from keloid. Wound Repair Regen 4:103–114

84. Shang T, Yao B, Gao D, et al (2018) A novel model of humanised keloid scarring in mice. Int Wound J 15:90–94

85. Shetlar MR, Shetlar CL, Hendricks L, Kischer CW (1985) The use of athymic nude mice for the study of human keloids. Exp Biol Med 179:549–552

86. Shetlar MR, Shetlar CL, Kischer CW, Pindur J (1991) Implants of keloid and hypertrophic scars into the athymic nude mouse: changes in the glycosaminoglycans of the implants. Connect Tissue Res 26:23–36

87. Shetlar MR, Shetlar DJ, Bloom RF, et al (1998) Involution of keloid implants in athymic mice treated with pirfenidone or with triamcinolone. J Lab Clin Med 132:491–496

88. Smith CJ, Smith JC, Finn MC (1987) The possible role of mast cells (allergy) in the production of keloid and hypertrophic scarring. J Burn Care Rehabil 8:126–31

89. Suetake T, Sasai S, Zhen YX, et al (1996) Functional analyses of the stratum corneum in scars. Sequential studies after injury and comparison among keloids, hypertrophic scars, and atrophic scars. Arch Dermatol 132:1453–1458

90. Sunaga A, Kamochi H, Sarukawa S, et al (2017) Experimental reconstitution of human keloids in mouse skin. Plast Reconstr Surg Glob Open 5:1–7

91. Supp DM, Hahn JM, Glaser K, et al (2012) Deep and superficial keloid fibroblasts contribute differentially to tissue phenotype in a novel in vivo model of keloid scar. Plast Reconstr Surg 129:1259–1271

92. Supp DM, Glaser K, Hahn JM, et al (2012) Abnormal responses of keloid tissue to wounding identified using in vitro model system. Eplasty 12:184–187

93. Syed F, Bayat A (2012) Notch signaling pathway in keloid disease: enhanced fibroblast activity in a Jagged-1 peptide-dependent manner in lesional vs. extralesional fibroblasts. Wound Repair Regen 20:688–706

94. Torkian BA, Yeh AT, Engel R, et al (2004) Modeling aberrant wound healing using tissue-engineered skin constructs and multiphoton microscopy. Arch Facial Plast Surg 6:180–187

95. Tseng S-H, Hsu C-K, Yu-Yun Lee J, et al (2012) Noninvasive evaluation of collagen and hemoglobin contents and scattering property of in vivo keloid scars and normal skin using diffuse reflectance spectroscopy: pilot study. J Biomed Opt 17:0770051

96. Waki EY, Crumley RL, Jakowatz JG (1991) Effects of pharmacologic agents on human keloids implanted in athymic mice: a pilot study. Arch Otolaryngol – Head Neck Surg 117:1177–1181

97. Wang H, Luo S (2013) Establishment of an animal model for human keloid scars using tissue engineering method. J Burn Care Res 34:439–446

98. Wang X, Liu K, Ruan M, et al (2018) Gallic acid inhibits fibroblast growth and migration in keloids through the AKT/ERK signaling pathway. Acta Biochim Biophys Sin 50:1114–1120

99. Wang X, Smith P, Pu LLQ, et al (1999) Exogenous transforming growth factor β2 modulates collagen I and collagen III synthesis in proliferative scar xenografts in nude rats. J Surg Res 87:194–200

100. Xia W, Phan T-T, Lim IJ, et al (2004) Complex epithelial-mesenchymal interactions modulate transforming growth factor-beta expression in keloid-derived cells. Wound Repair Regen 12:546–556

101. Yagi Y, Muroga E, Naitoh M, et al (2013) An ex vivo model employing keloid-derived cell-seeded collagen sponges for therapy development. J Invest Dermatol 133:386–393

102. Yoo MG, Kim IH (2014) Keloids and hypertrophic scars: characteristic vascular structures visualized by using dermoscopy. Ann Dermatol 26:603–609

103. Yu D, Shang Y, Yuan J, et al (2016) Wnt/β-catenin signaling exacerbates keloid cell proliferation by regulating telomerase. Cell Physiol Biochem 39:2001–2013

104. Zhang Q, Kelly AP, Wang L, et al (2006) Green tea extract and (−)-epigallocatechin-3-gallate inhibit mast cell-stimulated type I collagen expression in keloid fibroblasts via blocking PI-3K/Akt signaling pathways. J Invest Dermatol 126:2607–2613

105. Zhang Q, Yamaza T, Kelly AP, et al (2009) Tumor-like stem cells derived from human keloid are governed by the inflammatory niche driven by IL-17/IL-6 axis. PLoS One 4:e7798
